# Comprehensive bioinformatics analysis unveils THEMIS2 as a carcinogenic indicator related to immune infiltration and prognosis of thyroid cancer

**DOI:** 10.1038/s41598-024-58943-6

**Published:** 2024-04-08

**Authors:** Jun-feng Liu, Bing Zou, Cheng Xiang, Hai-chao Yan

**Affiliations:** 1 Head and Neck Breast Department, Xinxiang Central Hospital, The Fourth Clinical College of Xinxiang Medical University, Xinxiang, 453000 Henan China; 2 Breast and Nail Surgery, Feicheng City People’s Hospital, Feicheng, 271600 Shandong China; 3 Department of Thyroid Surgery, The Second Affiliated Hospital, Zhejiang University School of Medicine, No. 88, Jiefang Road, Shangcheng District, Hangzhou, 310009 Zhejiang China

**Keywords:** THEMIS2, Thyroid cancer, Prognosis, Immune infiltration, Cancer, Thyroid cancer

## Abstract

The aim of this study was to identify biomarkers associated with the initiation and prognosis of thyroid cancer and elucidate the underlying pathogenic mechanisms. We obtained expression profiles and clinical information from the Cancer Genome Atlas (TCGA)-THCA and three datasets (GSE53157, GSE82208, and GSE76039). The three microarray datasets were combined using Perl and the sva package in R and termed ‘merged dataset’. Weighted gene co-expression network analysis (WGCNA) identified 15 gene co-expression modules in the merged dataset and 235 hub genes. Venn diagram analysis revealed 232 overlapping genes between the merged and THCA datasets. Overlapping genes were subjected to gene ontology (GO) and Kyoto Encyclopedia of Genes and Genomes (KEGG) pathway enrichment analyses. The least absolute shrinkage and selection operator (LASSO) regression identified THEMIS2 as a candidate hub gene. Cox, Kaplan–Meier (K–M) survival and gene set enrichment analysis (GSEA) confirmed the correlation of THEMIS2 with overall survival, its enrichment in immunologic processes, and its association with the p53 and JAK-STAT signaling pathways. Its expression was positively correlated with those of immune checkpoints and the infiltration level of immune cells. Receiver operating characteristic curve (ROC) analysis confirmed that THEMIS2, a diagnostic biomarker, could distinguish between tumor and normal specimens. The nomogram (ROC or DCA) model containing THEMIS2, age, and stage predicted favourable prognoses. Thus, THEMIS2 was a biomarker of immune infiltration and prognosis in thyroid cancer.

## Introduction

The thyroid gland, located at the base of the neck, is butterfly-shaped and secretes thyroid hormones that maintain the body’s metabolic equilibrium and regulate heart rate, blood pressure, body temperature, and weight^1^. Thyroid cancer is a commonly occurring malignant head and neck tumor arising from the thyroid follicular epithelium or specific epithelial cells^2^. The incidence of thyroid cancer is rising worldwide. The incidence of thyroid cancer is projected to increase by 20 percent per year in China^3^. Different types of thyroid cancer have been classified based on the tumor’s origin and differentiation. Papillary thyroid carcinoma (PTC), accounting for approximately 90% of all cases, is the most prevalent type. PTC and follicular thyroid carcinoma (FTC) are collectively termed differentiated thyroid carcinoma (DTC), and these patients show favorable prognosis, with a 5-year survival rate of over 98%. In contrast, anaplastic thyroid cancer (ATC) is extremely malignant, with a median survival time of only 7–10 months and a 5-year survival rate of approximately 8%. Medullary thyroid carcinoma (MTC) has an intermediate prognosis between DTC and ATC, with a 5-year survival rate of approximately 90%^4^. The 10-year recurrence rate for patients with thyroid cancer following treatment ranges from 1.7–22.7%^5^. The incidence and recurrence of thyroid cancer continue to increase. Consequently, identifying specific tumor markers is essential for comprehending the pathogenesis and progression of this disease and developing new therapeutic strategies.

Existing evidence indicates that the tumor microenvironment (TME) is crucial for malignant development^6^. Tumor immune microenvironment (TIME) comprises immune-related components in the TME^7^. TIME is classified into the following three subtypes based on the distribution and categories of immune cells: immune-excluded (I-E), immune-inflamed (I-I), and immune-desert (I-D). TIME classification assists in elucidating the selective effects of immunotherapy and guides the selection of clinical treatment approaches. Different cases and tumor sites contribute to the heterogeneity of TIME^8^. Therefore, understanding the interaction between thyroid cancer and its immune microenvironment is crucial for developing effective treatment targets and new cancer prognostic markers.

In this study, Gene Expression Omnibus (GEO) and the Cancer Genome Atlas (TCGA) databases were analyzed to identify potential diagnostic and prognostic biomarkers for thyroid cancer. Various bioinformatics methods were used to analyze the relevant genes. The correlation between gene expression and prognosis in patients with thyroid cancer was analyzed using clinical data obtained from the TCGA-THCA dataset. Enrichment analyses were conducted to elucidate the molecular mechanisms. The relationship between gene expression, immune cell infiltration, and immune checkpoint was also assessed. We identified a novel prognostic marker and potential therapeutically efficacious target for thyroid cancer.

## Methods

### Study design

**Figure 1:**
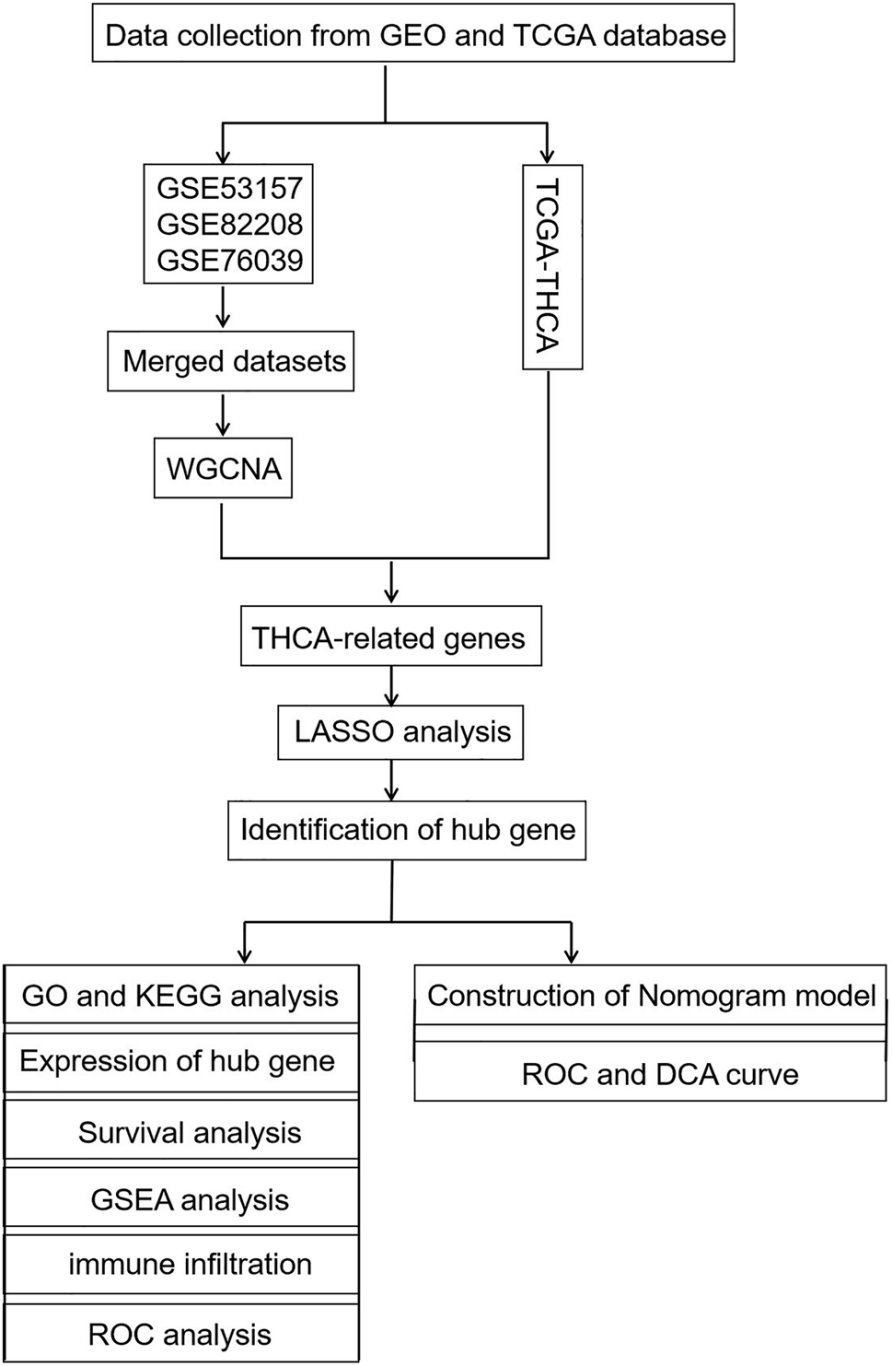
General workflow of the study.

This was an integrated bioinformatics analysis aimed at assessing multiple gene expression datasets to identify THCA-related biomarker(s). The general workflow is shown in Fig. 1.

### GEO data collection, preprocessing, and analysis

The GEO database was queried using the keyword, “thyroid cancer.” All samples were from humans. The dataset contained at least 25 samples. Three microarray data sets (GSE53157, GSE82208, and GSE76039) were selected and downloaded. Detailed information on the datasets is provided in Supplementary Table 1. We first removed batch effects using the ‘Combat’ function in the ‘sva’ package in R and used Perl and “sva”^9^ to merge the three datasets.

### Weighted gene co-expression network analysis (WGCNA) for the merged dataset from the GEO database

WGCNA is a data reduction and unsupervised classification method. First, the co-expression network was built using the “WGCNA” package in R^10^ using the expression profile of the merge matrix. The parameters set for the network construction were as follows: mergeCutHeight = 0.25, minModuleSize = 200, corType = “Pearson”. Key genes within each co-expression module correlated with clinical features (age, ATC, and PDTC) were determined. After gene clustering, a heatmap showed the correlation between modules and phenotypes.

### Data downloading and preprocessing from TCGA

We obtained RNA sequencing data and clinical information on THCA from TCGA. The dataset included 510 tumor samples, 58 normal samples, and their corresponding clinical information, such as the follow-up time and clinical status. We transformed the raw TCGA counts data by collating the data and transforming the IDs to obtain a TCGA expression matrix.

### Identification of THCA-related genes

Venn diagram analysis was performed to identify overlapping genes between the key genes from GEO and the TCGA-THCA dataset (https://bioinformatics.psb.ugent.be/webtools/Venn/).

### Gene Ontology (GO) and Kyoto Encyclopedia of Genes and Genomes (KEGG) analyses for the overlapping genes

The “clusterProfiler” package^11^ in R was used for GO annotation and KEGG pathway enrichment analyses of the overlapped genes. GO terms were of three types: biological process (BP), molecular function (MF), and cellular component (CC). P < 0.05 and false discovery rate (FDR) q < 0.05 represented a statistically significant enrichment.

### Development and validation of hub genes in thyroid cancer

We performed a preliminary analysis of the overlapping genes. Least absolute shrinkage and selection operator (LASSO) regression analysis was performed to identify hub genes correlated with the development of THCA. The risk score was calculated using the following formula: risk score = $\sum\nolimits_{n = 1}^{j} {{\text{Coef}}\;{\text{j}}*{\text{Xj}}}$^12^, with Coef j referring to the coefficient calculated using the LASSO method and Xj representing gene expression. We investigated the potential diagnostic value of the hub genes for distinguishing thyroid cancer from normal samples using receiver operating characteristic (ROC) curve analysis using the pROC function in R. This allowed us to calculate the area under the curve (AUC) when screening signature genes and estimating their corresponding diagnostic value. Only THEMIS2 was identified as a key hub gene.

### Kaplan‒Meier (K–M) survival analysis

Within the scope of this investigation was the consideration of overall survival (OS) data in TCGA-THCA. All patients were divided into high- and low-expression groups according to the best cutoff value of THEMIS2 expression. A K–M survival plot was generated using the “maxstat” function in R to explore the survival difference between the two groups. K–M survival analysis was performed among subgroups to explore the detailed association between THEMIS2 expression and prognosis.

### Gene set enrichment analysis (GSEA) for THEMIS2

The reference gene set, “c2.cp.kegg.v7.4.symbols.gmt,” was selected, and the GSEA software (version 4.1.0) was used to compare significantly different pathways between the low- and high-expression groups of TCGA-THCA. The criterion for significant enrichment was a p-value of less than 0.05.

### THEMIS2 expression and localization

THEMIS2 protein levels and its subcellular localization were determined using the Human Protein Atlas (HPA) database (https://www.proteinatlas.org). Its expression pattern was explored based on the TCGA-THCA dataset stratified by age, sex, and clinical stage.

### Immune analyses for THEMIS2

The CIBERSORT algorithm was used to obtain the infiltration levels of 22 immune cells in each patient based on the TCGA-THCA gene expression profile. The “limma” tool in R software was utilized to determine the differences in the levels of cellular infiltration between THEMIS2 high- and low-expression groups. The correlation between THEMIS2 expression and immune checkpoints was evaluated.

### Assessment of THEMIS2 as a prognostic value for thyroid cancer

Both univariate and multivariate Cox regression analyses were performed to determine independent prognostic factors associated with OS. Hub gene expression was a covariate, along with age, sex, and stage. Hazard ratios (HRs) were used to identify protective factors (HR < 1) or risk factors (HR > 1). The effect of related variables on the association between THEMIS2 and OS was evaluated by Cox regression analysis.

### Establishment and validation of the nomogram model

Data from TCGA-THCA were used to establish a nomogram model in this study. The model was developed based on three parameters, including age, stage, and THEMIS2 expression. The ROC curves for the nomogram model were generated. Simultaneously, decision curve analysis (DCA) was used to assess the net benefit of nomograms in the clinical context.

### Cell lines

The human papillary thyroid cancer (PTC) cell line, TPC-1 (BNCC, Beijing, China), and the normal human thyroid cell line, HTori-3 (ATCC, Manassas, VA, USA) were cultured in Dulbecco’s modified Eagle’s medium (DMEM) (Gibco, USA) supplemented with 10% fetal bovine serum (FBS), 100 U/ml penicillin, and 100 mg/ml streptomycin. The cells were incubated in a 5% CO_2_ incubator at 37 °C. When the cells reached 70–80% confluence, they were passaged, in accordance with standard procedures.

### RT-PCR

Total RNA was extracted from cultured cell lines by using Trizol Reagent (TaKaRa Bio) according to the manufacturer’s instructions. cDNA synthesis was performed using TransScript One-Step gDNA Removal and cDNA Synthesis SuperMix (TRAN) according to the protocol described, and RT-PCR was finished by using 2× EasyTaq PCR SuperMix (TRAN) as recommended by the manufacturer. The sequences were as follows: for THEMIS2 (forward: 5′-CTTCCAGGGCTACTTCACCC-3′, reverse: 5′-TTCCTCCTCATCCTCCCCAG-3′); for GAPDH (forward: 5′-ACCCAGAAGACTGTGGATGG-3′, reverse: 5′-TTGTGCTGAAGTCTCCCCATC-3′).

### Statistical analysis

The expressional difference of hub genes was analyzed using a t-test for two groups, and analysis of variance (ANOVA) was performed for multiple groups. The correlation between two continuous variables was analyzed using the Pearson method. The survival difference between the two groups was compared with a log-rank test. Multiplicative interaction terms were used to calculate the relative excess risk of interaction (RERI), which in turn was used to analyze the effect of the interaction. SPSS version 23.0 and R software were utilized for all statistical analyses. P < 0.05 represented a statistically significant result.

### Ethics approval

The Ethics Committee of Xinxiang Central Hospital/ The Fourth Clinical College of Xinxiang Medical University deemed that this research is based on open-source data, so the need for ethics approval was waived.

## Results

### WGCNA for the merged dataset of thyroid cancer

**Figure 2:**
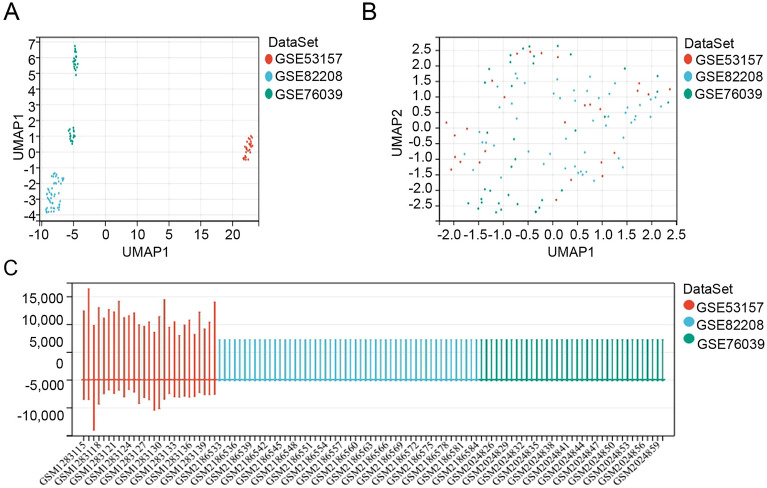
Normalization and merging of the three datasets. (**A**) Three datasets. (**B**) Merging the three datasets. (**C**) Normalization of the three datasets.

**Figure 3:**
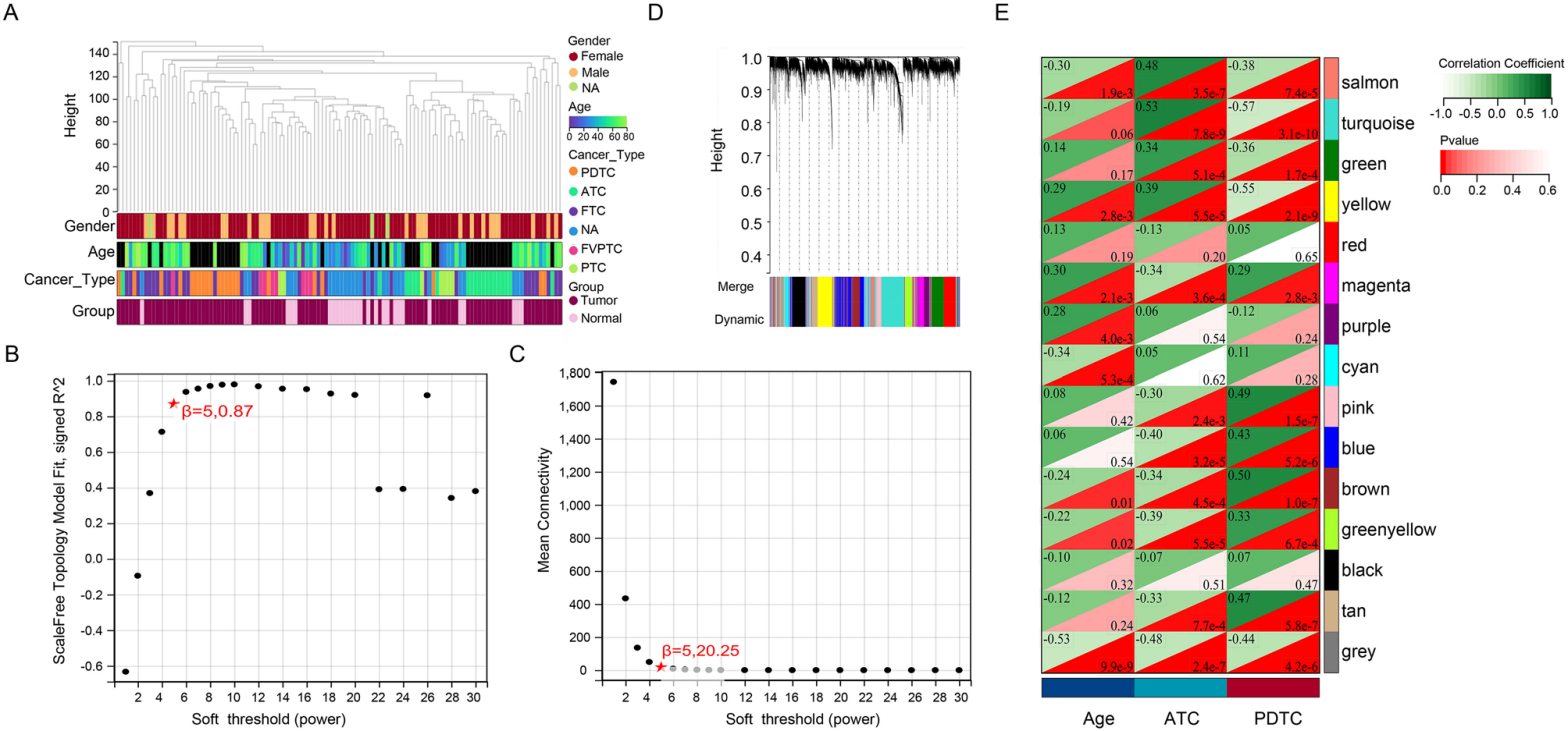
WGCNA for the merged datasets. (**A**) Clustering dendrogram for 116 samples. (**B**) Analysis of the scale-free index at various soft-threshold powers (β). (**C**) Mean connectivity for various soft-threshold powers. (**D**) Clustering dendrograms. Based on dynamic tree cutting, the genes were clustered into different modules through hierarchical clustering with a threshold of 0.25. Each color represents a module. (**E**) Correlation heatmap between module eigengenes and clinical traits.

Three datasets (GSE53157, GSE82208, and GSE76039) related to thyroid cancer (Fig. 2A) were merged (Fig. 2B) to increase sample size and decrease heterogeneity, and data were normalized (Fig. 2C). WGCNA was performed for the merged data to acquire a list of genes with frequently high co-expression and strong topological overlap similarity. An expression matrix for the merged dataset included 116 samples (28 control samples and 88 tumor samples). Figure 3A depicts a sample clustering tree based on Pearson’s correlation coefficient for sample aggregation. We selected a soft threshold of five based on the scale-free topology criterion with an R^2^ value of 0.87 to obtain a scale-free network. The adjacency matrix was transformed into a TOM matrix (Fig. 3B,C), illustrating the similarity between nodes based on the weighted correlation. Fifteen modules were obtained using average hierarchical clustering and dynamic tree trimming (Fig. 3D). The correlation between each gene among the 15 models and clinical traits (age, ATC, and PDTC) were evaluated, and a total of 235 key genes with MM > 0.8 and P value < 0.0001 were identified.

### GO and KEGG analyses for overlapping genes

**Figure 4:**
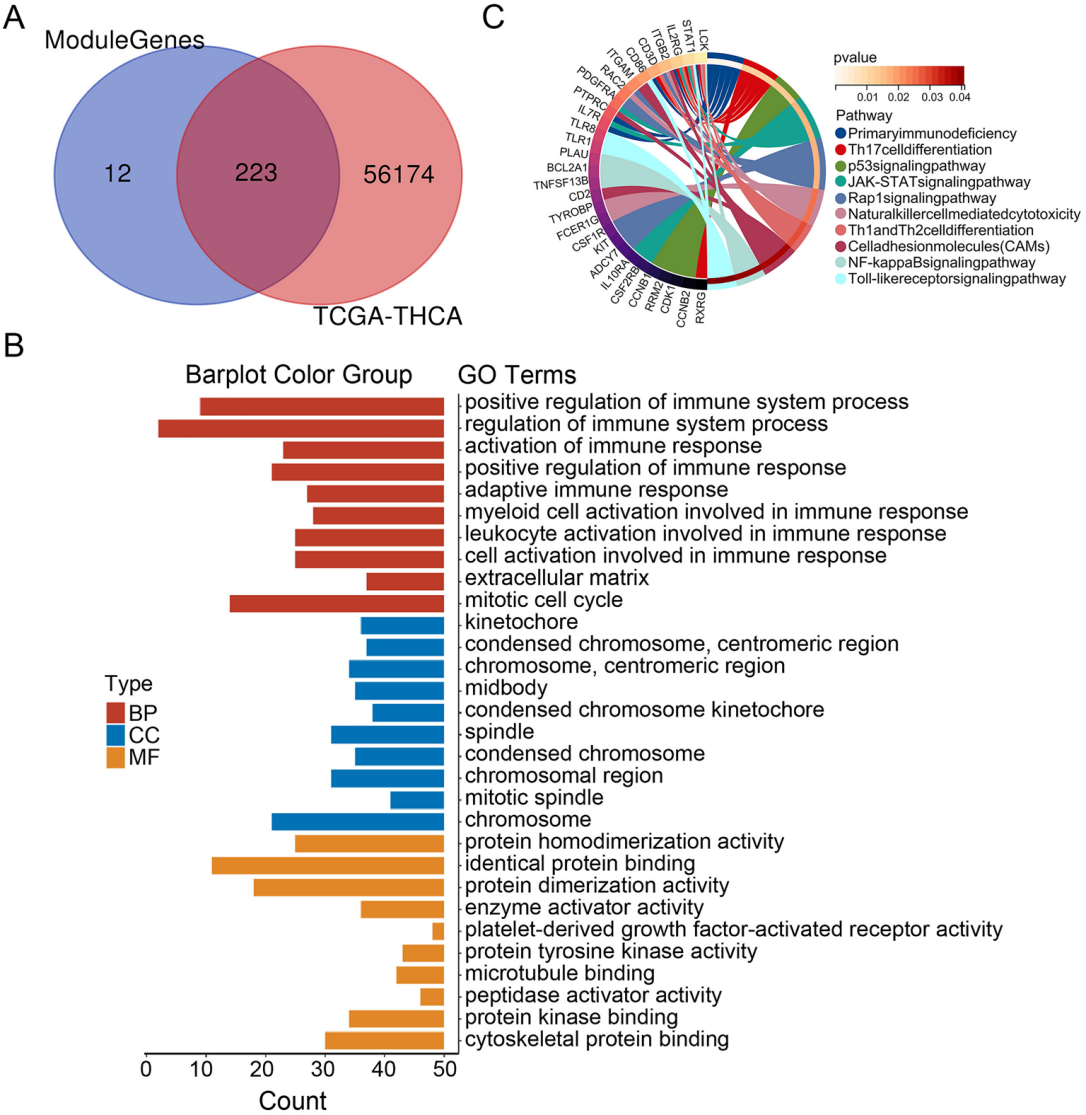
GO and KEGG analyses for overlapping genes. (**A**) Identification of overlapping genes from the Venn diagram. (**B**) GO analysis for the overlapping genes. (**C**) KEGG analysis for the overlapping genes.

Whole genes from the TCGA-THCA dataset were used as candidates to expand the search scope of genes. Among 235 phenotype-related genes, 223 were common to the TCGA-THCA data set based on Venn diagram analysis (Fig. 4A). We performed GO annotation and KEGG pathway enrichment analysis for the overlapping genes. These genes were correlated to the regulation of the immune system process, kinetochore, and protein homodimerization activity (Fig. 4B). KEGG pathway enrichment analysis suggested that the genes were involved in the Toll-like receptor signaling, p53 signaling, JAK-STAT signaling, and Toll-like receptor signaling (Fig. 4C).

### THEMIS2 is a reliable diagnostic biomarker

**Figure 5:**
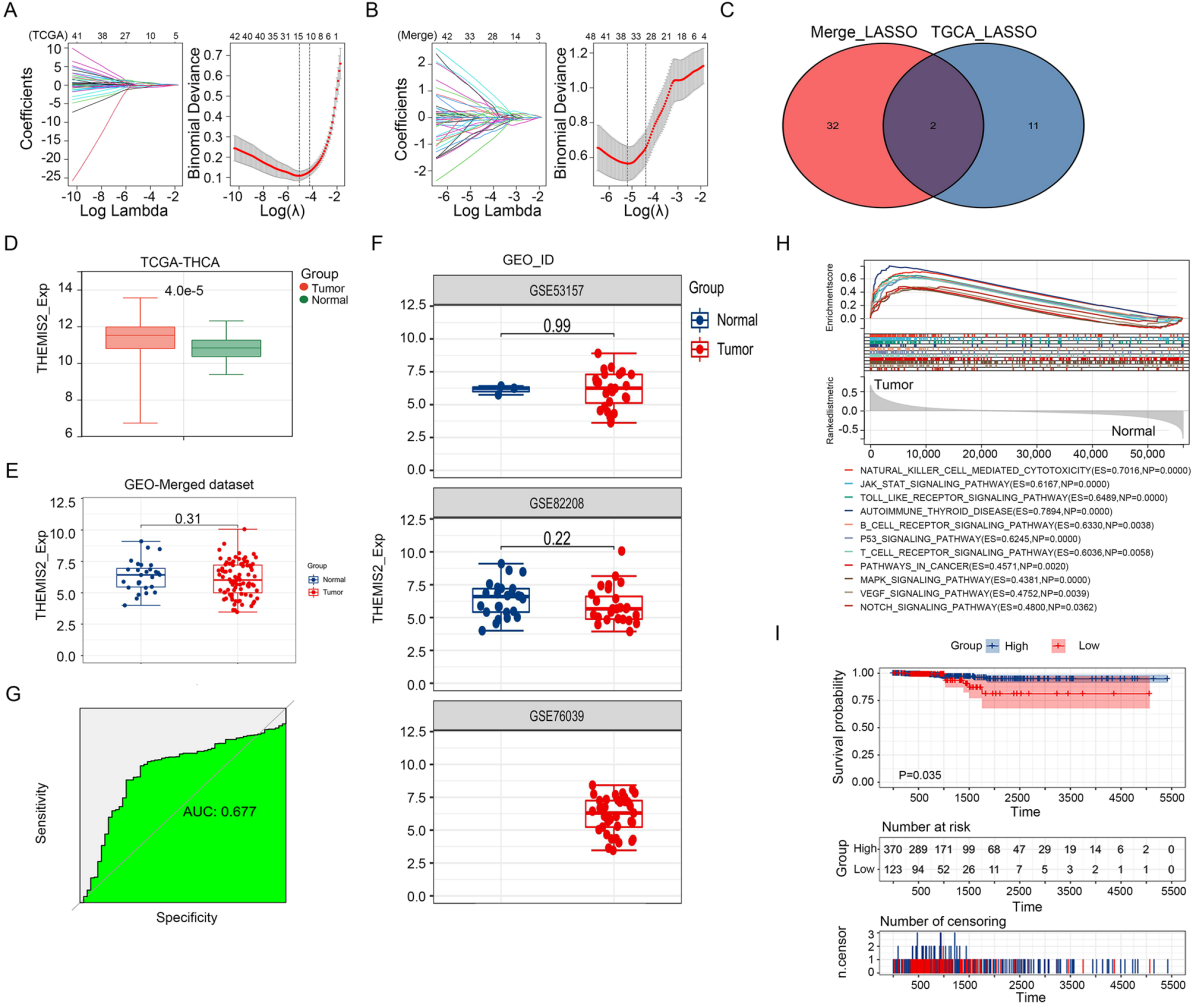
Identification of key genes associated with diagnosis and prognosis of thyroid cancer. (**A**) Expression signatures of 13 genes from the TCGA-THCA dataset based on THCA-related clusters selected in the LASSO models (left). Cross-validation for tuning parameter selection in the LASSO model (right). (**B**) Expression signatures of 34 genes from the merged dataset based on THCA-related clusters selected in the LASSO models (left). Cross-validation for tuning parameter selection in the LASSO model (right). (**C**) Identification of overlapping genes from the Venn diagram. THEMIS2 expression between tumor and normal groups of the TCGA-THCA dataset (**D**), merged dataset (**E**), and single dataset (**F**). (**G**) GSEA for THEMIS2. (**H**) ROC curves and AUC statistics to evaluate the capacity of discrimination of tumor specimens from healthy controls showing excellent sensitivity and specificity. (**I**) Kaplan–Meier survival analysis for patients with thyroid cancer stratified by expression signature groups in the THCA dataset.

We performed LASSO regression analysis to identify hub genes linked with thyroid cancer diagnosis. The TCGA-THCA and merged datasets were used to construct the LASSO model and validate its role in clinical settings. Model 1 (merge GEO dataset) was optimized by ten-fold cross-validation at λ = 0.00001 and comprised 34 key genes (RBM6, ZCCHC12, HAVCR2, USP34, PAPOLG, FBLN2, GBP2, TNPO2, ARPC2, HEPH, NDC80, ASPDH, NCF2, ASB16, HCCS, CRISPLD2, LRRK2, NIFK-AS1, SAMD13, COA5, THEMIS2, APOO, GALNT7, PDGFRL, SAMSN1, C1QA, PELP1, CD2, RXRG, TMEM243, PSD3, UBE2C, NKTR, and HCK) as variables (Fig. 5A). Model 2 (THCA dataset) was optimized by ten-fold cross-validation at λ = 0.0000015 and comprised 13 key genes (ZCCHC12, LRP4, CPQ, TFF3, EPN1, KIF20A, THEMIS2, DCN, CTSA, MPZL2, MMRN2, BRD2, and STXBP5L) as variables (Fig. 5B). Venn diagram analysis identified two overlapping genes (THEMIS2 and ZCCHC12) (Fig. 5C) between the two LASSO models. The role of ZCCHC12 in thyroid cancer has been reported previously^13^. Therefore, THEMIS2 was subjected to further analysis. In the TCGA-THCA dataset, THEMIS2 was significantly highly expressed in the tumor group compared with the normal group (Fig. 5D). There was no significant difference in the THEMIS2 expression between the two groups of the merged dataset and the single dataset (Fig. 5E,F). We generated ROC curves and calculated the corresponding AUC values to evaluate the diagnostic performance of THEMIS2 expression. Finally, we defined THEMIS2 as a diagnostic biomarker of thyroid cancer because AUC exceeded 0.65 (AUC = 0.677; Fig. 5G). GSEA showed that THEMIS2 expression was mainly enriched in the JAK-STAT signaling pathway, T cell receptor signaling pathway, p53 signaling pathway, and B cell receptor signaling pathway (Fig. 5H). The TCGA-THCA dataset provided prognostic information, suggesting that patients with low THEMIS2 expression had a poor survival rate (Fig. 5I).

### THEMIS2 expression and localization

**Figure 6:**
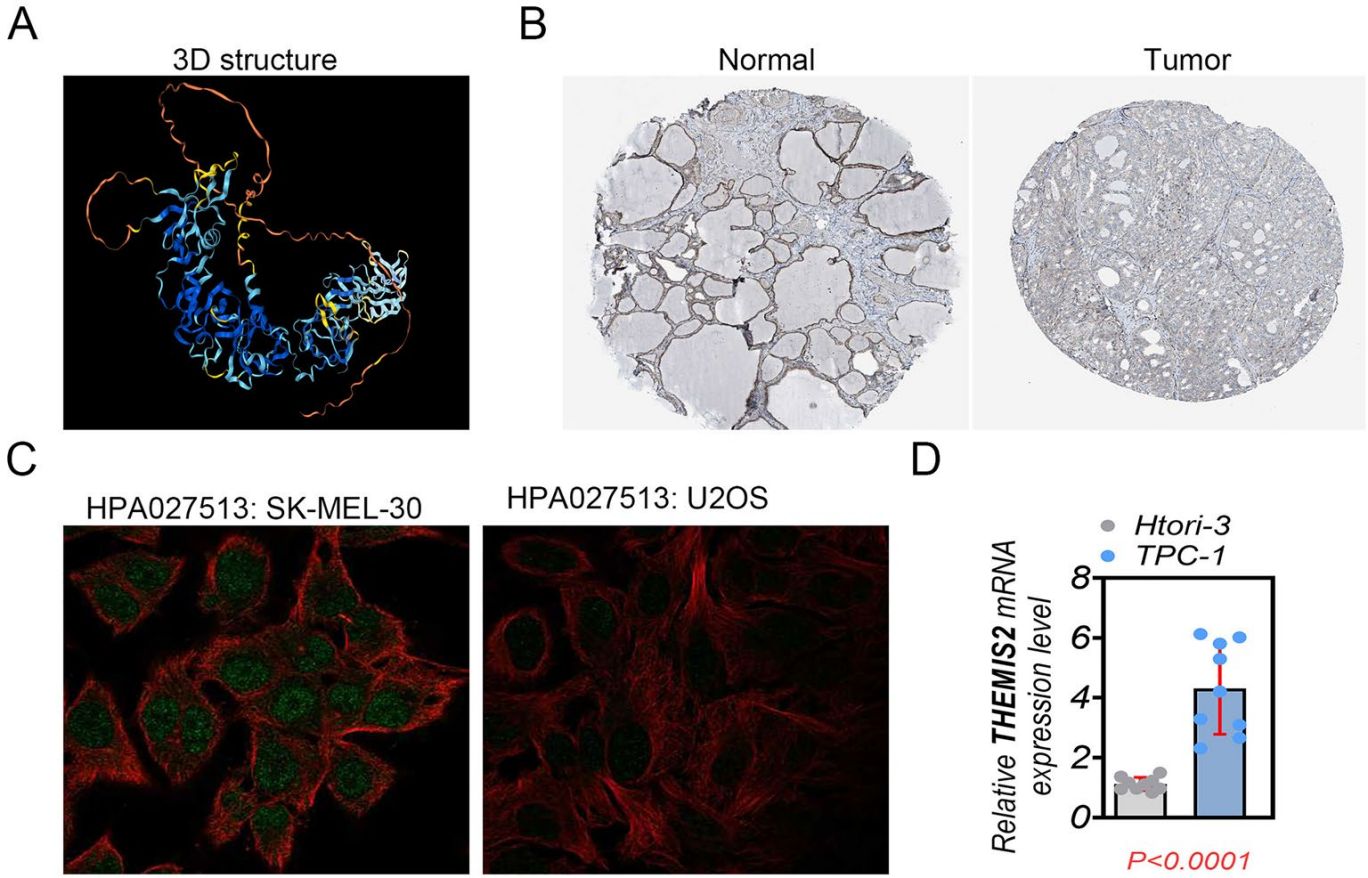
THEMIS2 expression and localization. (**A**) 3D structure of THEMIS2. (**B**) THEMIS2 expression in tumor and normal tissues from immunohistochemistry analysis from the HPA database. (**C**) Localization of THEMIS2 in different cells. (**D**) RT-PCR analysis of THEMIS2 expression in THCA cells. The data represent the mean ± SD of three independent experiments, and the level of significance was indicated by ***P < 0.0001.

**Figure 7:**
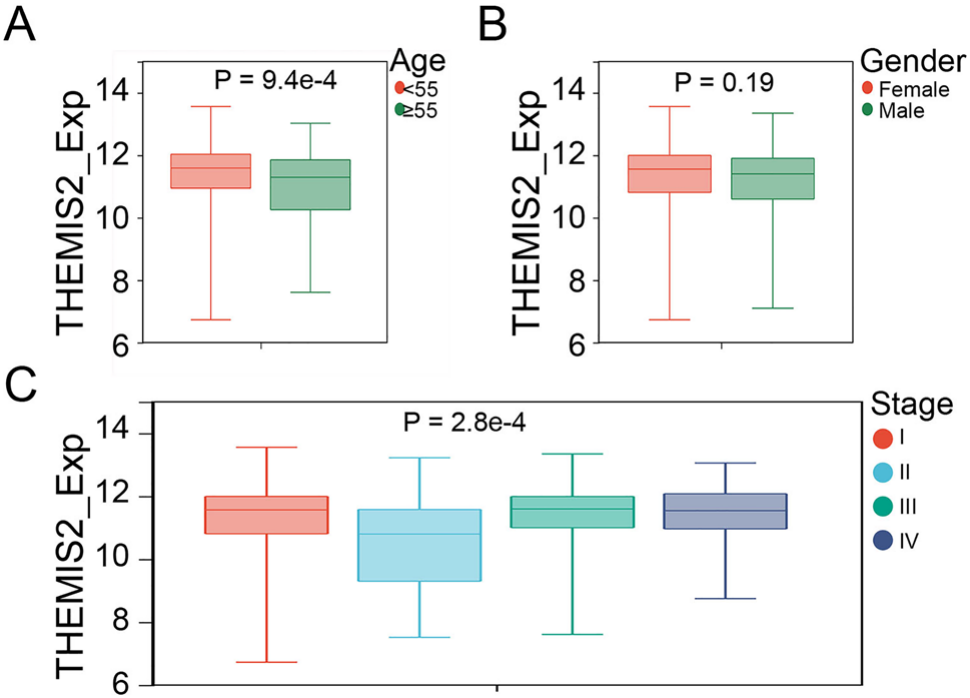
THEMIS2 expression according to age, sex, and stage. (**A**) THEMIS2 expression between < 50 and ≥ 55-year-old patients. (**B**) THEMIS2 expression between females and males. (**C**) THEMIS2 expression across stages I, II, III, and IV.

From the HPA data, a three-dimensional structure of THEMIS2 was obtained, as shown in Fig. 6A. THEMIS2 protein expression was positive in normal and tumor tissues (Fig. 6B). It was localized to the nucleoplasm (Fig. 6C). The results of RT-PCR results showed that the mRNA expression of THEMIS2 was higher in the THCA cell group (Fig. 6D). We explored its expression pattern based on the clinical characteristics of patients. The mRNA expression of THEMIS2 was lower in patients with thyroid cancer aged ≥ 55 years than in those aged < 55 years (Fig. 7A). THEMIS2 expression did not differ between males and females (Fig. 7B). Its levels of expression fluctuated dramatically across tumor stages (Fig. 7C).

### Correlation between THEMIS2 expression and immune infiltration

**Figure 8:**
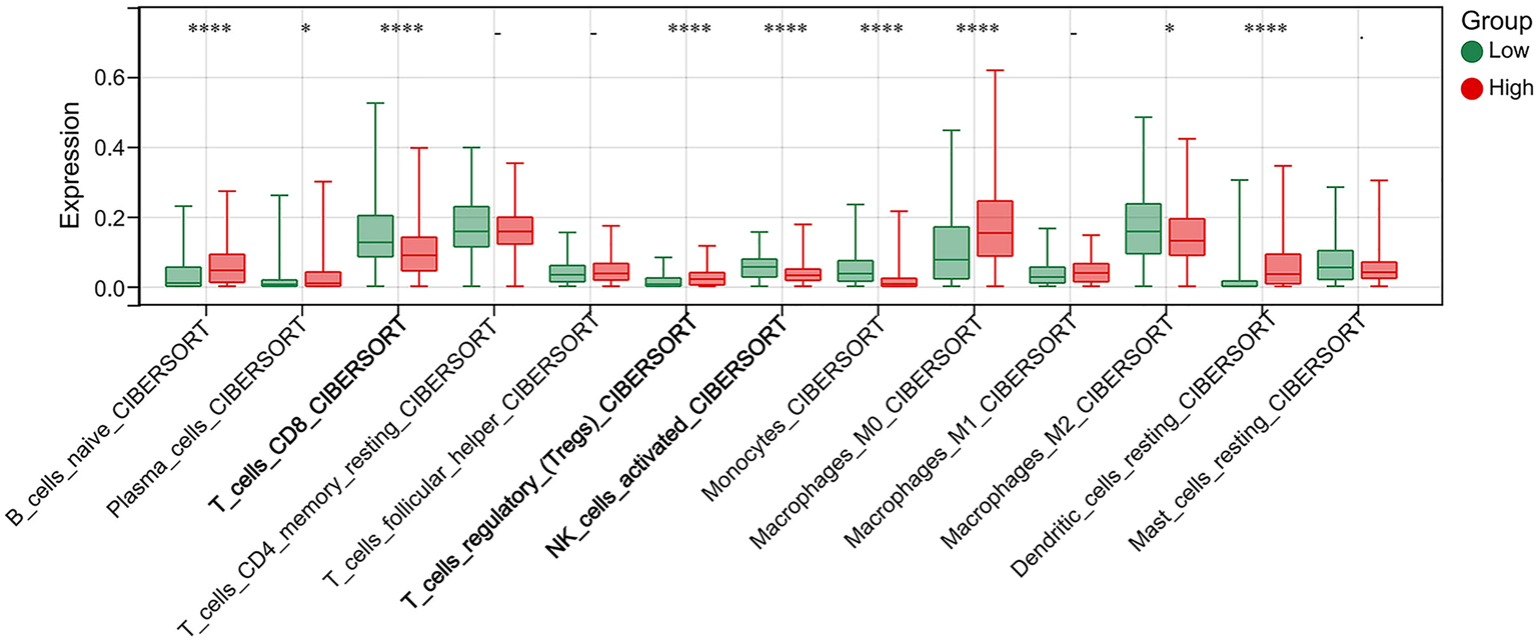
Correlation between THEMIS2 expression and immune infiltration. (**A**) Correlation between THEMIS2 expression and immune infiltrates from CIBERSORT.

Based on the enrichment analysis results, THEMIS2 was found to regulate immune system processes and T and B cell receptor signaling pathways. Consequently, we sought to examine the relationship between the expression of THEMIS2 and immune cell infiltration. Based on the best cutoff value of THEMIS2 expression in K–M survival analysis, 123 cases and 370 cases were assigned to low and high-expression groups, respectively. CIBERSORT analyses showed that multiple immune cell types were associated with high THEMIS2 expression (Fig. 8A). The high THEMIS2 expression group had lower T_cells_CD8 levels and activated NK cell levels and higher Tregs cell levels compared to the low THEMIS2 expression group.

### Correlation between THEMIS2 expression and immune checkpoints

**Figure 9:**
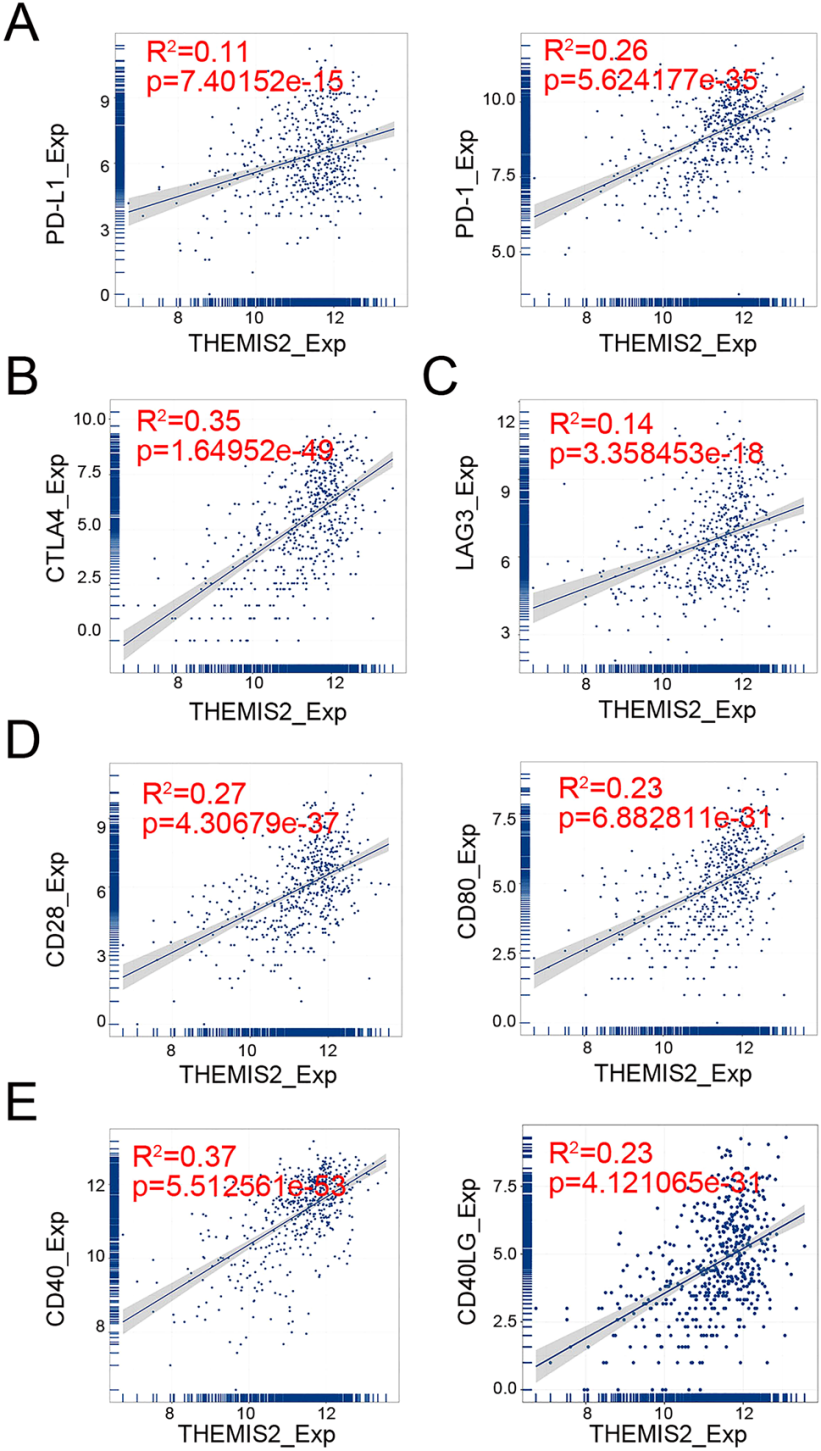
Correlation between THEMIS2 expression and immune checkpoints. (**A**) Correlation between THEMIS2 expression and PD-1/PD-L1 levels. (**B**) Correlation between THEMIS2 expression and CTLA4 levels. (**C**) Correlation between THEMIS2 expression and LAG3 levels. (**D**) Correlation between THEMIS2 expression and CD28/CD80 levels. (**E**) Correlation between THEMIS2 expression and CD40LG/CD40 levels.

THEMIS2 expression is closely related to immune cell function, which expresses a wide range of molecules called immune checkpoints that regulate the degree of immune activation. Over-expression or over-functioning of immune checkpoint molecules can inhibit immune function and promote tumor progression. PD-L1/PD-1, CTLA4, LAG3, CD28/CD80, and CD40/CD40LG are universal immune checkpoints^14,15^. THEMIS2 expression correlated positively with these immune checkpoints (Fig. 9). Thus, THEMIS2 exhibited immunosuppression.

### THEMIS2, as a prognostic factor in thyroid carcinoma, is affected by age and stage

We investigated THEMIS2’s prognostic significance in thyroid carcinoma. Table 1 shows the sample distribution. Univariate and multiple Cox regression analyses were performed to determine whether THEMIS2 expression was an independent predictor of thyroid cancer. Univariate Cox regression analysis demonstrated age, stage, and THEMIS2 expression as important factors of prognosis of patients with thyroid cancer (Table 2). However, multiple Cox regression analysis demonstrated that age was the only independent prognostic factor for patients with thyroid cancer (Table 3). THEMIS2 expression showed no significant independent role.

**Table 1:** Characteristics of clinical samples.

Characteristics	THEMIS2_Low_Exp (N = 123)	THEMIS2_High_Exp (N = 370)	Total (N = 493)	*P-*value	FDR
Age_group				7.20E−04	3.60E−03
< 55	66 (13.39%)	262 (53.14%)	328 (66.53%)		
≥ 55	57 (11.56%)	108 (21.91%)	165 (33.47%)		
Sex				0.23	0.25
Female	85 (17.24%)	278 (56.39%)	363 (73.63%)		
Male	38 (7.71%)	92 (18.66%)	130 (26.37%)		
Type				6.90E−16	4.80E−15
FTC	57 (11.56%)	44 (8.92%)	101 (20.49%)		
PTC	66 (13.39%)	326 (66.13%)	392 (79.51%)		
Stage				1.30E−03	3.90E−03
I	65 (13.24%)	210 (42.77%)	275 (56.01%)		
II	24 (4.89%)	27 (5.50%)	51 (10.39%)		
III	22 (4.48%)	89 (18.13%)	111 (22.61%)		
IV	11 (2.24%)	43 (8.76%)	54 (11.00%)		
T_stage				0.13	0.25
T1	39 (7.91%)	103 (20.89%)	142 (28.80%)		
T2	49 (9.94%)	114 (23.12%)	163 (33.06%)		
T3	31 (6.29%)	132 (26.77%)	163 (33.06%)		
T4	4 (0.81%)	19 (3.85%)	23 (4.67%)		
TX	0 (0.0e + 0%)	2 (0.41%)	2 (0.41%)		
N_stage				1.30E−08	7.70E−08
N0	76 (15.42%)	150 (30.43%)	226 (45.84%)		
N1	26 (5.27%)	191 (38.74%)	217 (44.02%)		
NX	21 (4.26%)	29 (5.88%)	50 (10.14%)		
M_stage				9.80E−04	3.90E−03
M0	51 (10.37%)	226 (45.93%)	277 (56.30%)		
M1	3 (0.61%)	6 (1.22%)	9 (1.83%)		
MX	68 (13.82%)	138 (28.05%)	206 (41.87%)		
Age (years)					
Mean ± SD	51.00 ± 16.25	46.22 ± 15.58	47.41 ± 15.87		
Median [min–max]	52.00 [18.00, 83.00]	45.00 [15.00, 89.00]	46.00 [15.00, 89.00]		
THEMIS2_Exp					
Mean ± SD	9.71 ± 0.86	11.76 ± 0.53	11.25 ± 1.09		
Median [min–max]	9.93 [6.73, 10.76]	11.75 [10.77, 13.56]	11.53 [6.73, 13.56]

**Table 2:** Univariate COX regression analysis.

Id	Coef	HR	95% Lower	95% Higher	P-value
THEMIS2_Exp_group	− 1.023	0.359	0.133	0.97	0.043
age	2.873	17.698	3.029	103.413	0.001
Gender	− 0.347	0.707	0.425	1.175	0.181
Stage	1.960	7.101	2.285	22.070	0.001

**Table 3:** Multivariate COX regression analysis.

Id	Coef	HR	95% Lower	95% Higher	P-value
THEMIS2_Exp_group	− 0.818	0.441	0.159	1.225	0.116
Age	0.149	1.160	1.097	1.226	0.000
Stage	0.315	1.370	0.794	2.365	0.629

We determined the association between THEMIS2 expression and age and stage. Age and stage showed multiplicative interactions with THEMIS2 expression (Table 4). Thus, prognosis based on THEMIS2 expression was significantly affected by age and stage.

**Table 4:** Analysis of multiplicative interaction between age and stage and THEMIS2 expression.

Interaction terms	TNFRSF11B interaction				
	Coef	HR	95% Lower	95% Higher	P-value
Age*THEMIS2	0.096	1.101	1.062	1.141	0.000
Stage*THEMIS2	1.397	4.042	1.761	9.274	0.001

**Figure 10:**
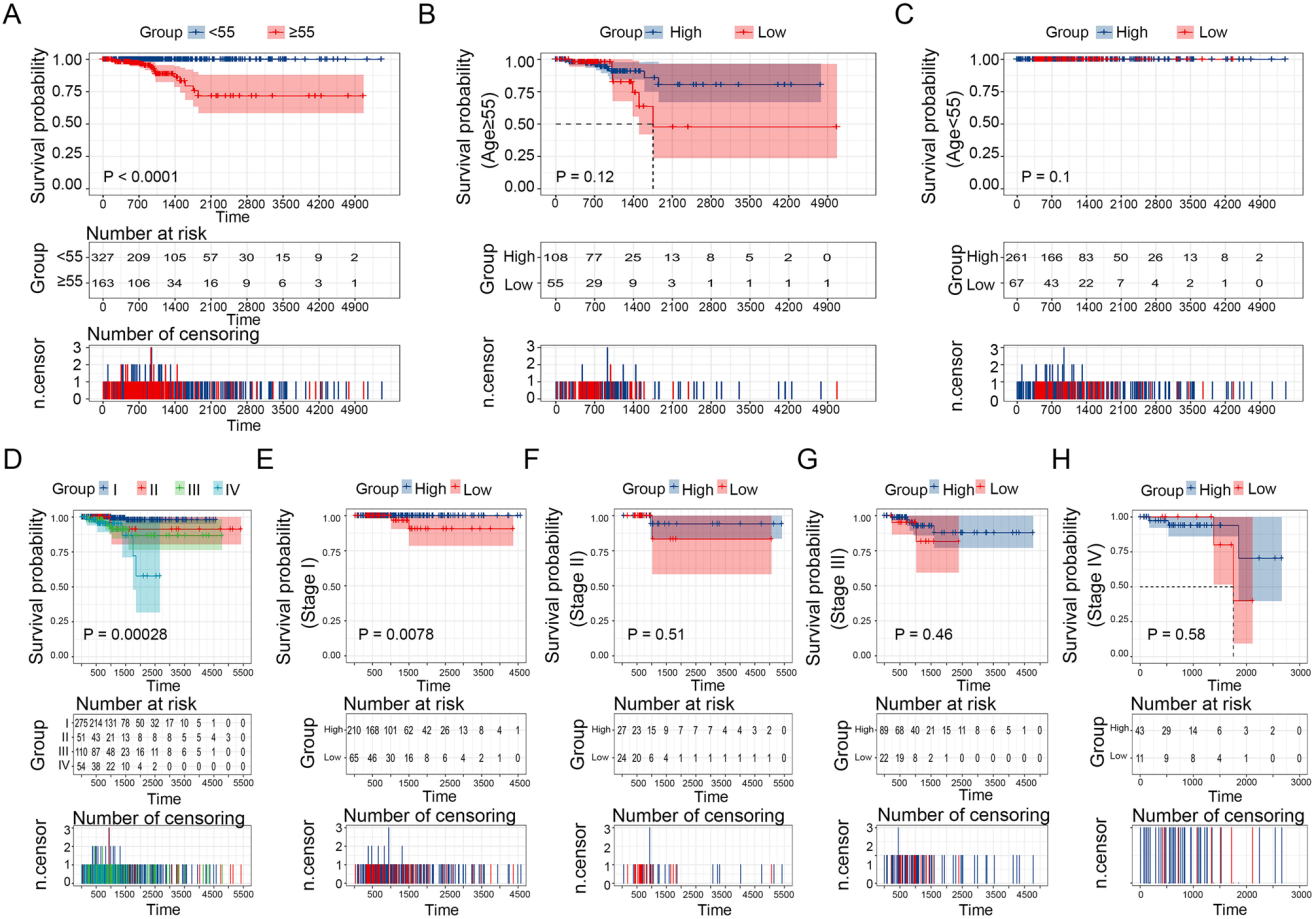
Survival analysis in stratified groups based on THEMIS2 expression. (**A**) The difference in the survival of patients with thyroid cancer across different age groups in the THCA dataset. Survival analysis for patients with thyroid cancer stratified by THEMIS2 expression in the ≥ 55 (**B**) years and < 55 years (**C**) groups. (**D**) The difference in the survival of patients with thyroid cancer across different stages. Kaplan–Meier survival analysis for patients with thyroid cancer stratified by THEMIS2 expression at stage I (**E**), stage II (**F**), stage III (**G**), and stage IV (**H**).

We explored the prognostic role of age and stage and found that patients over the age of 55 years and belonging to stage IV had a dismal prognosis from survival analyses (Fig. 10A,D). We examined the prognostic effect of THEMIS2 expression on patients among subgroups grouped by age and stage. The survival status of patients between high- and low-THEMIS2 expression groups did not differ between ≥ 55 or < 55 years subgroups (Fig. 10B,C). Among the four stages, only the survival status of patients in stage I differed considerably between the high- and low-THEMIS2 expression groups (Fig. 10E). There was no significant variation in the survival status of patients in stages II, III, and IV (Fig. 10F–H).

### Nomogram model based on THEMIS2 expression for thyroid cancer

**Figure 11:**
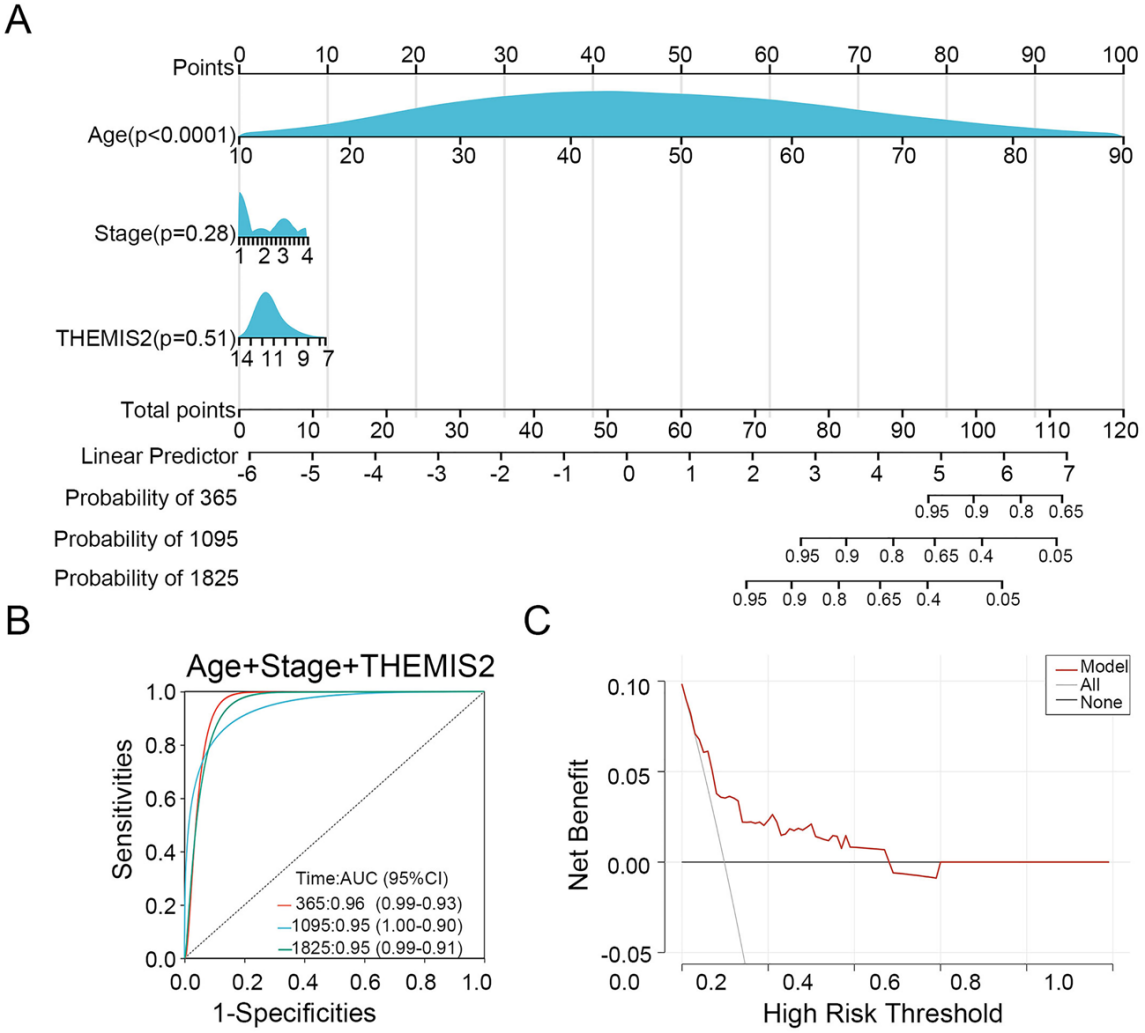
A comprehensive nomogram model based on THEMIS2 expression to evaluate the prognosis of patients with thyroid cancer. (**A**) The nomogram model was constructed based on age, stage, and THEMIS2 expression. (**B**) ROC curves for the nomogram. (**C**) DCA for the nomogram.

Based on the three prognosis-related variables, including age, clinical stage, and THEMIS2 expression, a nomogram model was established for the prognostic assessment of thyroid cancer (Fig. 11A). The ROC curves for TCGA-THCA dataset were plotted, and the AUC of the nomogram model was 0.96, 0.95, and 0.95 for 1-, 3- and 5-year survival (Fig. 11B). DCA showed positive net benefits for the nomogram model (Fig. 11C).

## Discussion

In the study, 223 overlapping genes showed significant associations with both age and type of thyroid cancer. Based on GO annotation terms, these genes primarily regulate immune system processes, activate immune responses, modulate extracellular matrix dynamics, govern mitosis, and control cell cycle progression. Fundamental physiological characteristics of cancer cells are uncontrolled and unrestrained proliferation. The extracellular matrix is crucial for cancer cell proliferation, invasion, and metastasis^16,17^. KEGG results showed that the genes were mainly related to the JAK-STAT and p53 signaling pathways. Mutations in or inactivation of the p53 gene can promote carcinogenesis^18^. Therefore, patients with thyroid cancer patients are recommended to undergo p53 gene testing to assess their condition accurately^19^. The JAK/STAT signaling pathway is a pivotal communication hub in cellular activity and crucial in cancer^20^. By interacting with a diverse array of cytokines, the JAK/STAT signaling system affects immune cell differentiation and development by functioning as an immunomodulator. Extensive evidence has demonstrated that the activation of the JAK/STAT signaling promotes the onset and progression of various diseases, including thyroid cancer^20,21^.

The 223 overlapping genes were subjected to LASSO analysis, leading to the discovery of two noteworthy genes, namely THEMIS2 and ZCCHC12. Previous studies have extensively reported the involvement of ZCCHC12 in thyroid carcinoma^13,22^, prompting our study’s focus on THEMIS2. GSEA revealed that THEMIS2 is intricately linked to both p53 signaling and the JAK-STAT signaling pathway. THEMIS2 is pivotal in rheumatoid arthritis^23^. Collectively, our findings suggest that THEMIS2 is crucial in immunity and the regulation of the p53 and JAK-STAT signaling pathways.

THEMIS2, also known as ICB-1 or C1orf38, exhibits predominant expression in B cells and macrophages. It governs the Toll-like receptor response, specifically in macrophages^24^, and is a distinct biomarker of chronic lymphocytic leukemia^25^. GSEA revealed a significant association of THEMIS2 with the B cell receptor signaling pathway, T cell receptor signaling pathway, and Toll-like receptor signaling pathway. Toll-like receptors (TLRs) modulate the tumor microenvironment (TME) by activating innate immune responses and facilitating adaptive immune response activation^26^. TME refers to the internal milieu wherein tumor cells thrive and comprise non-epithelial cells and extracellular matrix within the tumor tissue. Non-epithelial cells predominantly include immune cell infiltrates, fibroblasts, and vascular endothelial cells. T cells, B cells, NK cells, and macrophages are among the immune cell populations that infiltrate tumors^7,8^. Immune infiltration affects the clinical outcome of patients. THEMIS2 is overexpressed in thyroid cancer tumor samples and demonstrates remarkable discriminatory ability between cancerous and healthy controls, as indicated by the findings of our bioinformatics research. Patients with diminished THEMIS2 expression exhibited poorer survival outcomes, suggesting that THEMIS2 expression may serve as a diagnostic and prognostic marker for thyroid cancer.

Reduced numbers of CD8+ T cells and NK cells, along with elevated levels of Treg cells, serve as indicators for tumor recurrence and metastasis^27^. In the THEMIS2 high expression group, there was a decrease in the levels of CD8+ T cells, NK cells, and Treg cells, which strongly suggests an adverse effect of THEMIS2 on the immune system. The immune system is crucial for maintaining human health and equilibrium by recognizing pathogens and resisting tumor invasion through an immune surveillance system and escape mechanism. Immune checkpoints regulate the immune system and are crucial for maintaining autoimmune tolerance and regulating the duration and extent of immune responses in peripheral tissues^28^. Antibody release by tumor cells that correspondingly inhibit T cell activation enables tumor cells to evade immune surveillance. T cell activation necessitates the presence of costimulatory receptor/ligand pairs, such as CD28/CD80 and CD40L/CD40, along with immunosuppressive receptor/ligand pairs, including CTLA-4/CD80 and PD-1/PD-L1 13,14. LAG-3 restrains T cell activity and participates in their negative regulation^14,15^. Consequently, targeting these aforementioned immune checkpoints can abrogate T cell inhibition and augment overall immune function in the body. The results revealed a significant positive correlation between THEMIS2 expression in thyroid cancer and the expression of these immune checkpoints. Patients with lower THEMIS2 expression exhibited reduced immunosuppression, indicating a more favorable response to treatment.

We investigated the prognostic value of THEMIS2 expression in thyroid cancer. While THEMIS2, age, and stage showed significant associations with patient survival in the univariate COX analysis, multivariate COX analysis revealed that only age remained an independent prognostic factor for thyroid cancer. Interaction analysis suggested that the prognostic effect of THEMIS2 in thyroid carcinoma is primarily affected by age and stage. This could be attributed to the fact that THEMIS2 is derived from module genes correlated strongly with age and different types of thyroid cancer. A higher prevalence of thyroid cancer has been reported among individuals aged 45 years or older^29^. Furthermore, combining THEMIS2 expression with age and stage can significantly improve the predictive performance of disease prognosis.

THEMIS2 exhibits strong correlations with age and tumor type and is significantly upregulated in thyroid cancer tumors. Patients with low THEMIS2 expression showed significantly lower survival rates than those with high THEMIS2 expression. THEMIS2 is closely associated with the p53 signaling pathway, the JAK-STAT signaling pathway, and the immune-related signaling pathway. It functions as an inhibitor of T-cell activation and is positively correlated with the expression of immune checkpoints. Age is a primary prognostic factor for THEMIS2. Our findings provide a precise nomogram for predicting prognosis in patients with thyroid cancer and hold potential clinical applicability. THEMIS2 may serve as a novel marker for immune infiltration in thyroid cancer by activating the p53 and JAK-STAT signaling pathways, inhibiting T-cell activation, and facilitating the occurrence and progression of thyroid cancer.

### Supplementary Information

Supplementary Information.

Supplementary Table S1.

## Data Availability

All data generated or analysed during this study are included in this supplementary information files.

## References

[1] Khan, Y. S. & Farhana, A. in *StatPearls* (2023).

[2] Laha D, Nilubol N, Boufraqech M (2020). New therapies for advanced thyroid cancer. Front. Endocrinol. (Lausanne).

[3] Wang J, Yu F, Shang Y, Ping Z, Liu L (2020). Thyroid cancer: Incidence and mortality trends in China, 2005–2015. Endocrine.

[4] Vuong HG, Le MK, Hassell L, Kondo T, Kakudo K (2022). The differences in distant metastatic patterns and their corresponding survival between thyroid cancer subtypes. Head Neck.

[5] Chan S (2020). Systematic review of recurrence rate after hemithyroidectomy for low-risk well-differentiated thyroid cancer. Eur. Thyroid J..

[6] Xiao Y, Yu D (2021). Tumor microenvironment as a therapeutic target in cancer. Pharmacol. Ther..

[7] Lv B (2022). Immunotherapy: Reshape the tumor immune microenvironment. Front. Immunol..

[8] Lin Y (2022). Geospatial immune heterogeneity reflects the diverse tumor-immune interactions in intrahepatic cholangiocarcinoma. Cancer Discov..

[9] Zhang S (2019). DNA methylation exploration for ARDS: A multi-omics and multi-microarray interrelated analysis. J. Transl. Med..

[10] Langfelder P, Horvath S (2008). WGCNA: An R package for weighted correlation network analysis. BMC Bioinform..

[11] Yu G, Wang LG, Han Y, He QY (2012). clusterProfiler: An R package for comparing biological themes among gene clusters. OMICS.

[12] Pei D (2022). A novel prognostic signature associated with the tumor microenvironment in kidney renal clear cell carcinoma. Front. Oncol..

[13] Wang O (2017). ZCCHC12, a novel oncogene in papillary thyroid cancer. J. Cancer Res. Clin. Oncol..

[14] Saleh R, Toor SM, Khalaf S, Elkord E (2019). Breast cancer cells and PD-1/PD-L1 blockade upregulate the expression of PD-1, CTLA-4, TIM-3 and LAG-3 immune checkpoints in CD4(+) T cells. Vaccines (Basel).

[15] Wan X (2022). Tolerogenic dendritic cells alleviate collagen-induced arthritis by forming microchimerism and affecting the expression of immune checkpoint molecules. Eur. J. Immunol..

[16] Atat OE (2022). 3D modeling in cancer studies. Hum. Cell.

[17] Huang J (2021). Extracellular matrix and its therapeutic potential for cancer treatment. Signal Transduct. Target. Ther..

[18] Hernandez Borrero LJ, El-Deiry WS (2021). Tumor suppressor p53: Biology, signaling pathways, and therapeutic targeting. Biochim. Biophys. Acta Rev. Cancer.

[19] Manzella L (2017). New insights in thyroid cancer and p53 family proteins. Int. J. Mol. Sci..

[20] Li H (2022). Nav1.6 promotes the progression of human follicular thyroid carcinoma cells via JAK-STAT signaling pathway. Pathol. Res. Pract..

[21] Ptacek J (2021). Diminished cytokine-induced Jak/STAT signaling is associated with rheumatoid arthritis and disease activity. PLoS One.

[22] Cui Y, Dong YY (2022). ZCCHC12 promotes the progression of osteosarcoma via PI3K/AKT pathway. Aging (Albany NY).

[23] Xing X, Xia Q, Gong B, Shen Z, Zhang Y (2022). Identification of tissue-specific expressed hub genes and potential drugs in rheumatoid arthritis using bioinformatics analysis. Front. Genet..

[24] Deobagkar-Lele M, Anzilotti C, Cornall RJ (2017). Themis2: Setting the threshold for B-cell selection. Cell. Mol. Immunol..

[25] Hengeveld PJ (2023). High-throughput proteomics identifies THEMIS2 as independent biomarker of treatment-free survival in untreated CLL. Hemasphere.

[26] McCall KD, Muccioli M, Benencia F (2020). Toll-like receptors signaling in the tumor microenvironment. Adv. Exp. Med. Biol..

[27] Farhood B, Najafi M, Mortezaee K (2019). CD8(+) cytotoxic T lymphocytes in cancer immunotherapy: A review. J. Cell. Physiol..

[28] Omar HA (2019). Immunomodulatory MicroRNAs in cancer: Targeting immune checkpoints and the tumor microenvironment. FEBS J..

[29] Konstantinidis A, Tracy E, Sosa JA, Roman SA (2017). Risk prediction in children and adults less than 45 years old with papillary thyroid cancer. Expert Rev. Endocrinol. Metab.

