# Supplementary material for: Comprehensive bioinformatics analysis unveils THEMIS2 as a carcinogenic indicator related to immune infiltration and prognosis of thyroid cancer
Source: Sci Rep. 2024 Apr 8;14:8156. doi: 10.1038/s41598-024-58943-6 (PMC11001958; doi:10.1038/s41598-024-58943-6)
Supplement: Supplementary file 1 — Supplementary Information. [file 41598_2024_58943_MOESM1_ESM.zip › Raw data/Raw data/5. THEMIS2_analysis/Age小于55Survival_Analysis.pdf]

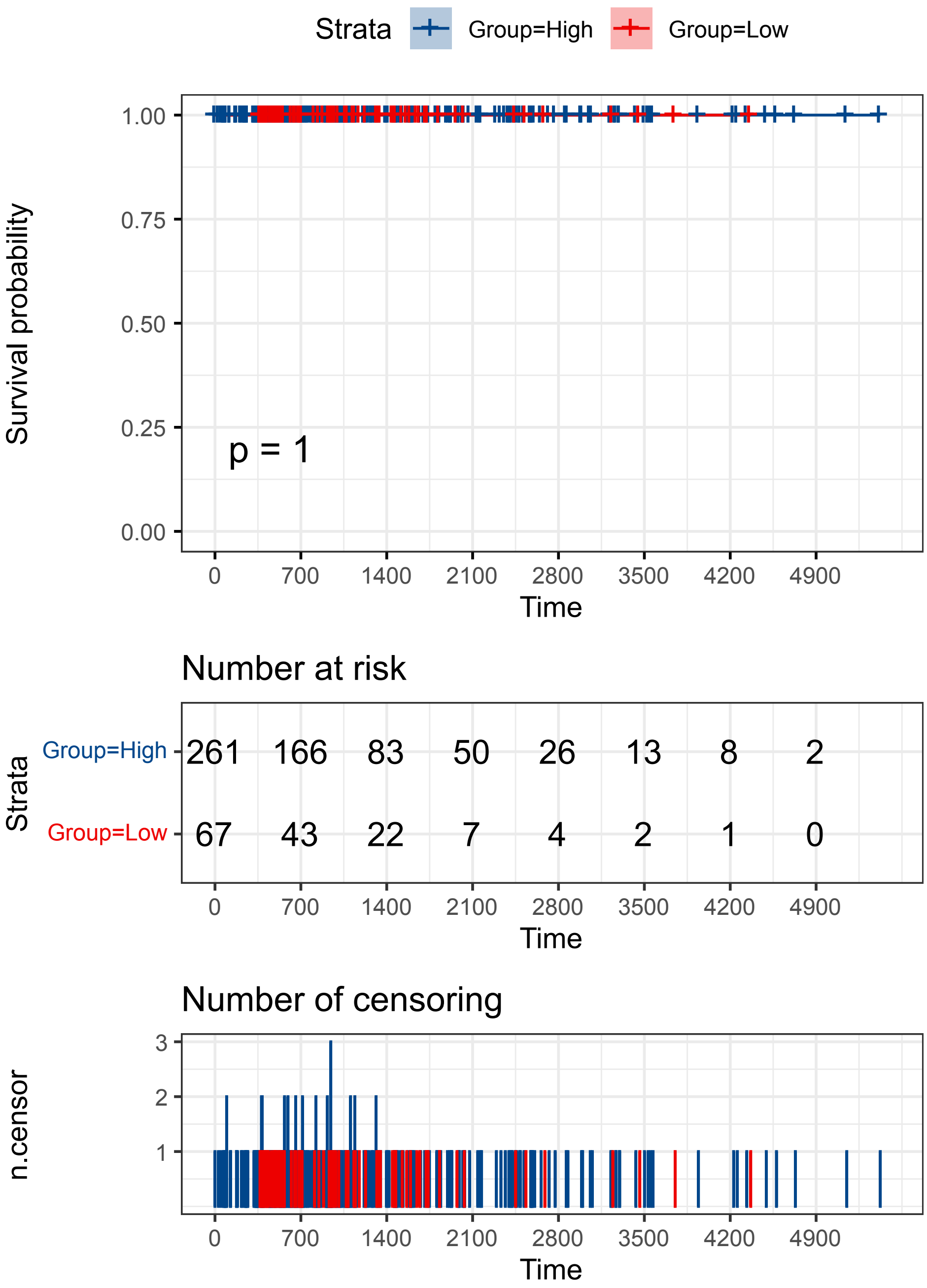

Strata
Group=High
Group=Low
Survival probability
1.00
0.75
0.50
0.25
0.00
p = 1
0
700
1400
2100
2800
3500
4200
4900
Time
Number at risk
Strata
Group=High
Group=Low
261
166
83
50
26
13
8
2
67
43
22
7
4
2
1
0
Time
Number of censoring
n.censor
3
2
1
Time
