# Supplementary material for: Comprehensive bioinformatics analysis unveils THEMIS2 as a carcinogenic indicator related to immune infiltration and prognosis of thyroid cancer
Source: Sci Rep. 2024 Apr 8;14:8156. doi: 10.1038/s41598-024-58943-6 (PMC11001958; doi:10.1038/s41598-024-58943-6)
Supplement: Supplementary file 1 — Supplementary Information. [file 41598_2024_58943_MOESM1_ESM.zip › Raw data/Raw data/5. THEMIS2_analysis/GSEA.pdf]

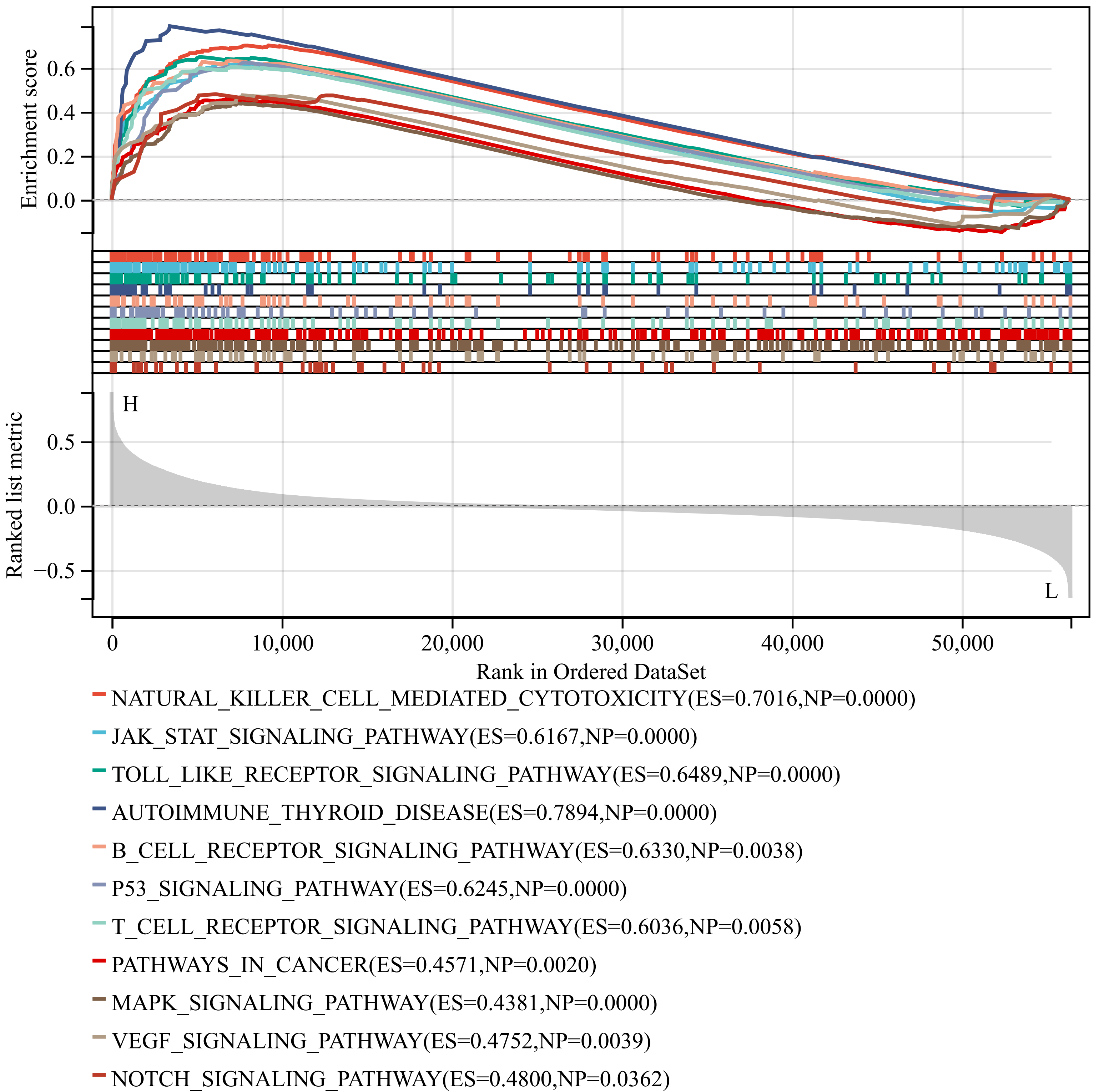
Enrichment score
0.6
0.4
0.2
0.0
Ranked list metric
0.5
0.0
−0.5
H
L
0
10,000
20,000
30,000
40,000
50,000
Rank in Ordered DataSet
NATURAL_KILLER_CELL_MEDIATED_CYTOTOXICITY(ES=0.7016,NP=0.0000)
JAK_STAT_SIGNALING_PATHWAY(ES=0.6167,NP=0.0000)
TOLL_LIKE_RECEPTOR_SIGNALING_PATHWAY(ES=0.6489,NP=0.0000)
AUTOIMMUNE_THYROID_DISEASE(ES=0.7894,NP=0.0000)
B_CELL_RECEPTOR_SIGNALING_PATHWAY(ES=0.6330,NP=0.0038)
P53_SIGNALING_PATHWAY(ES=0.6245,NP=0.0000)
T_CELL_RECEPTOR_SIGNALING_PATHWAY(ES=0.6036,NP=0.0058)
PATHWAYS_IN_CANCER(ES=0.4571,NP=0.0020)
MAPK_SIGNALING_PATHWAY(ES=0.4381,NP=0.0000)
VEGF_SIGNALING_PATHWAY(ES=0.4752,NP=0.0039)
NOTCH_SIGNALING_PATHWAY(ES=0.4800,NP=0.0362)
