# Supplementary material for: Comprehensive bioinformatics analysis unveils THEMIS2 as a carcinogenic indicator related to immune infiltration and prognosis of thyroid cancer
Source: Sci Rep. 2024 Apr 8;14:8156. doi: 10.1038/s41598-024-58943-6 (PMC11001958; doi:10.1038/s41598-024-58943-6)
Supplement: Supplementary file 1 — Supplementary Information. [file 41598_2024_58943_MOESM1_ESM.zip › Raw data/Raw data/5. THEMIS2_analysis/stageI+II_Survival_Analysis.pdf]

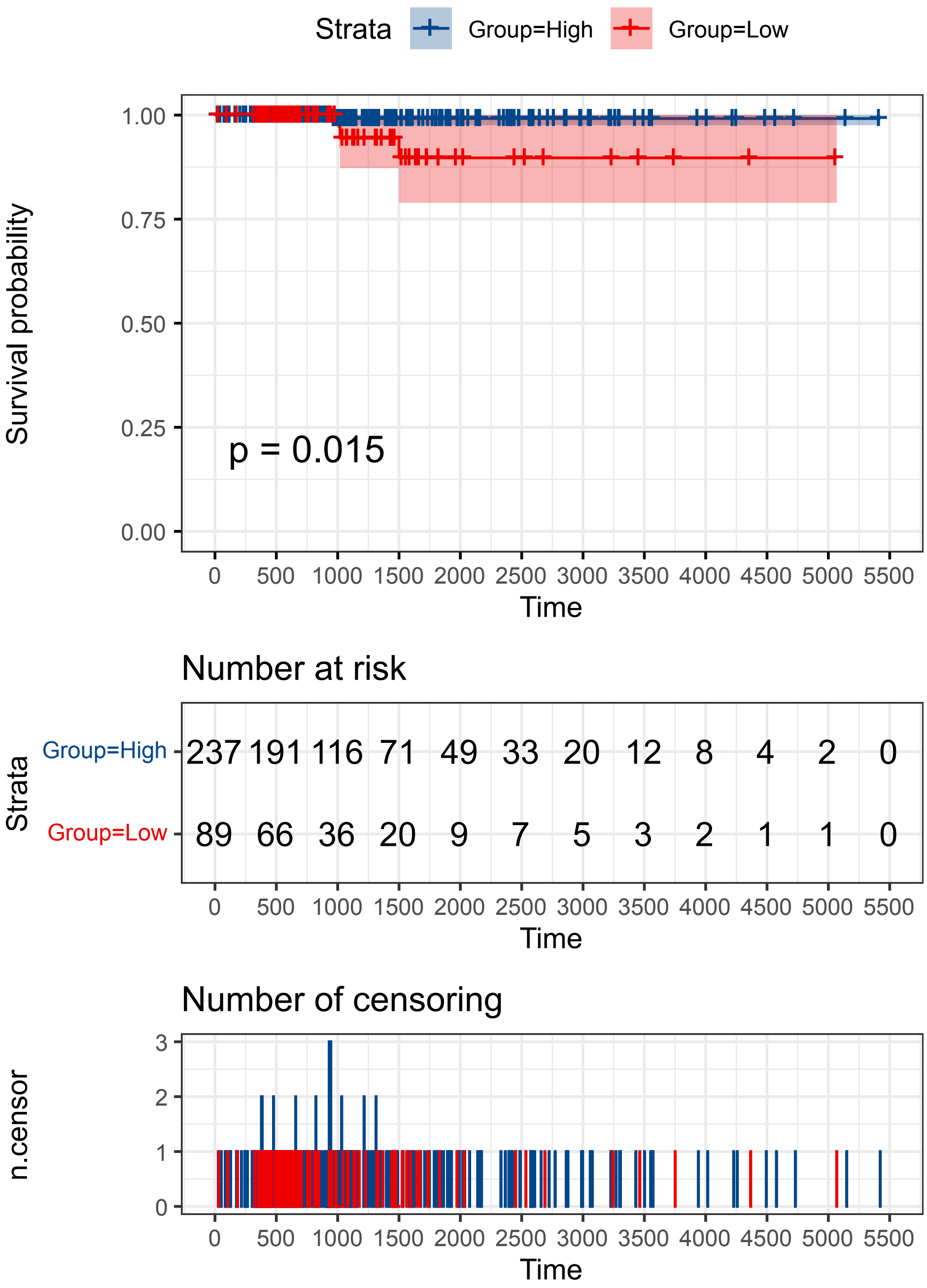
Strata
Group=High
Group=Low
Survival probability
1.00
0.75
0.50
0.25
0.00
p = 0.015
0 500 1000 1500 2000 2500 3000 3500 4000 4500 5000 5500
Time
Number at risk
Strata
Group=High 237 191 116 71 49 33 20 12 8 4 2 0
Group=Low 89 66 36 20 9 7 5 3 2 1 1 0
0 500 1000 1500 2000 2500 3000 3500 4000 4500 5000 5500
Time
Number of censoring
n.censor
3
2
1
0
0 500 1000 1500 2000 2500 3000 3500 4000 4500 5000 5500
Time
