# Supplementary material for: Comprehensive bioinformatics analysis unveils THEMIS2 as a carcinogenic indicator related to immune infiltration and prognosis of thyroid cancer
Source: Sci Rep. 2024 Apr 8;14:8156. doi: 10.1038/s41598-024-58943-6 (PMC11001958; doi:10.1038/s41598-024-58943-6)

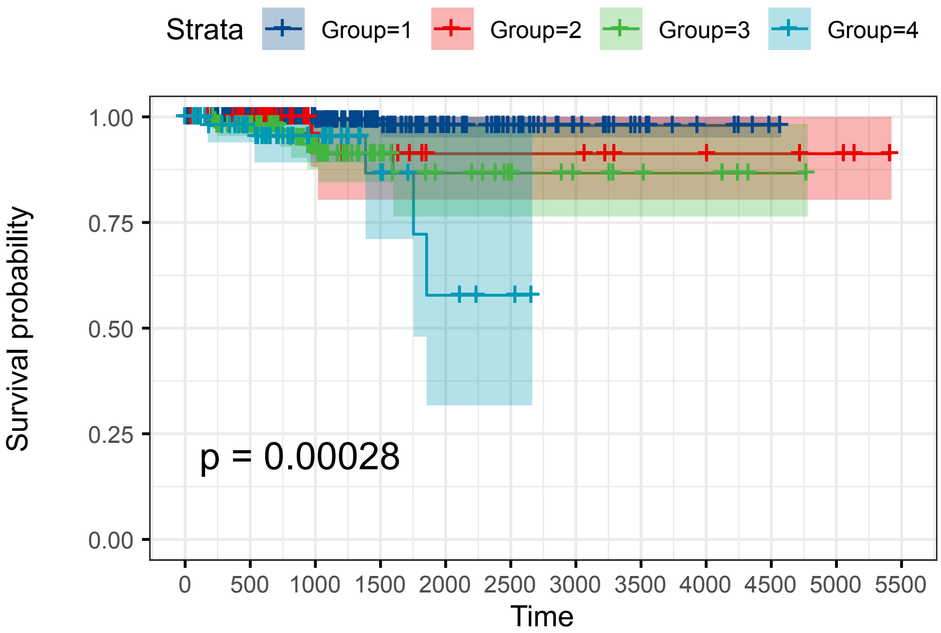

Number at risk

Strata	0	500	1000	1500	2000	2500	3000	3500	4000	4500	5000	5500
Group=1	275	214	131	78	50	32	17	10	5	1	0	0
Group=2	51	43	21	13	8	8	8	5	5	4	3	0
Group=3	110	87	48	23	16	11	8	6	5	1	0	0
Group=4	54	38	22	10	4	2	0	0	0	0	0	0

Time

Number of censoring

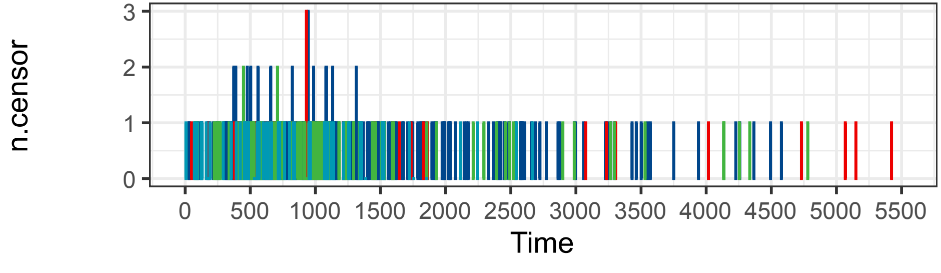

Supplement: Supplementary file 1 — Supplementary Information. [file 41598_2024_58943_MOESM1_ESM.zip › Raw data/Raw data/5. THEMIS2_analysis/Stage_I_II_III_IV_Survival_Analysis.pdf]
