# Supplementary figures and images for: Comprehensive bioinformatics analysis unveils THEMIS2 as a carcinogenic indicator related to immune infiltration and prognosis of thyroid cancer
Source: Sci Rep. 2024 Apr 8;14:8156. doi: 10.1038/s41598-024-58943-6 (PMC11001958; doi:10.1038/s41598-024-58943-6)

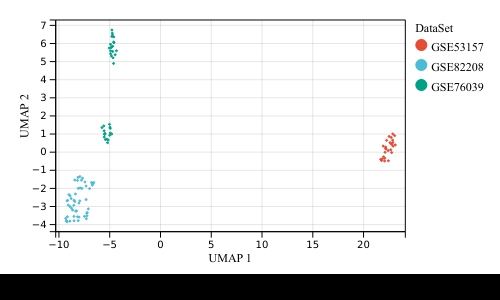

Supplement: Supplementary file 1 — Supplementary Information. [file 41598_2024_58943_MOESM1_ESM.zip › Raw data/Raw data/1.GEO&TCGA-THCA/71e2bea603d4096bd96cd04cb6b1f8de.jpeg]

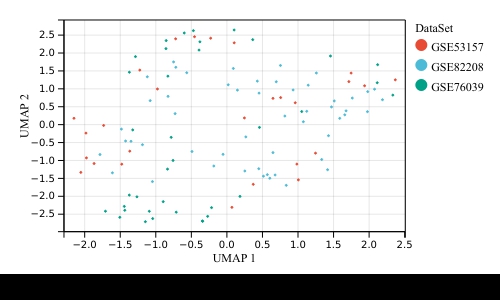

Supplement: Supplementary file 1 — Supplementary Information. [file 41598_2024_58943_MOESM1_ESM.zip › Raw data/Raw data/1.GEO&TCGA-THCA/7b3b9fce4942448b8607ada82a57da3f.jpeg]

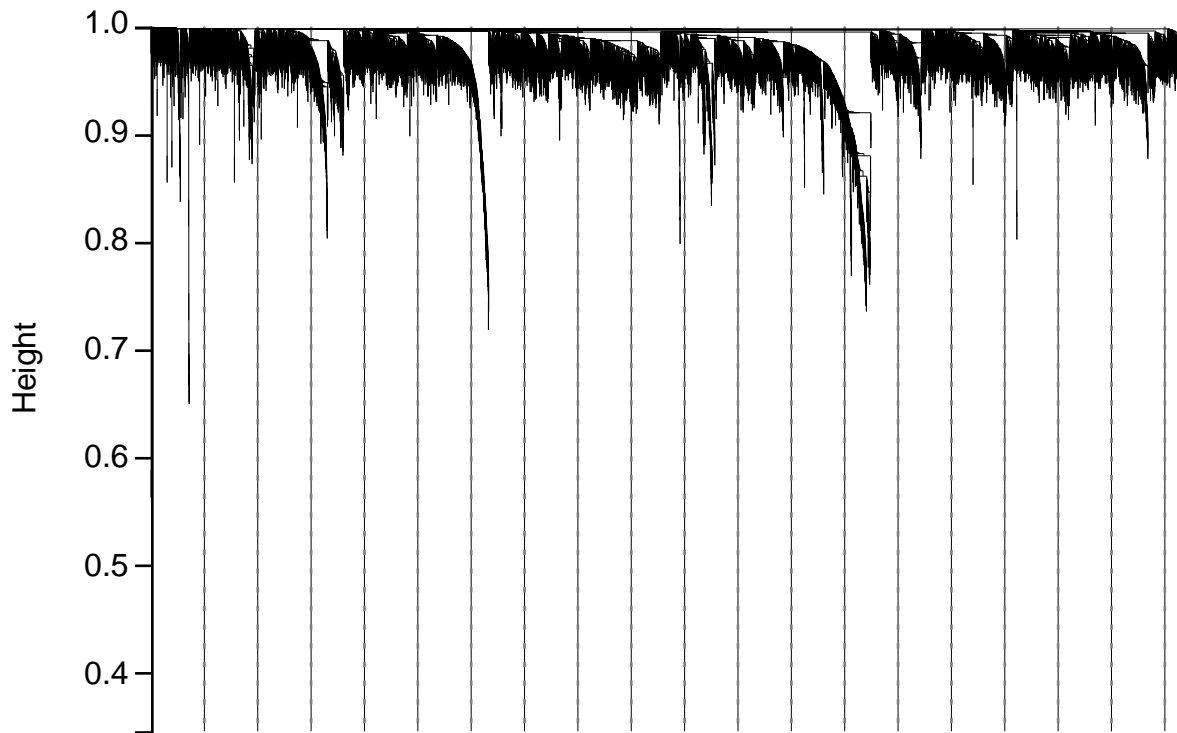

DynamicTreeCut

MergeDynamic

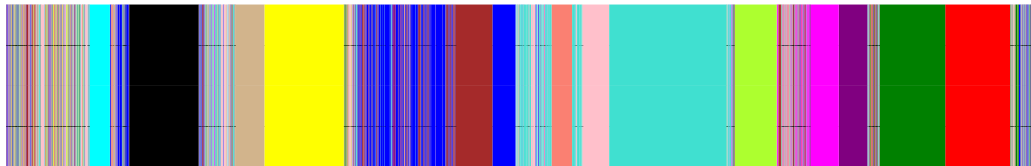

Supplement: Supplementary file 1 — Supplementary Information. [file 41598_2024_58943_MOESM1_ESM.zip › Raw data/Raw data/2.WGCNA/基因聚类.pdf]

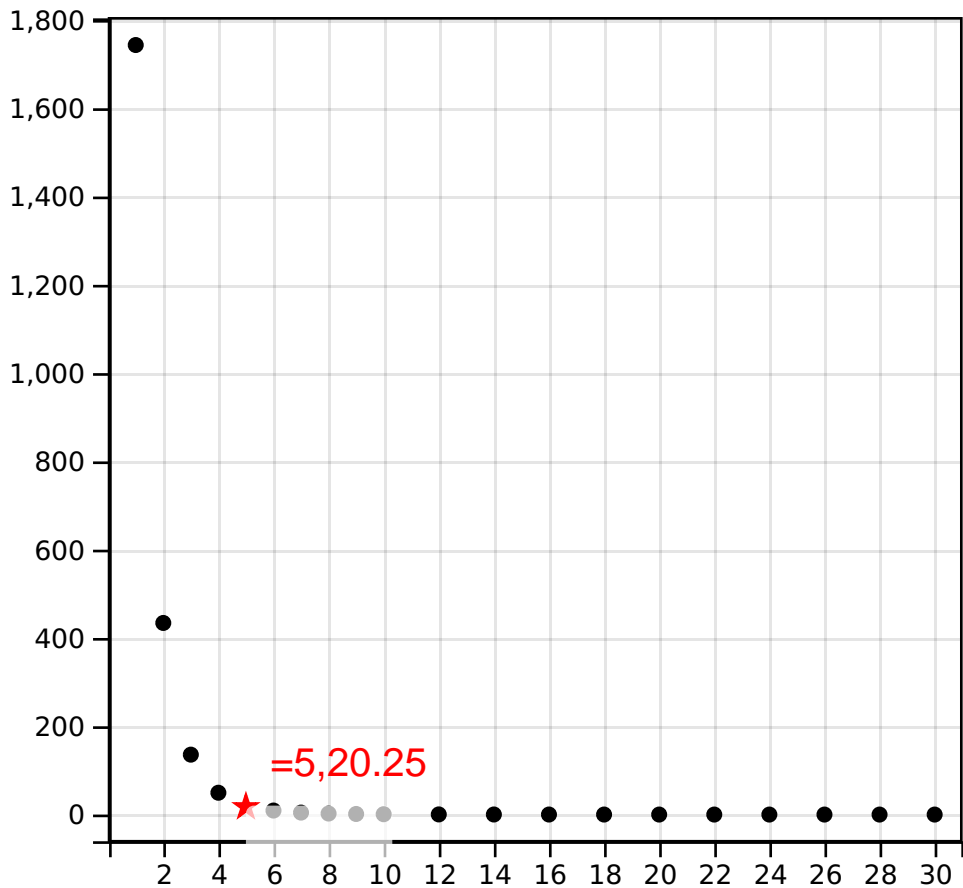

Supplement: Supplementary file 1 — Supplementary Information. [file 41598_2024_58943_MOESM1_ESM.zip › Raw data/Raw data/2.WGCNA/平均连通性.pdf]

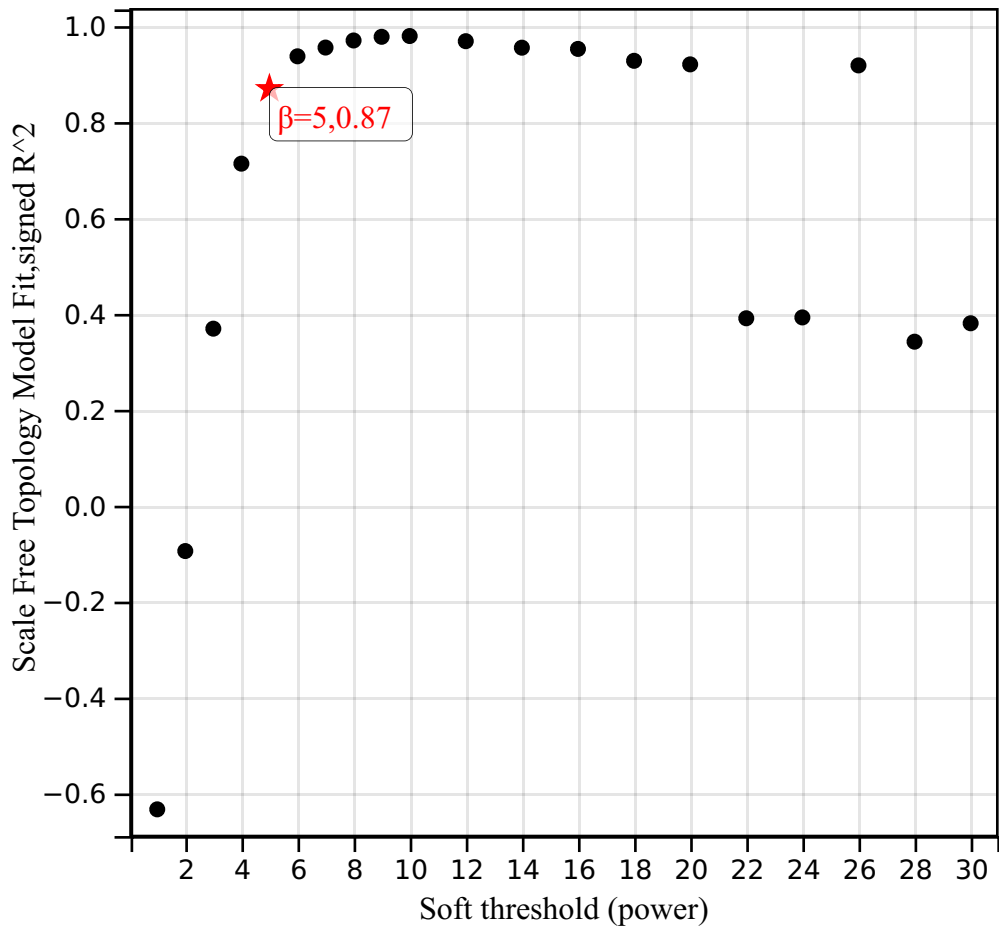

Supplement: Supplementary file 1 — Supplementary Information. [file 41598_2024_58943_MOESM1_ESM.zip › Raw data/Raw data/2.WGCNA/标度独立性.pdf]

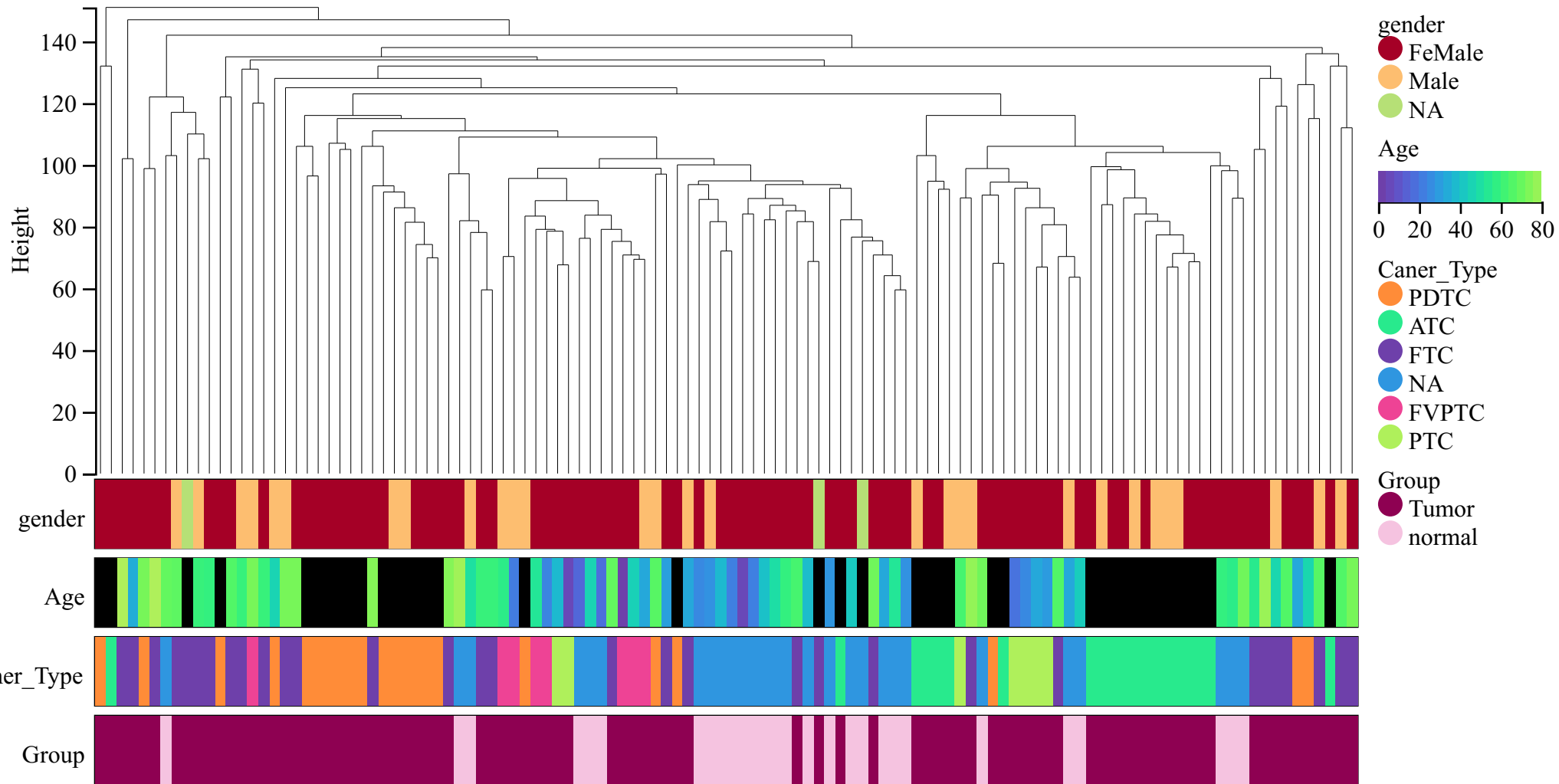

Supplement: Supplementary file 1 — Supplementary Information. [file 41598_2024_58943_MOESM1_ESM.zip › Raw data/Raw data/2.WGCNA/样本聚类.pdf]

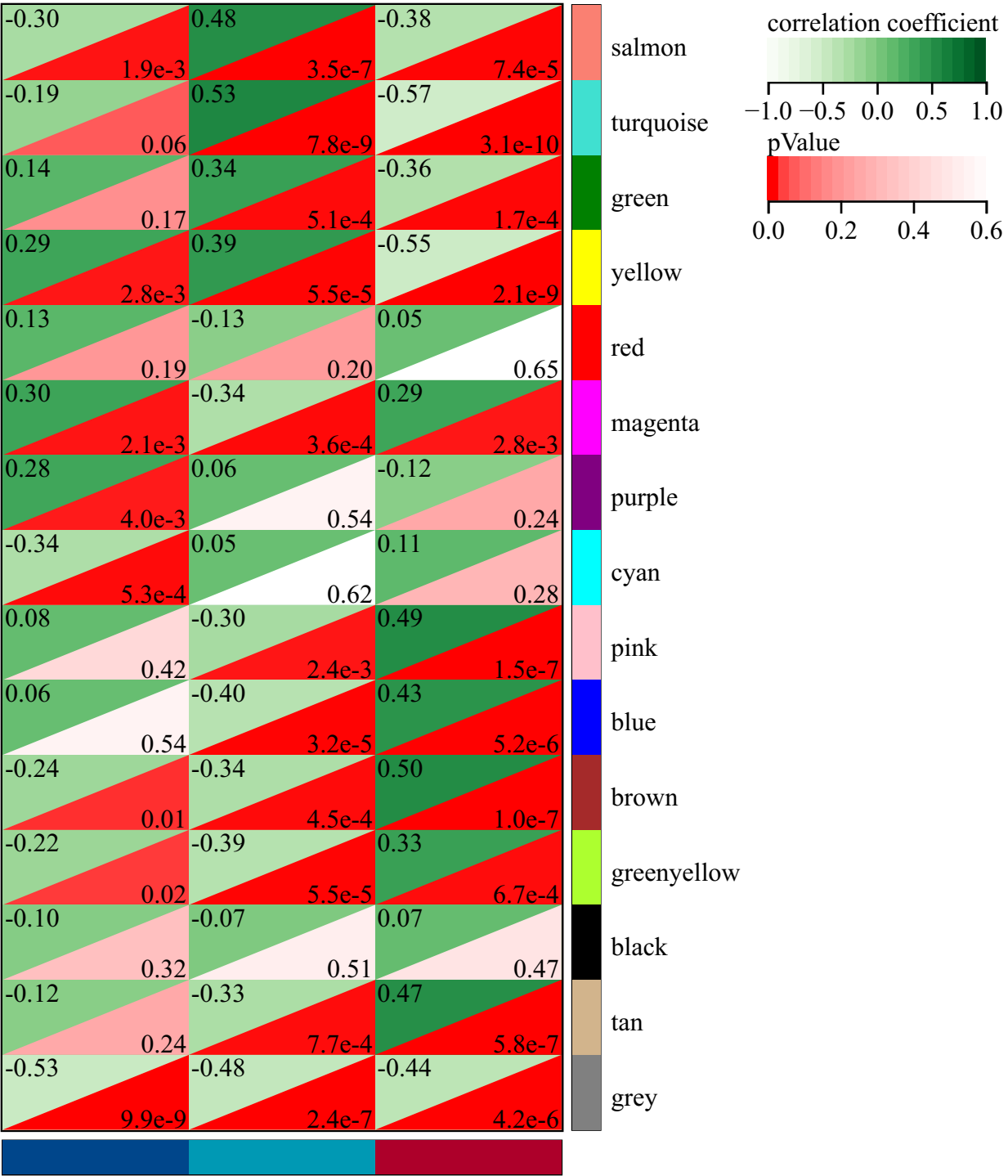

Age

Cancer\_Type(ATC)

Cancer\_Type(PDTC)

Supplement: Supplementary file 1 — Supplementary Information. [file 41598_2024_58943_MOESM1_ESM.zip › Raw data/Raw data/2.WGCNA/模块与表型相关性热图.pdf]

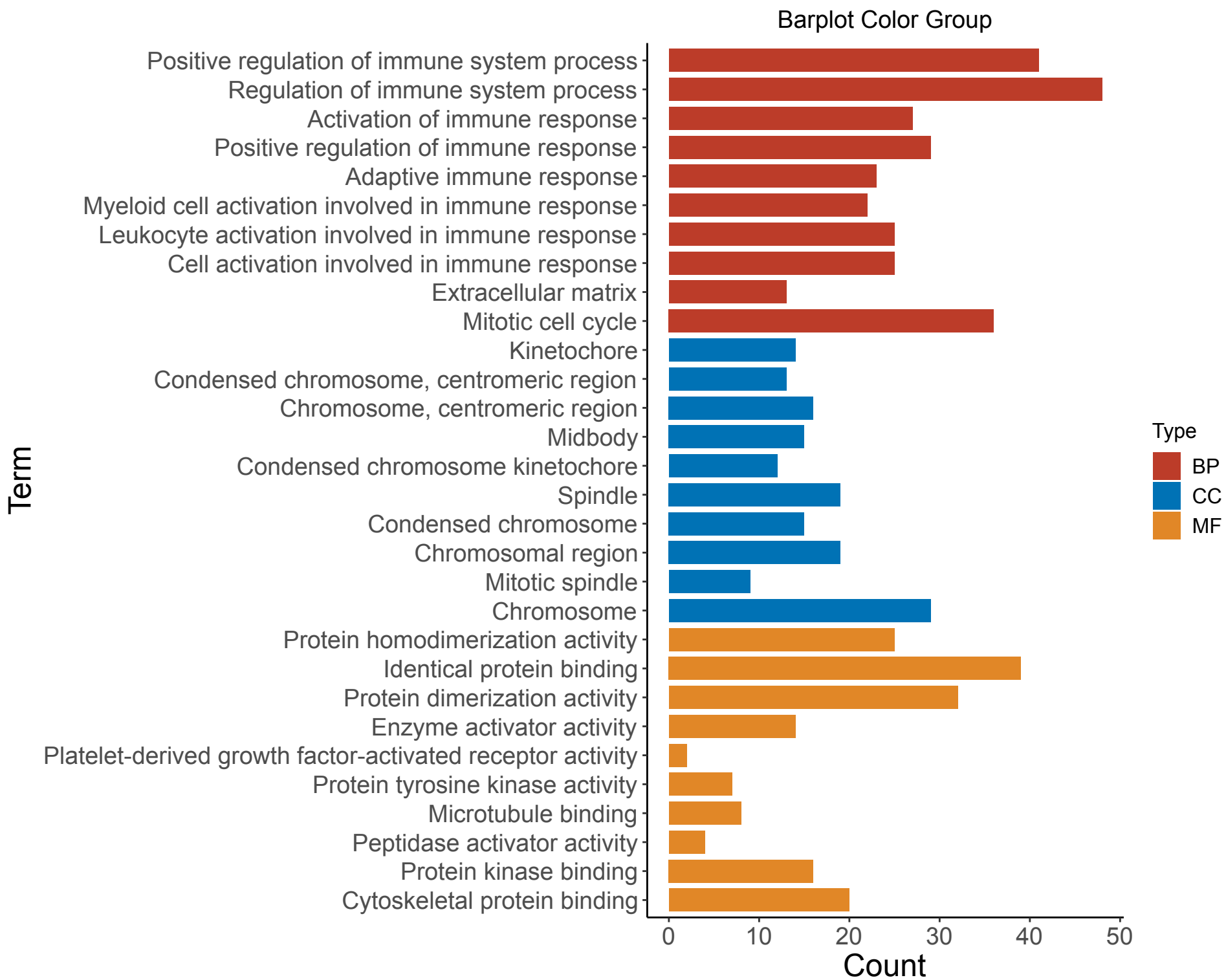

Supplement: Supplementary file 1 — Supplementary Information. [file 41598_2024_58943_MOESM1_ESM.zip › Raw data/Raw data/3.GO&KEGG/GO.pdf]

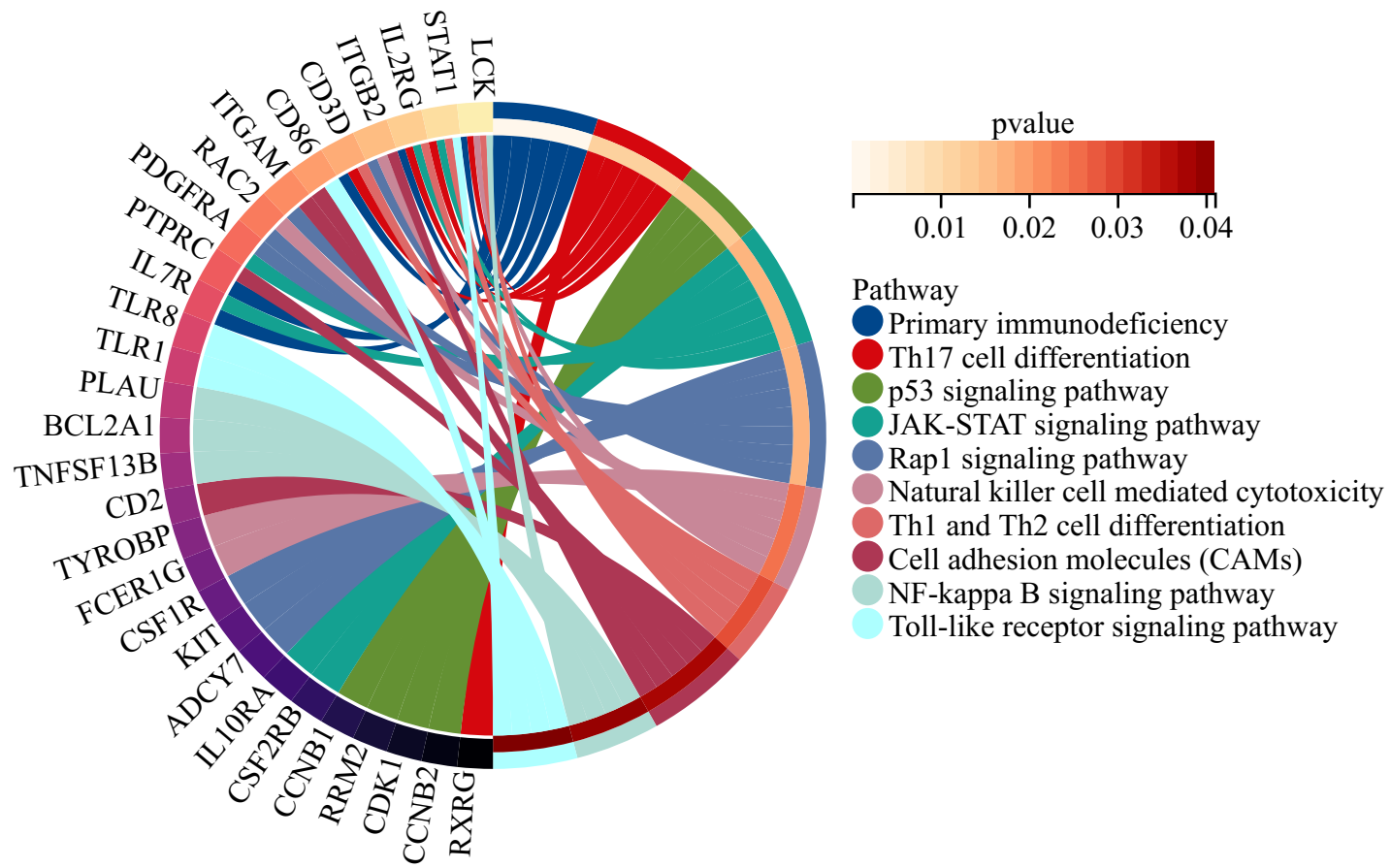

Supplement: Supplementary file 1 — Supplementary Information. [file 41598_2024_58943_MOESM1_ESM.zip › Raw data/Raw data/3.GO&KEGG/kegg.pdf]

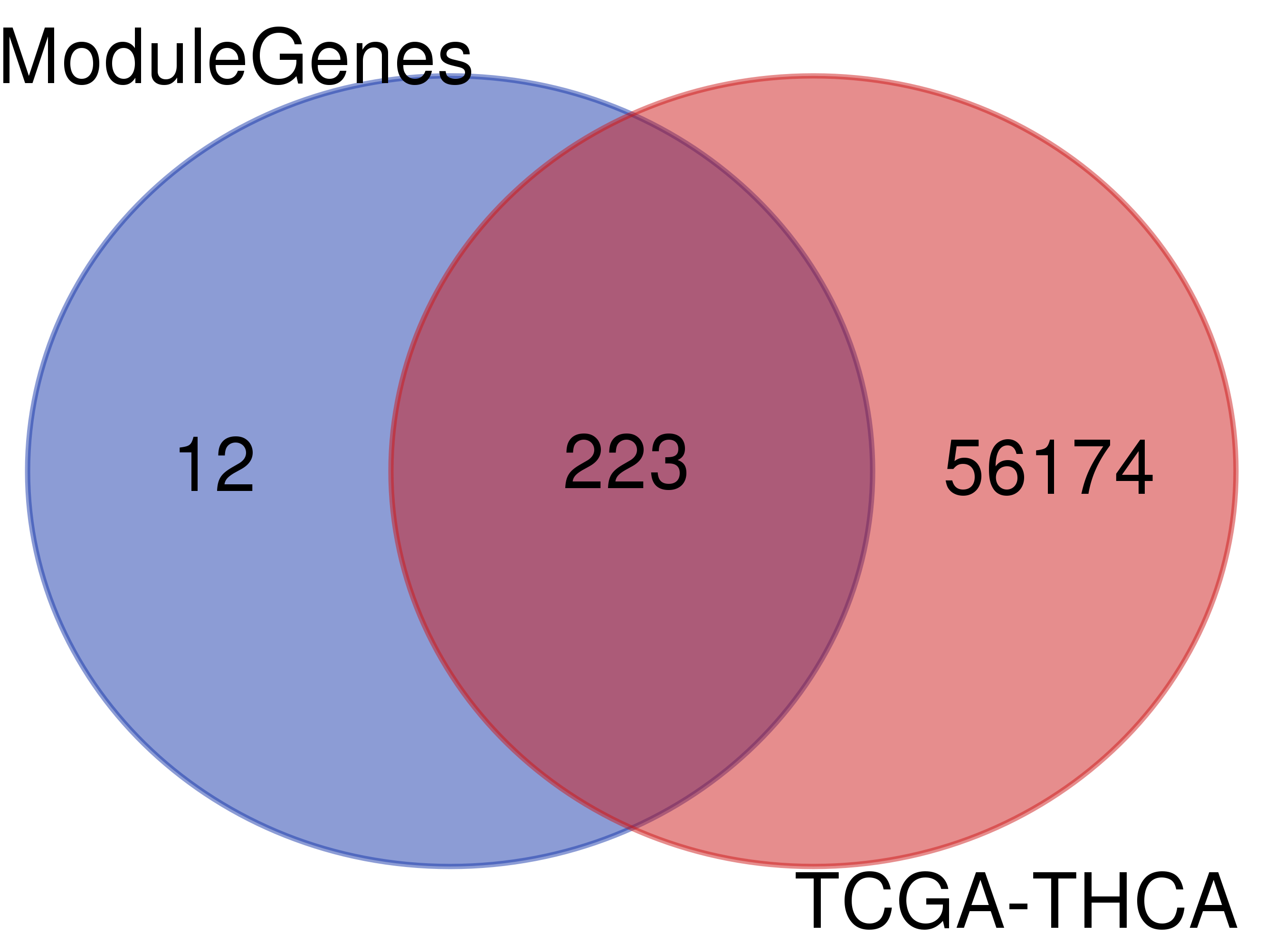

Supplement: Supplementary file 1 — Supplementary Information. [file 41598_2024_58943_MOESM1_ESM.zip › Raw data/Raw data/3.GO&KEGG/venn.png]

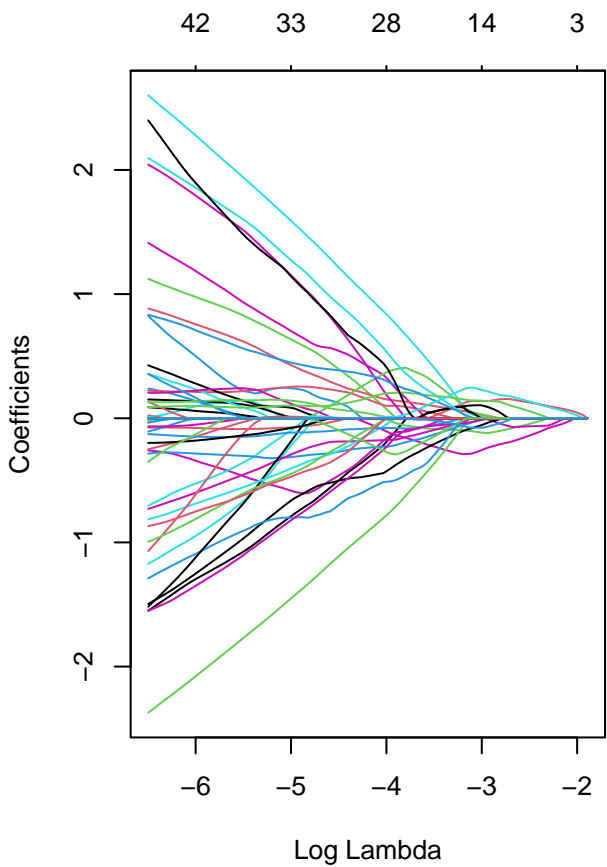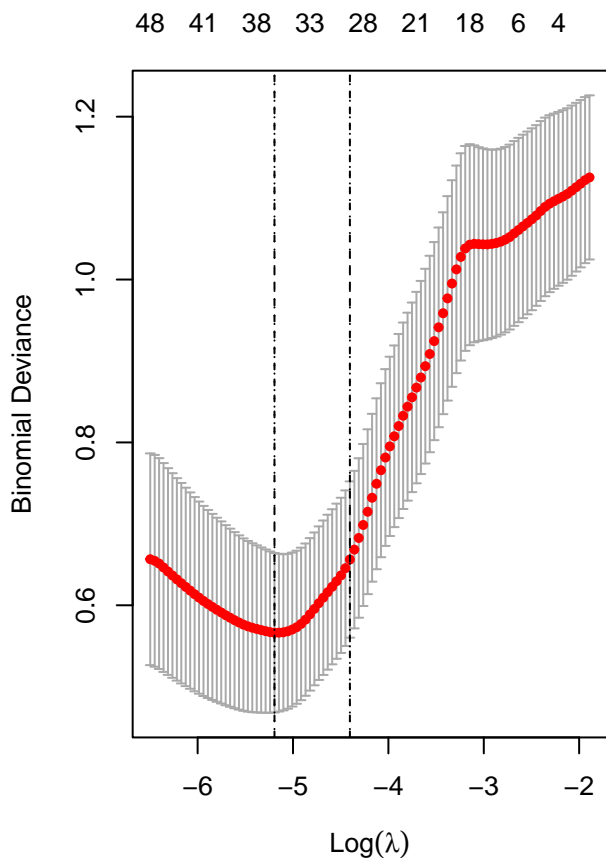

Supplement: Supplementary file 1 — Supplementary Information. [file 41598_2024_58943_MOESM1_ESM.zip › Raw data/Raw data/4. LASSO/Merge/lasso.pdf]

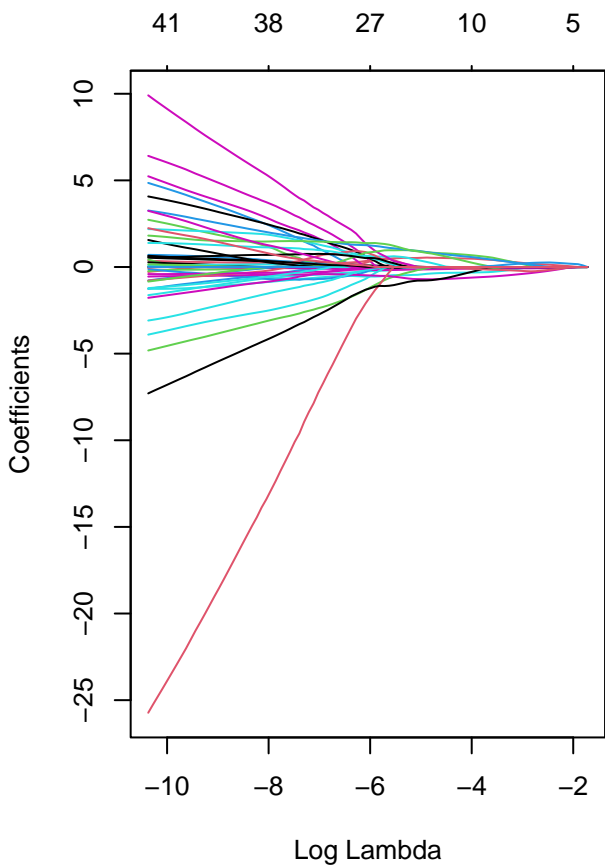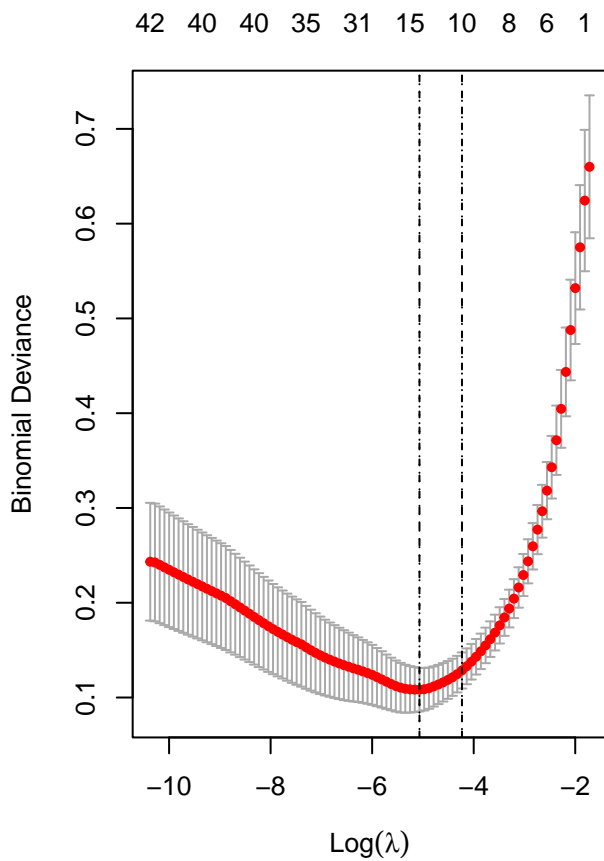

Supplement: Supplementary file 1 — Supplementary Information. [file 41598_2024_58943_MOESM1_ESM.zip › Raw data/Raw data/4. LASSO/TCGA/lasso.pdf]

# Venn Plot

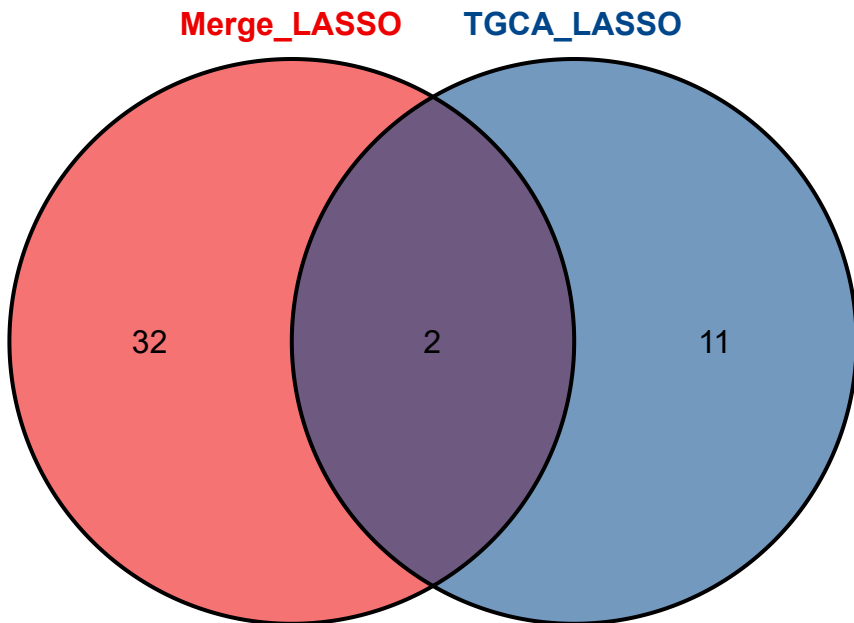

Supplement: Supplementary file 1 — Supplementary Information. [file 41598_2024_58943_MOESM1_ESM.zip › Raw data/Raw data/4. LASSO/venn.pdf]

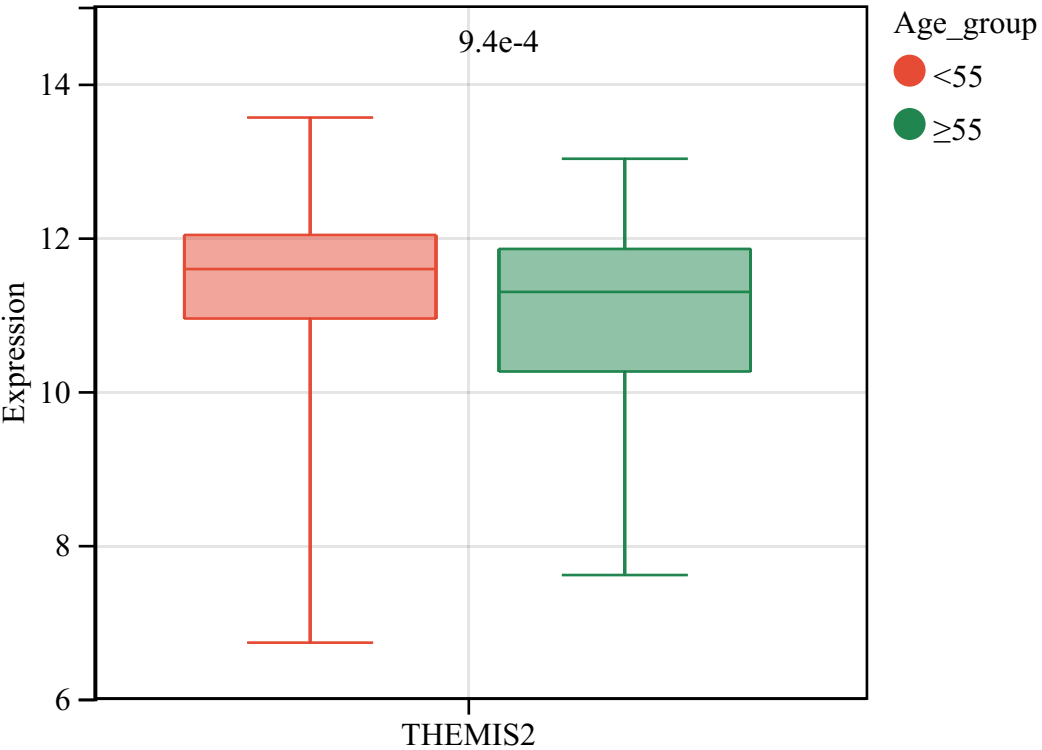

Supplement: Supplementary file 1 — Supplementary Information. [file 41598_2024_58943_MOESM1_ESM.zip › Raw data/Raw data/5. THEMIS2_analysis/Age.pdf]

Survival probability

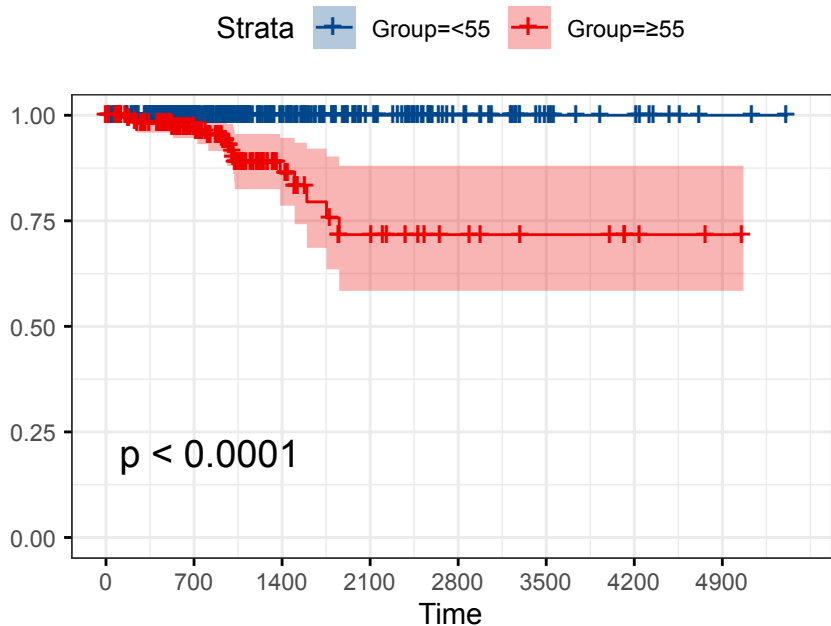

Number at risk

Strata

Group=&lt;55

Group= $\geq 55$ 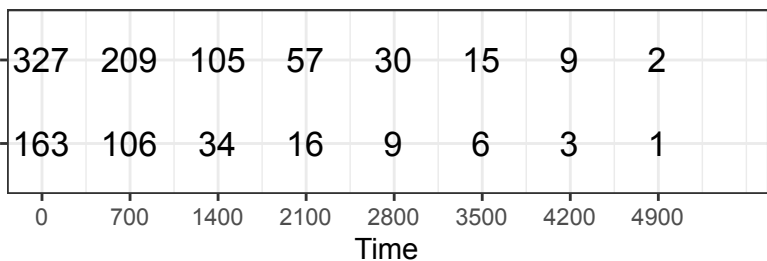

Number of censoring

n.censor

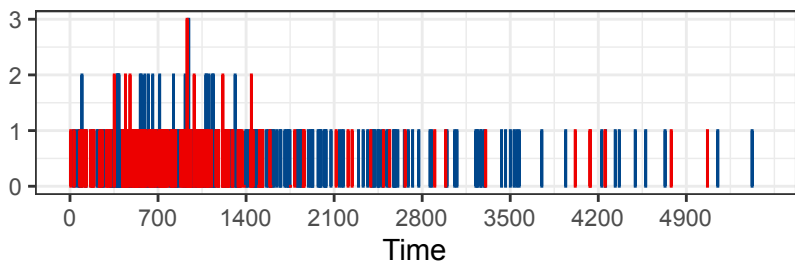

Supplement: Supplementary file 1 — Supplementary Information. [file 41598_2024_58943_MOESM1_ESM.zip › Raw data/Raw data/5. THEMIS2_analysis/Age_Survival_Analysis.pdf]

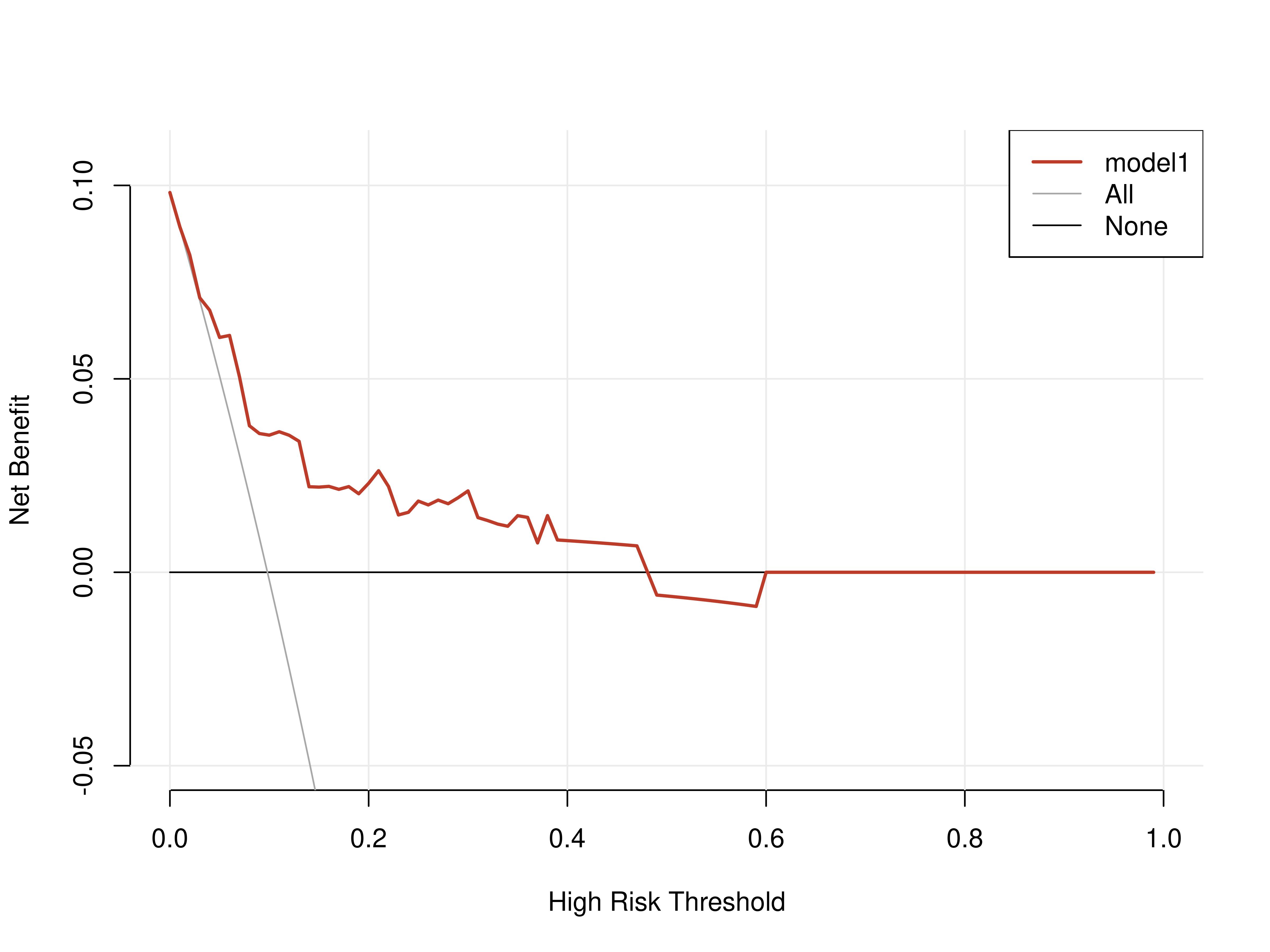

Supplement: Supplementary file 1 — Supplementary Information. [file 41598_2024_58943_MOESM1_ESM.zip › Raw data/Raw data/5. THEMIS2_analysis/DCA.jpg]

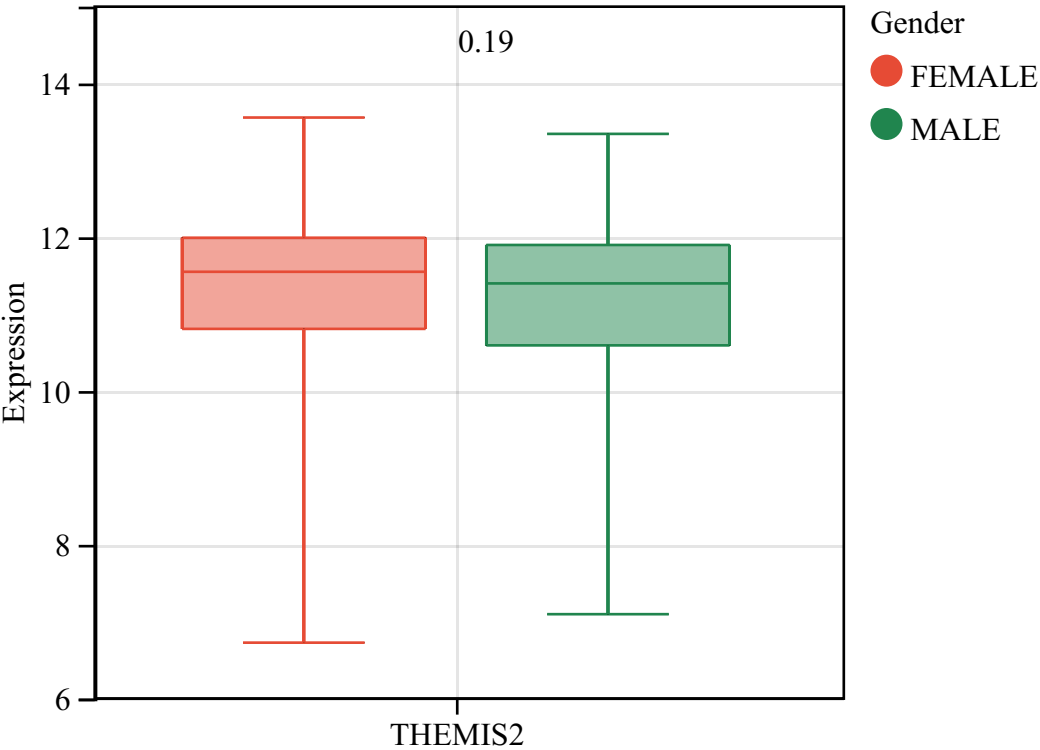

Supplement: Supplementary file 1 — Supplementary Information. [file 41598_2024_58943_MOESM1_ESM.zip › Raw data/Raw data/5. THEMIS2_analysis/Gender.pdf]

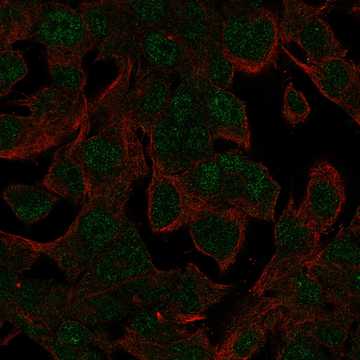

Supplement: Supplementary file 1 — Supplementary Information. [file 41598_2024_58943_MOESM1_ESM.zip › Raw data/Raw data/5. THEMIS2_analysis/Images/1.jpg]

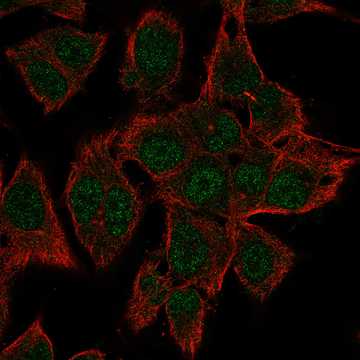

Supplement: Supplementary file 1 — Supplementary Information. [file 41598_2024_58943_MOESM1_ESM.zip › Raw data/Raw data/5. THEMIS2_analysis/Images/2.jpg]

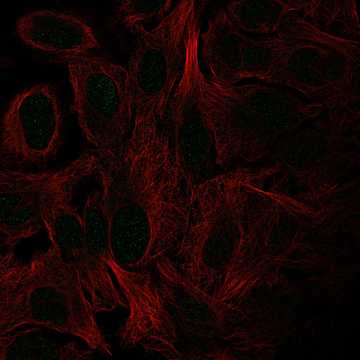

Supplement: Supplementary file 1 — Supplementary Information. [file 41598_2024_58943_MOESM1_ESM.zip › Raw data/Raw data/5. THEMIS2_analysis/Images/3.jpg]

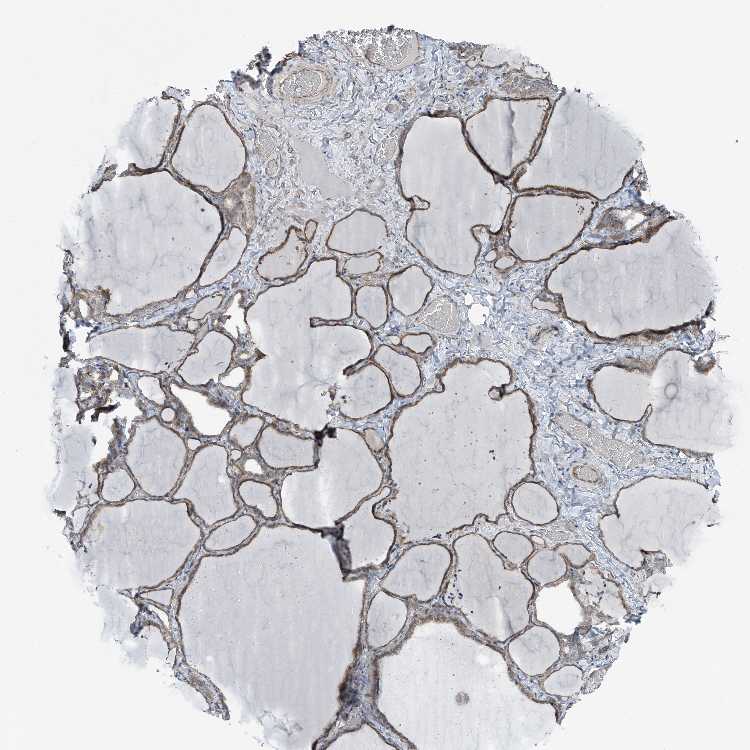

Supplement: Supplementary file 1 — Supplementary Information. [file 41598_2024_58943_MOESM1_ESM.zip › Raw data/Raw data/5. THEMIS2_analysis/Images/65313_B_1_5_medium.jpg]

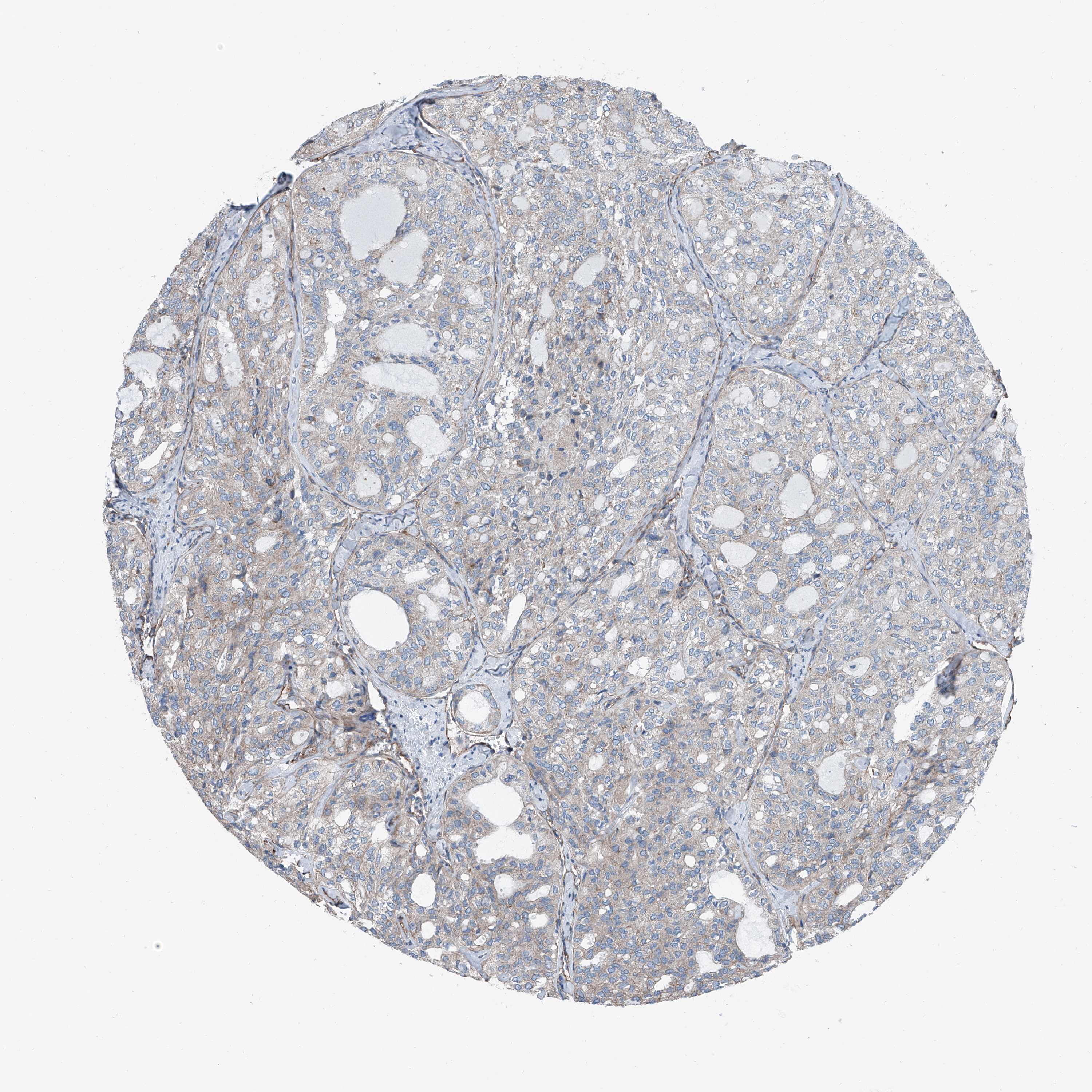

Supplement: Supplementary file 1 — Supplementary Information. [file 41598_2024_58943_MOESM1_ESM.zip › Raw data/Raw data/5. THEMIS2_analysis/Images/tumor.jpg]

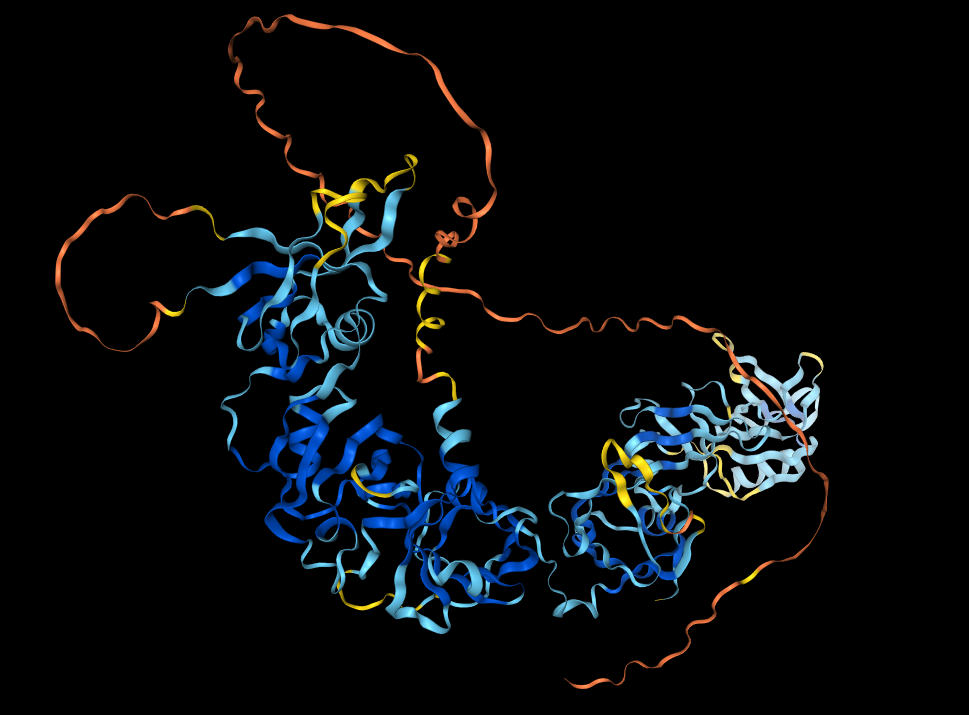

Supplement: Supplementary file 1 — Supplementary Information. [file 41598_2024_58943_MOESM1_ESM.zip › Raw data/Raw data/5. THEMIS2_analysis/Images/图片1.png]

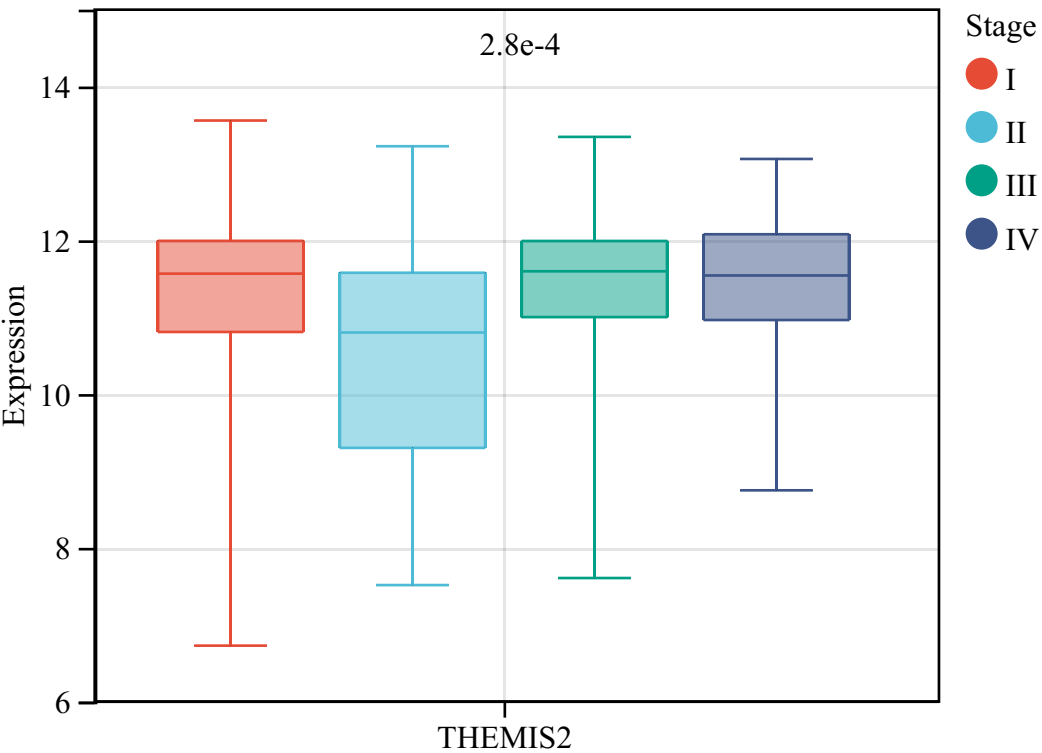

Supplement: Supplementary file 1 — Supplementary Information. [file 41598_2024_58943_MOESM1_ESM.zip › Raw data/Raw data/5. THEMIS2_analysis/Stage.pdf]

Survival probability

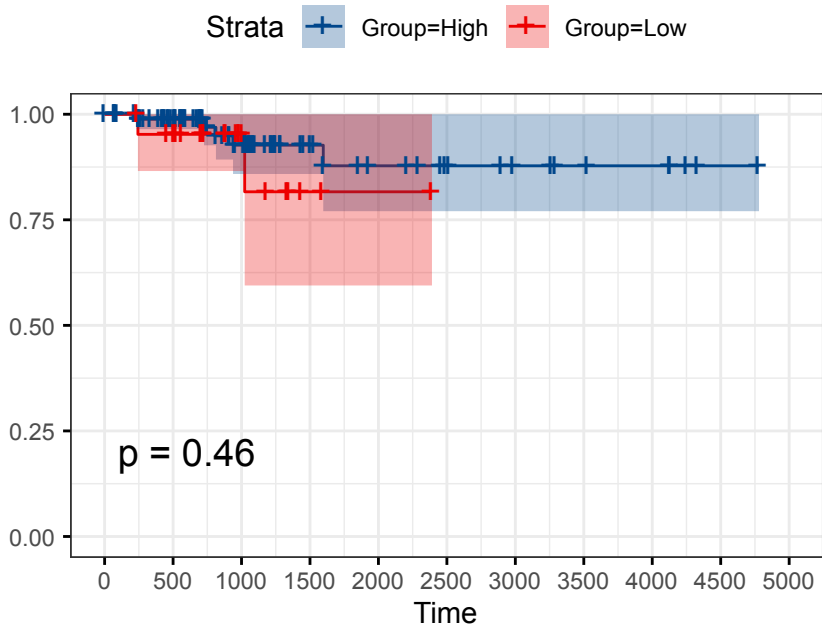

Number at risk

Strata

Group=High

Group=Low

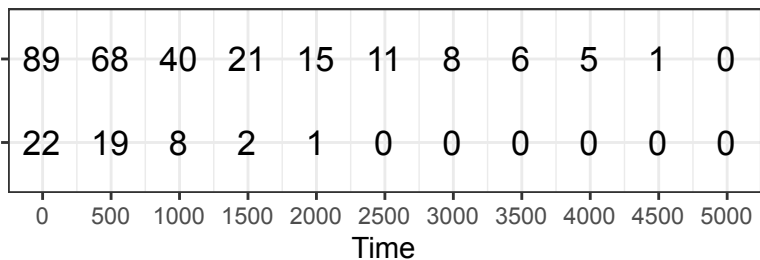

Number of censoring

n.censor

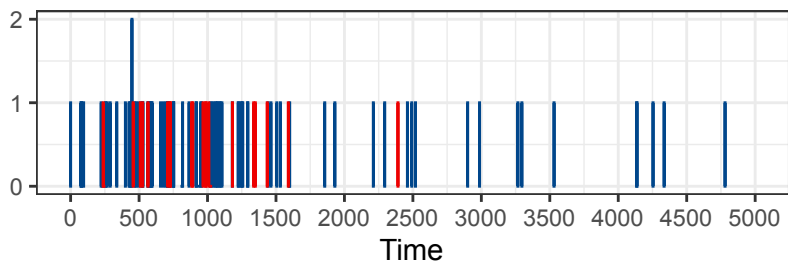

Supplement: Supplementary file 1 — Supplementary Information. [file 41598_2024_58943_MOESM1_ESM.zip › Raw data/Raw data/5. THEMIS2_analysis/stageIII_Survival_Analysis.pdf]

Survival probability

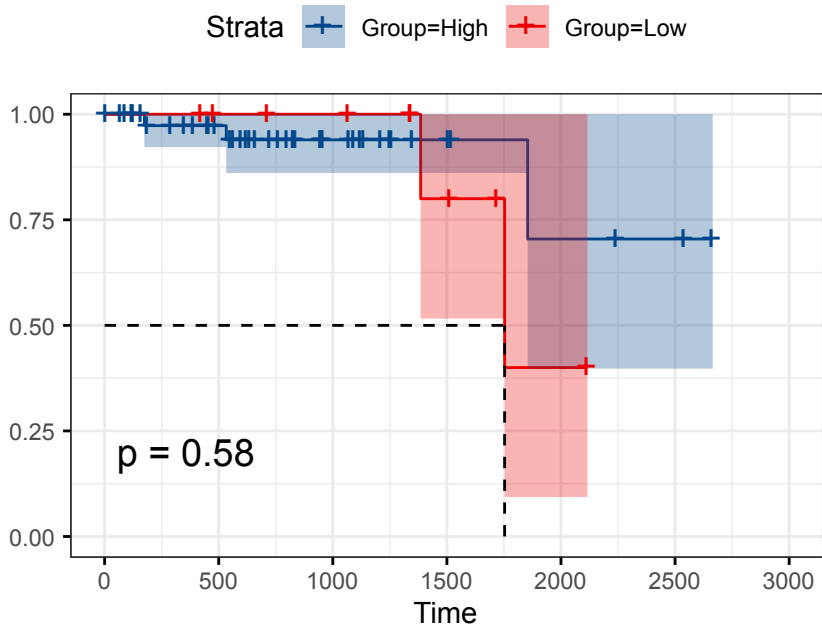

Number at risk

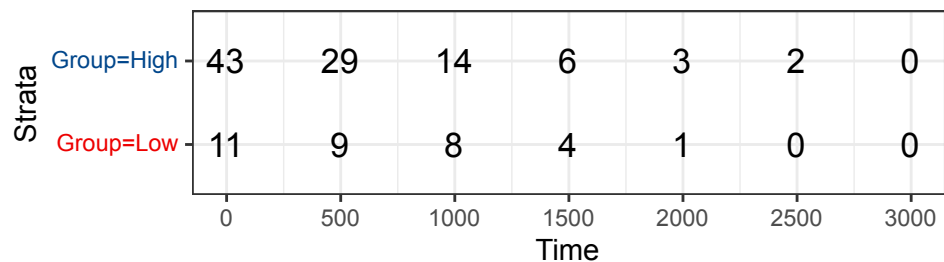

Number of censoring

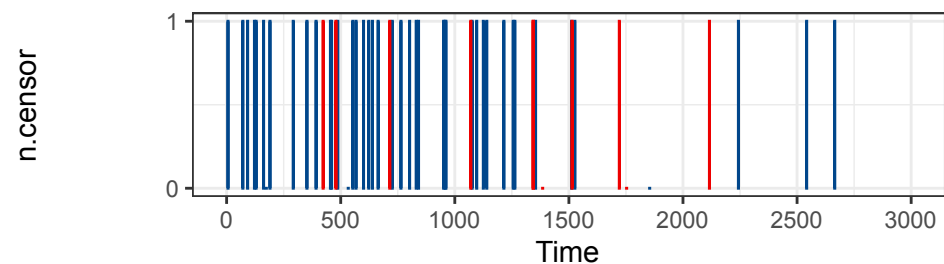

Supplement: Supplementary file 1 — Supplementary Information. [file 41598_2024_58943_MOESM1_ESM.zip › Raw data/Raw data/5. THEMIS2_analysis/stageIV_Survival_Analysis.pdf]

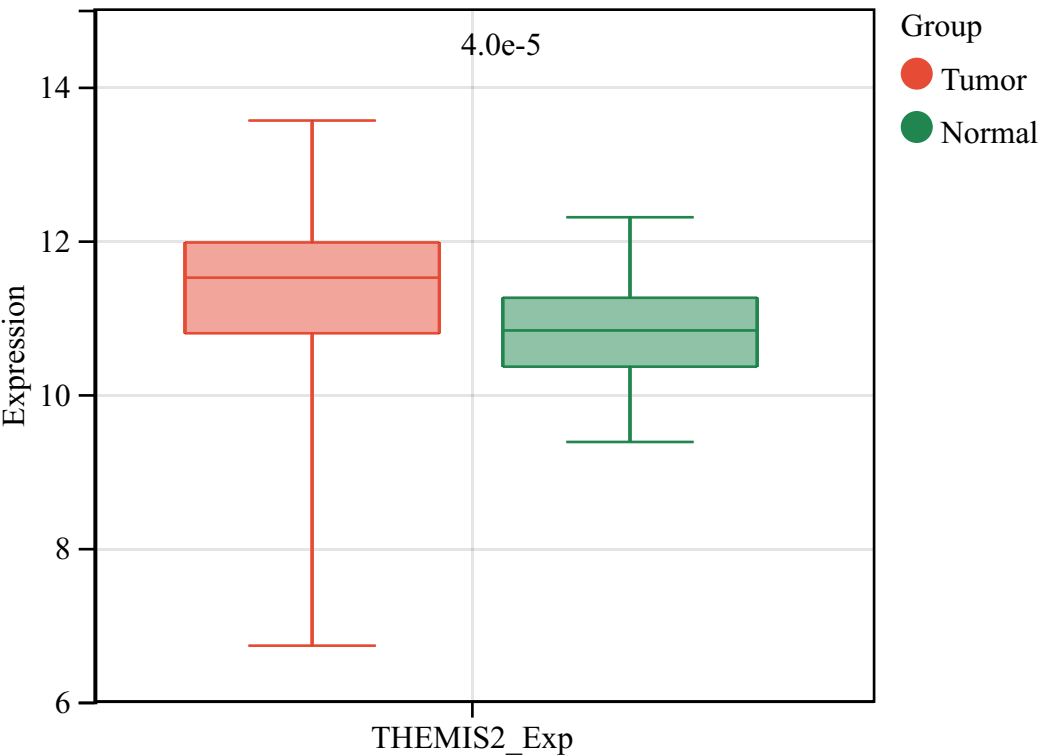

Supplement: Supplementary file 1 — Supplementary Information. [file 41598_2024_58943_MOESM1_ESM.zip › Raw data/Raw data/5. THEMIS2_analysis/TCGA_THEMIS2_exp.pdf]

# THEMIS2

Sensitivity

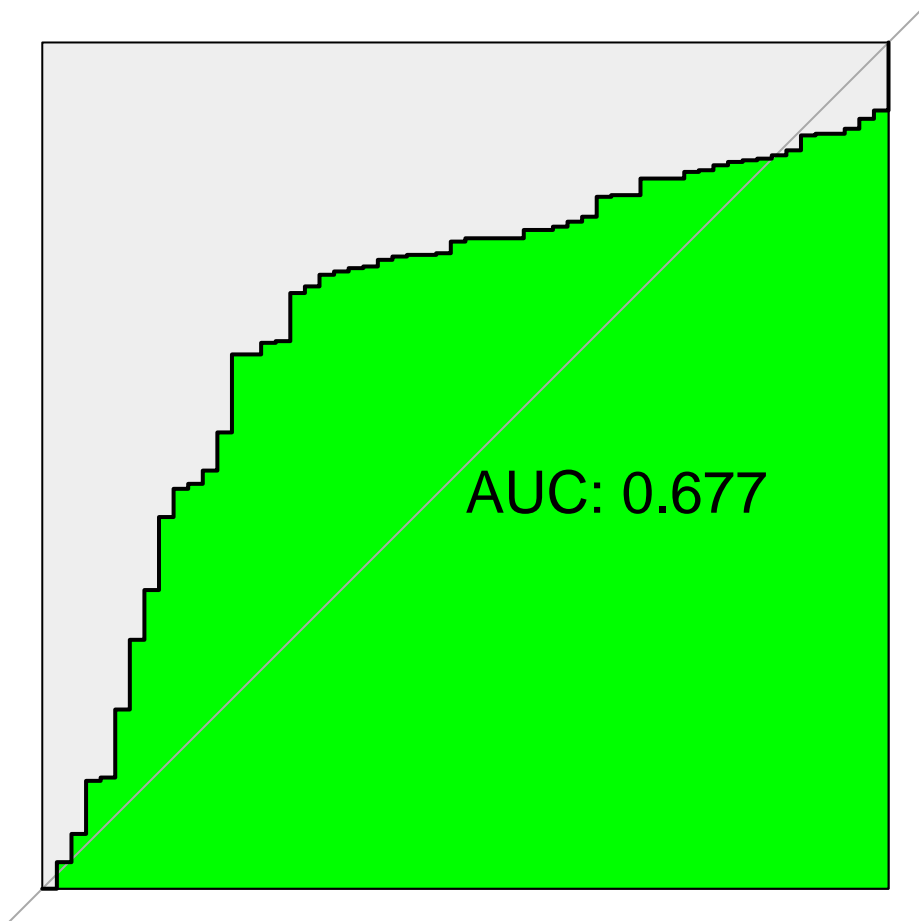

AUC: 0.677

Specificity

Supplement: Supplementary file 1 — Supplementary Information. [file 41598_2024_58943_MOESM1_ESM.zip › Raw data/Raw data/5. THEMIS2_analysis/THEMIS2_ROC.pdf]

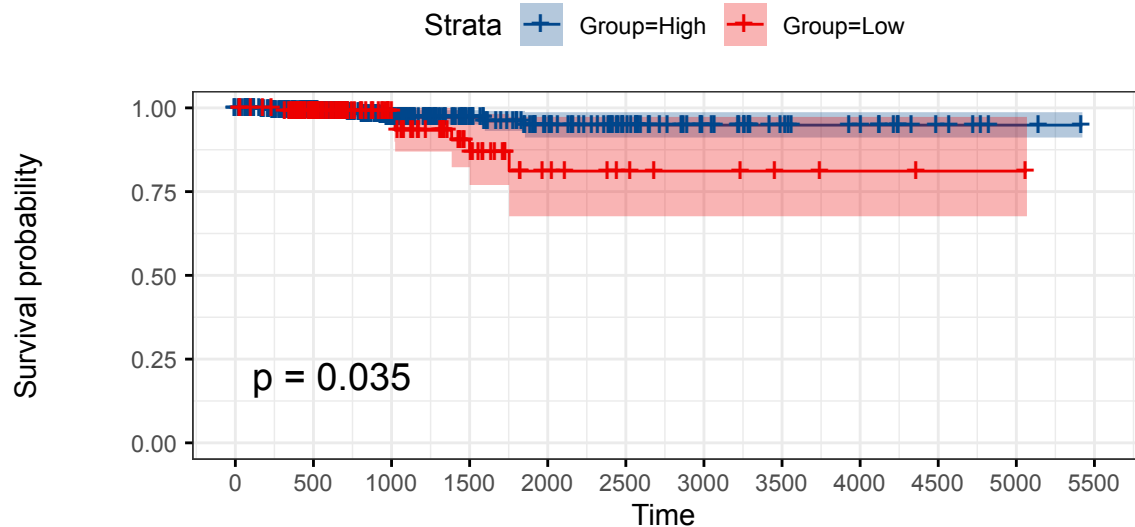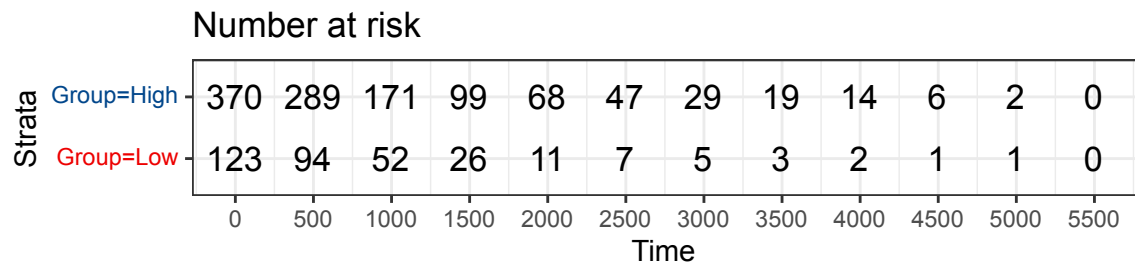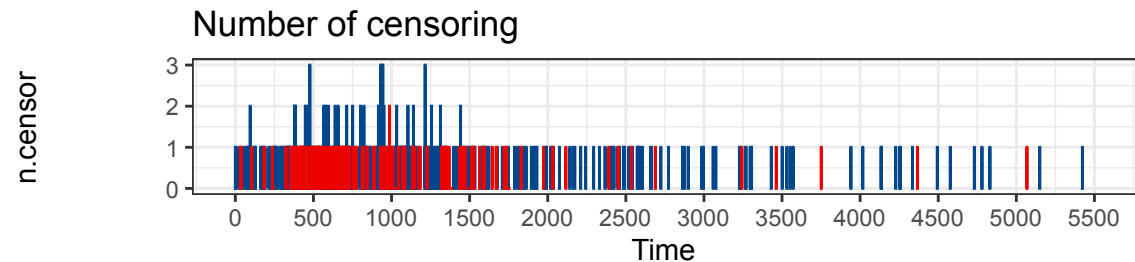

Supplement: Supplementary file 1 — Supplementary Information. [file 41598_2024_58943_MOESM1_ESM.zip › Raw data/Raw data/5. THEMIS2_analysis/THEMIS2_Survival_Analysis.pdf]

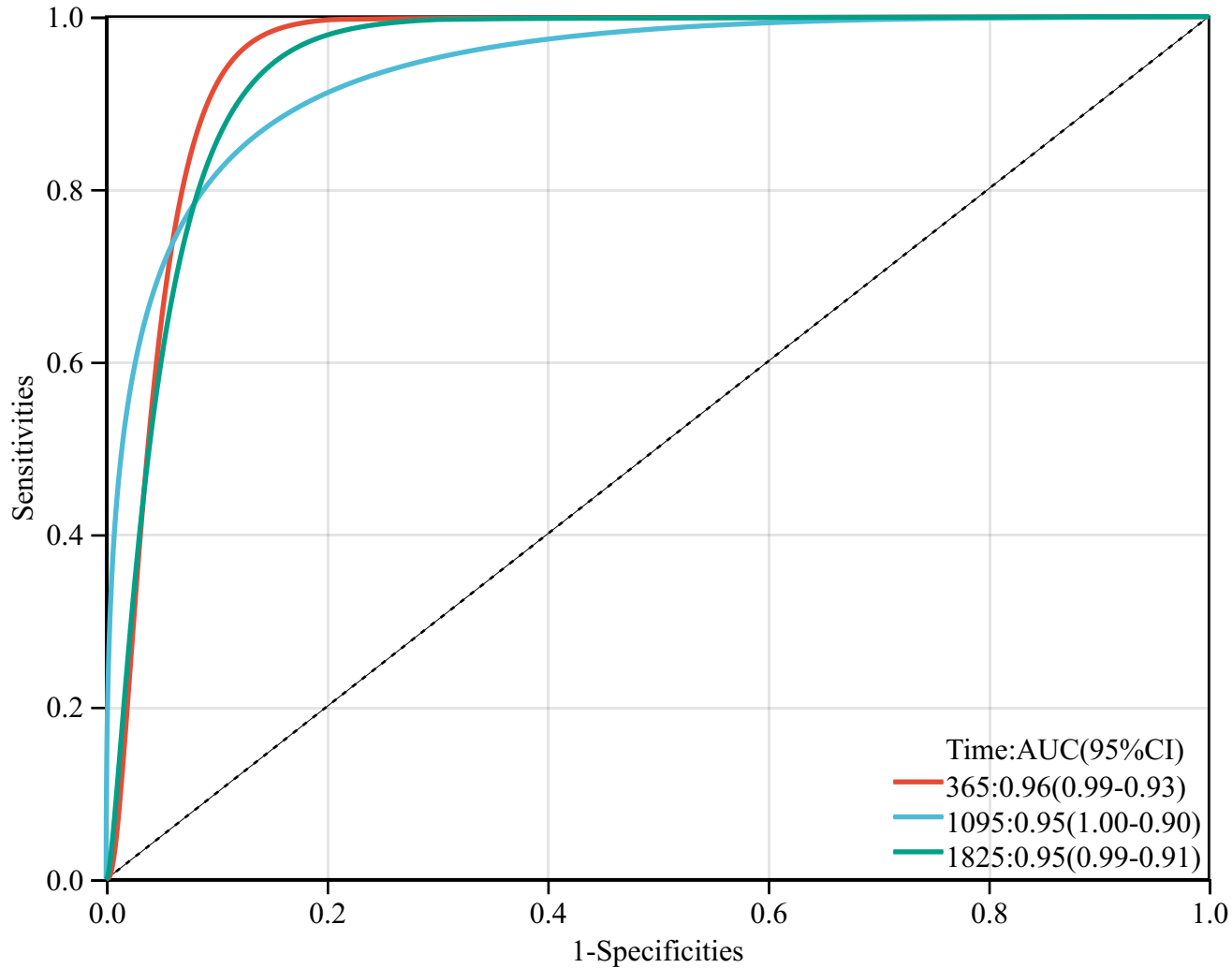

Supplement: Supplementary file 1 — Supplementary Information. [file 41598_2024_58943_MOESM1_ESM.zip › Raw data/Raw data/5. THEMIS2_analysis/三指标.pdf]

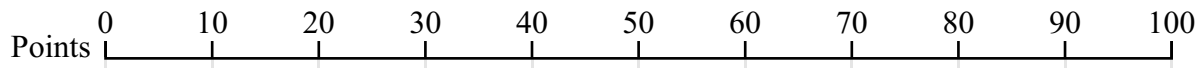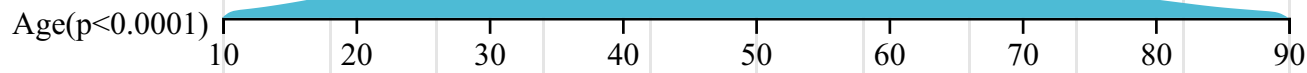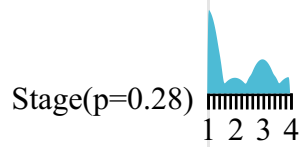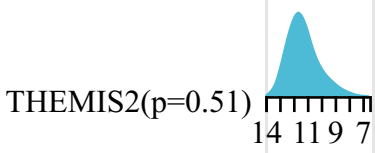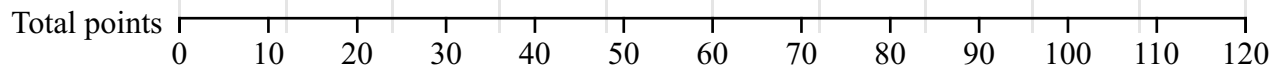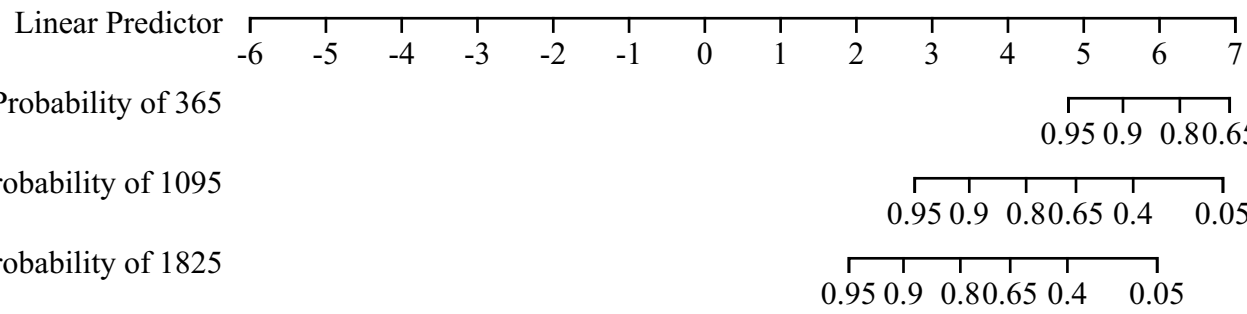

Supplement: Supplementary file 1 — Supplementary Information. [file 41598_2024_58943_MOESM1_ESM.zip › Raw data/Raw data/5. THEMIS2_analysis/列线图.pdf]

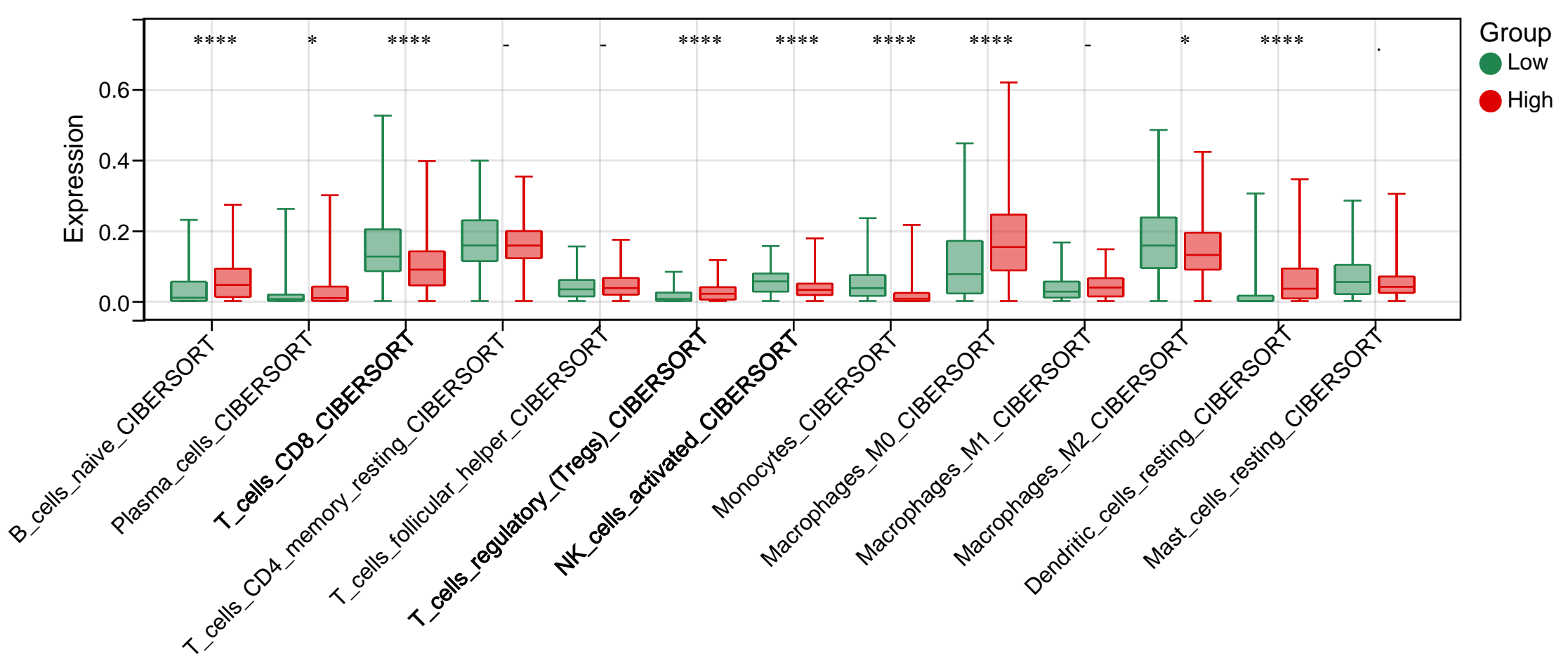

Supplement: Supplementary file 1 — Supplementary Information. [file 41598_2024_58943_MOESM1_ESM.zip › Raw data/Raw data/6. 免疫浸润/CIBERSORT.pdf]

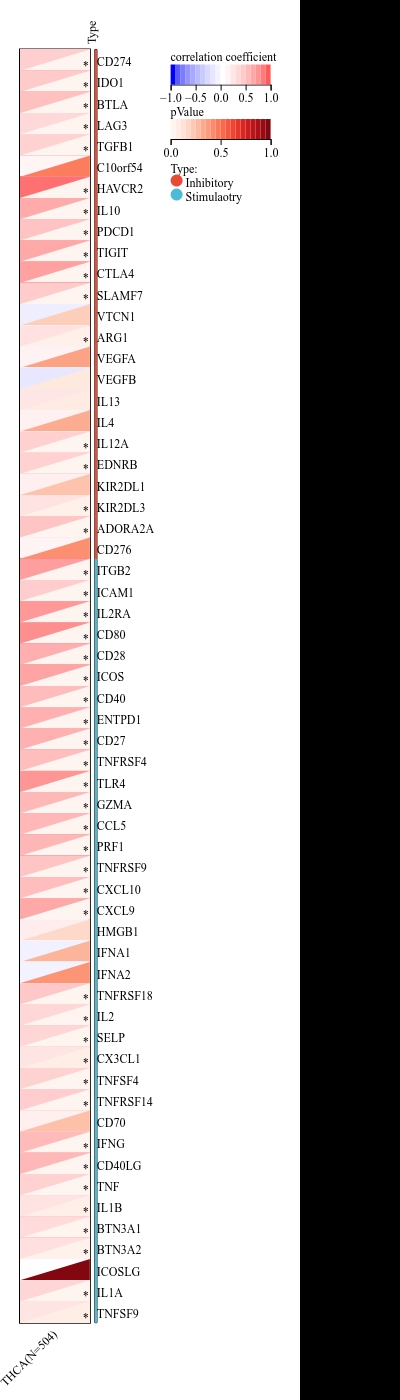

Supplement: Supplementary file 1 — Supplementary Information. [file 41598_2024_58943_MOESM1_ESM.zip › Raw data/Raw data/7. 免疫检查点分析/THCA_免疫检查点.jpeg]

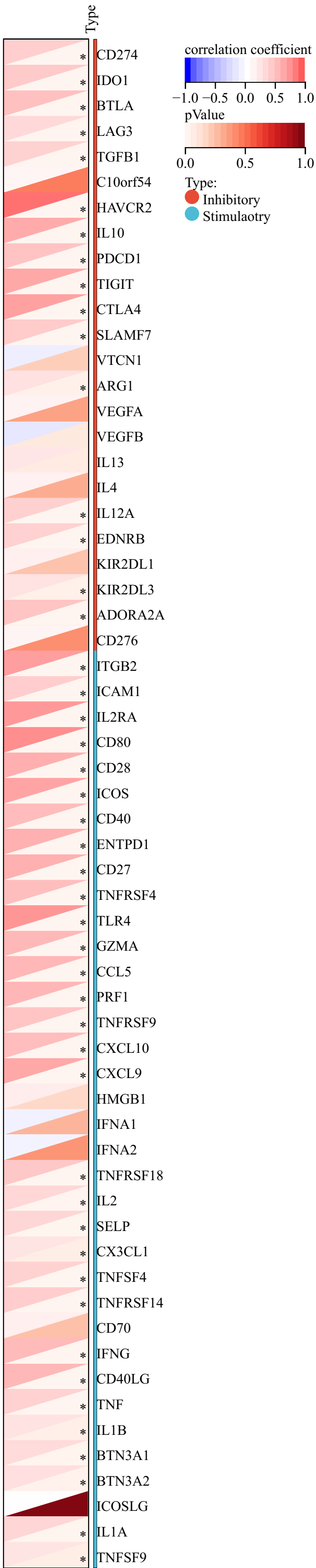

Supplement: Supplementary file 1 — Supplementary Information. [file 41598_2024_58943_MOESM1_ESM.zip › Raw data/Raw data/7. 免疫检查点分析/THCA_免疫检查点.pdf]

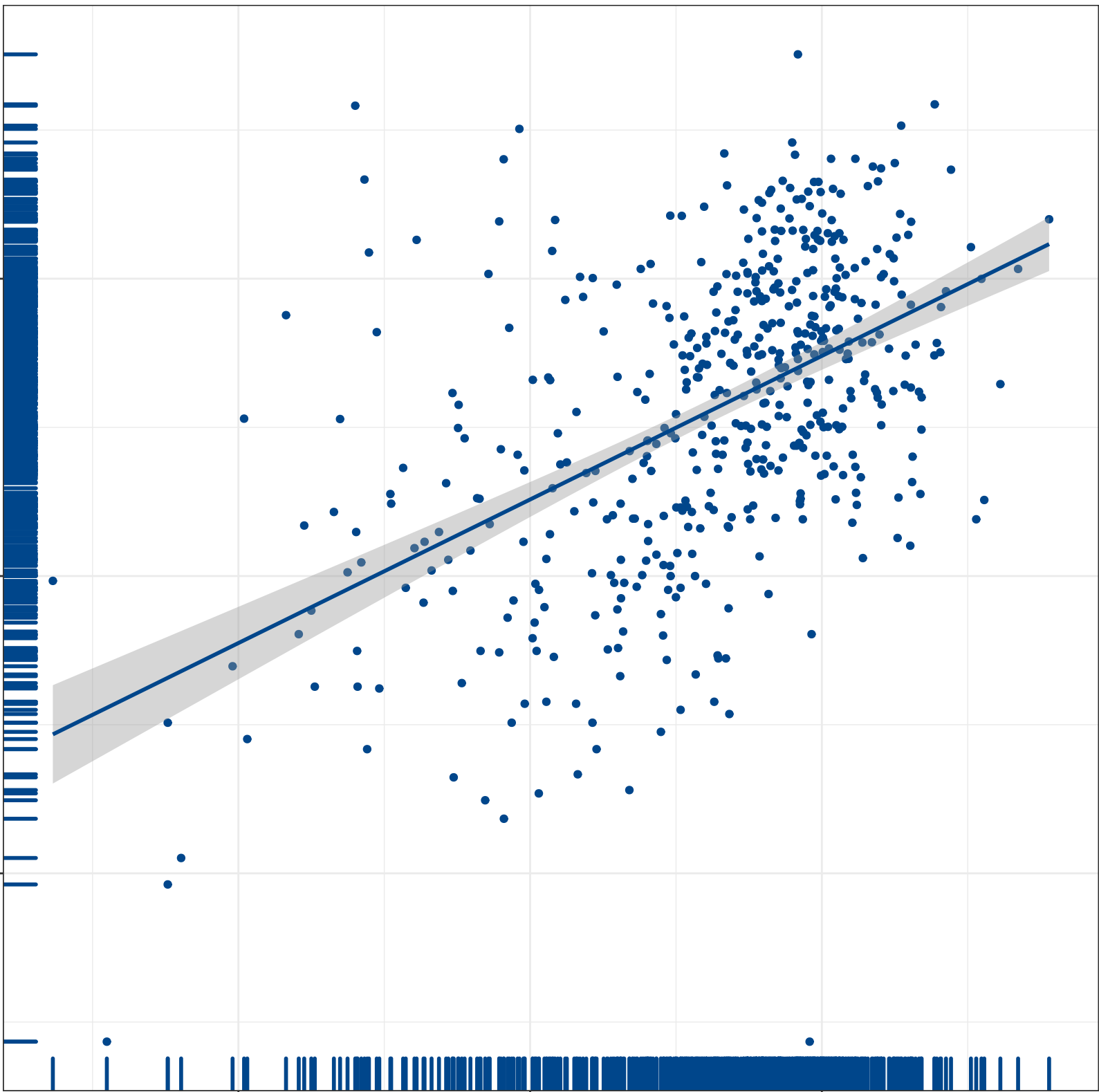

Supplement: Supplementary file 1 — Supplementary Information. [file 41598_2024_58943_MOESM1_ESM.zip › Raw data/Raw data/7. 免疫检查点分析/相关性分析/1.pdf]

Line Reguression Plot

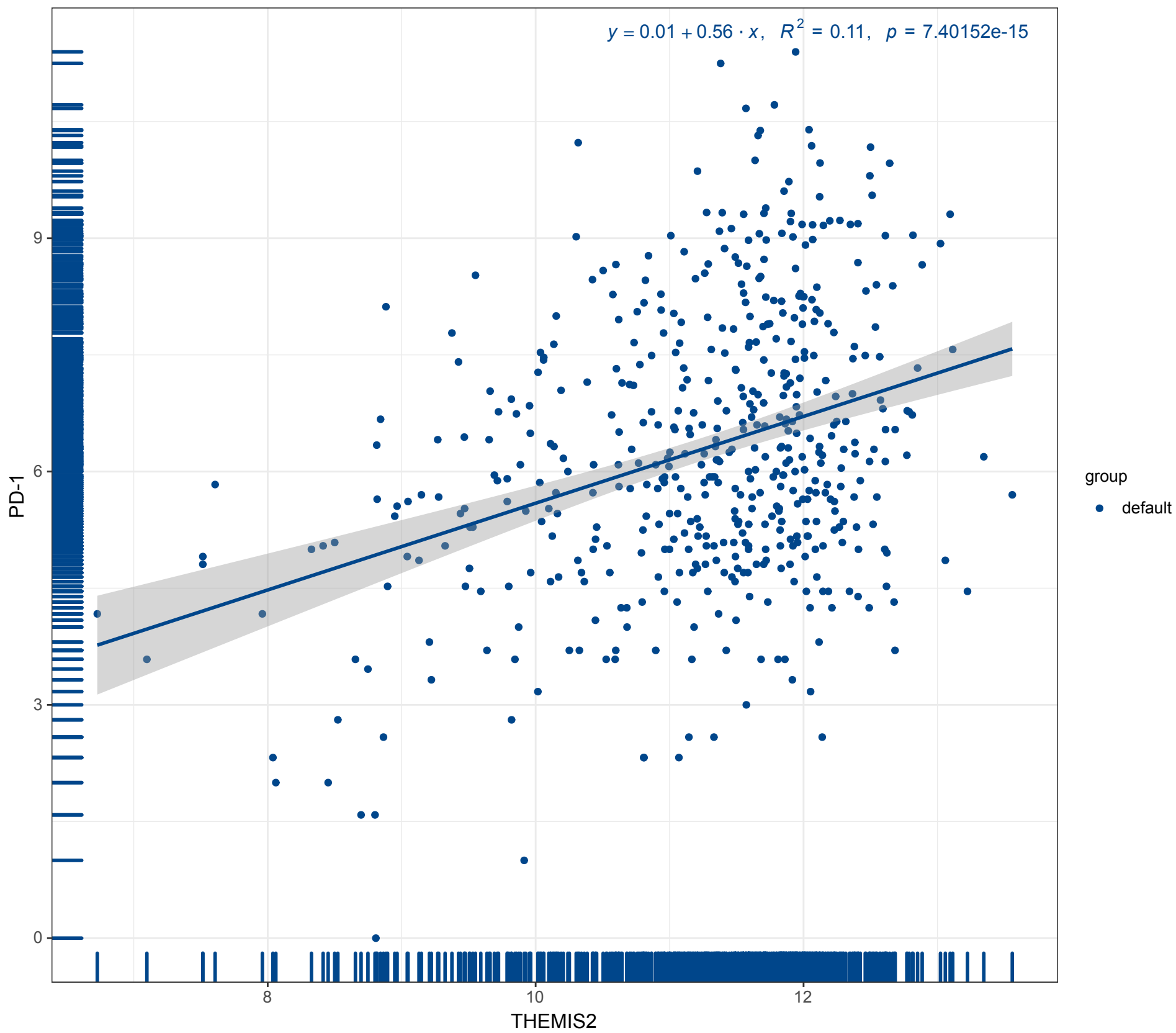

Supplement: Supplementary file 1 — Supplementary Information. [file 41598_2024_58943_MOESM1_ESM.zip › Raw data/Raw data/7. 免疫检查点分析/相关性分析/2.pdf]

Line Reguression Plot

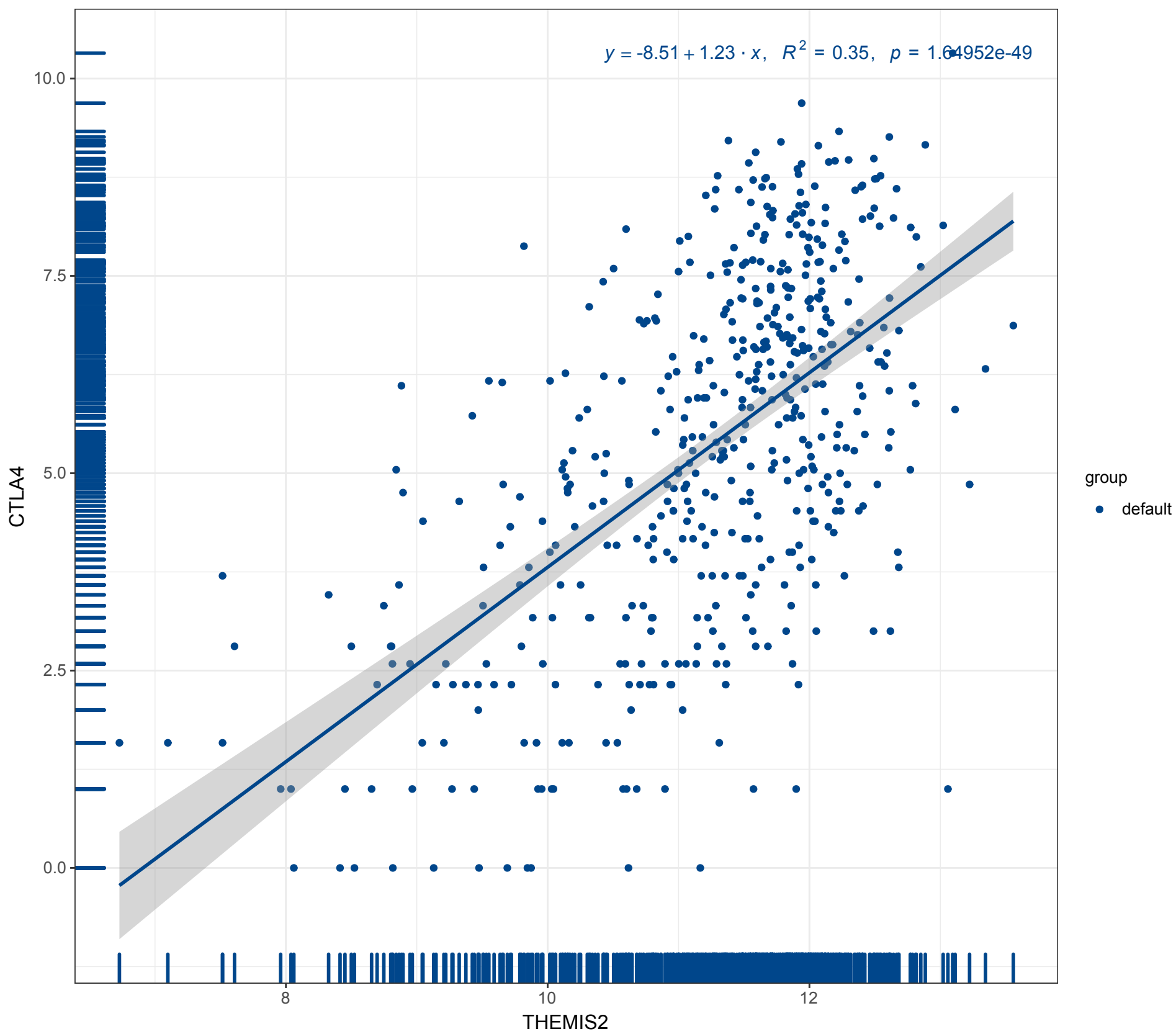

Supplement: Supplementary file 1 — Supplementary Information. [file 41598_2024_58943_MOESM1_ESM.zip › Raw data/Raw data/7. 免疫检查点分析/相关性分析/3.pdf]

Line Reguression Plot

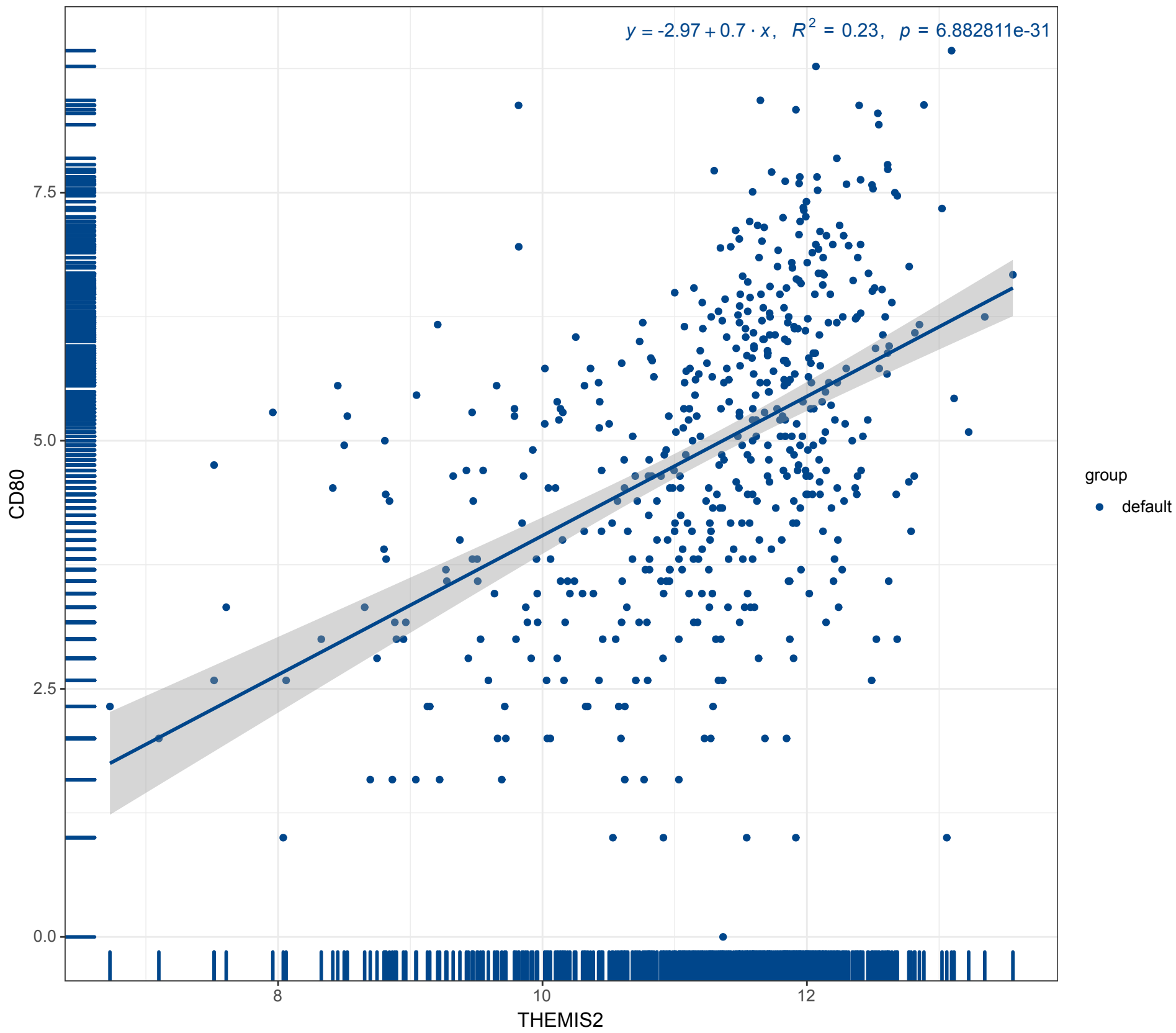

Supplement: Supplementary file 1 — Supplementary Information. [file 41598_2024_58943_MOESM1_ESM.zip › Raw data/Raw data/7. 免疫检查点分析/相关性分析/4.pdf]

Line Reguression Plot

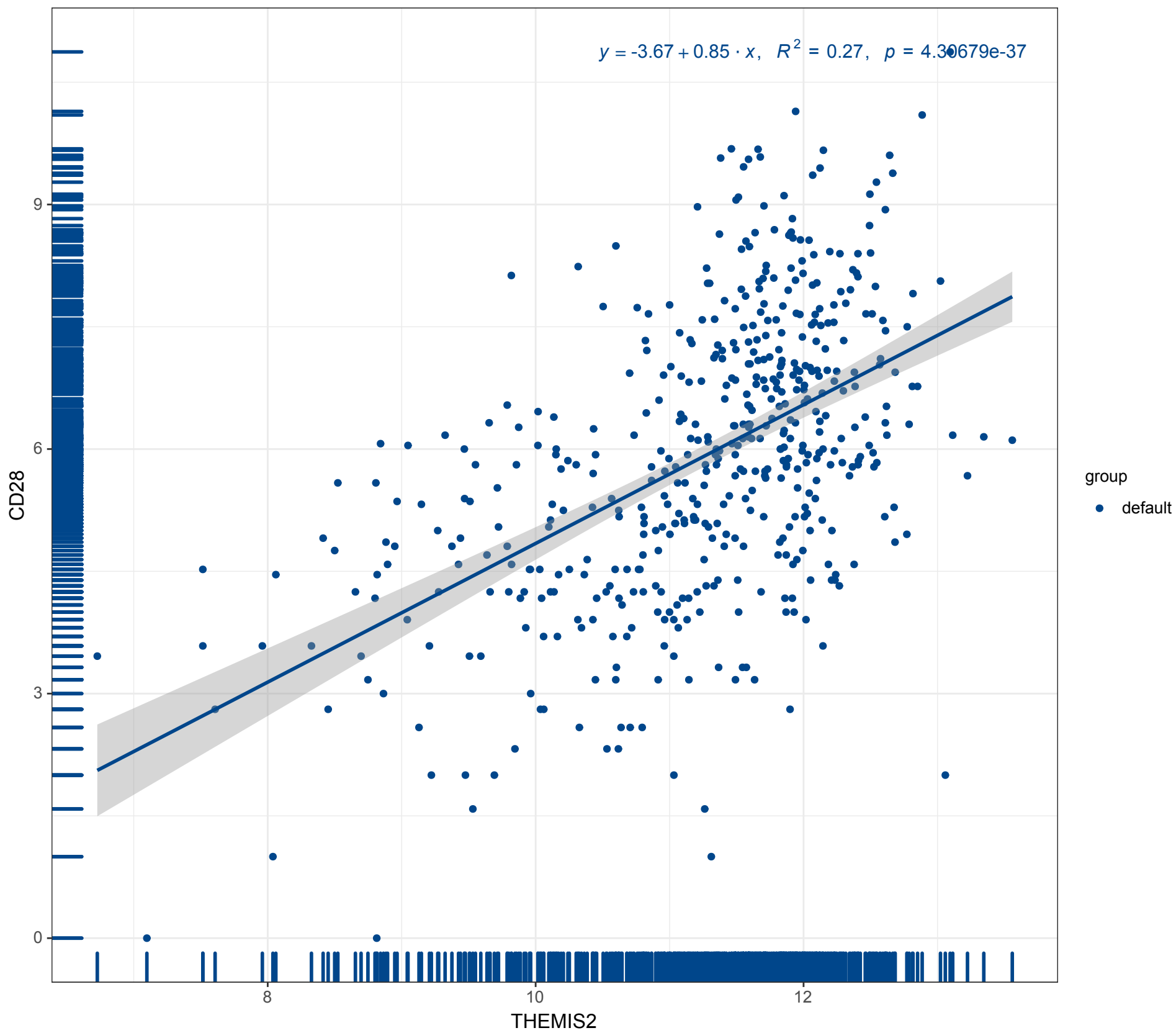

Supplement: Supplementary file 1 — Supplementary Information. [file 41598_2024_58943_MOESM1_ESM.zip › Raw data/Raw data/7. 免疫检查点分析/相关性分析/5.pdf]

Line Reguression Plot

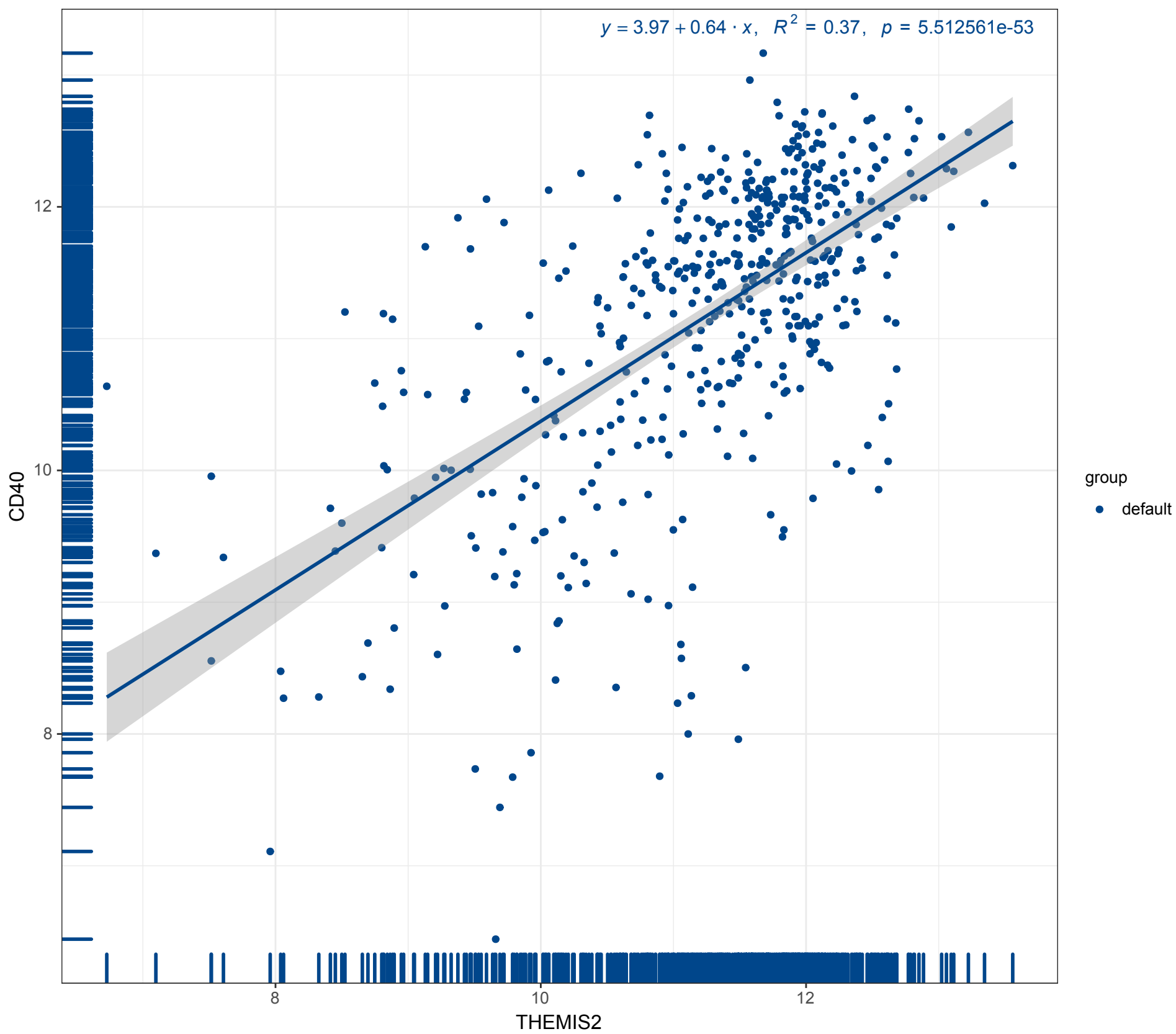

Supplement: Supplementary file 1 — Supplementary Information. [file 41598_2024_58943_MOESM1_ESM.zip › Raw data/Raw data/7. 免疫检查点分析/相关性分析/6.pdf]

Line Reguression Plot

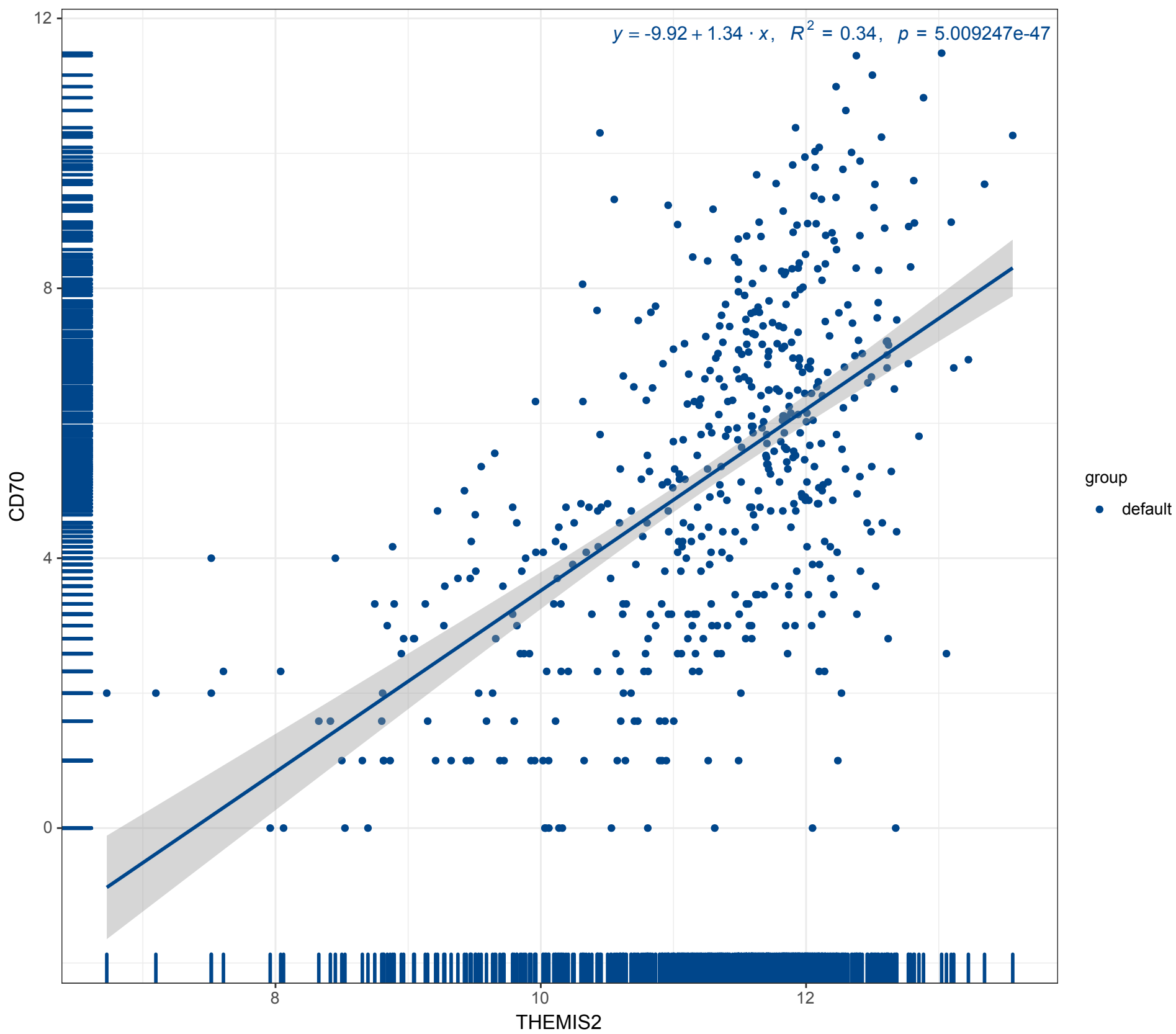

Supplement: Supplementary file 1 — Supplementary Information. [file 41598_2024_58943_MOESM1_ESM.zip › Raw data/Raw data/7. 免疫检查点分析/相关性分析/7.pdf]

Line Regression Plot

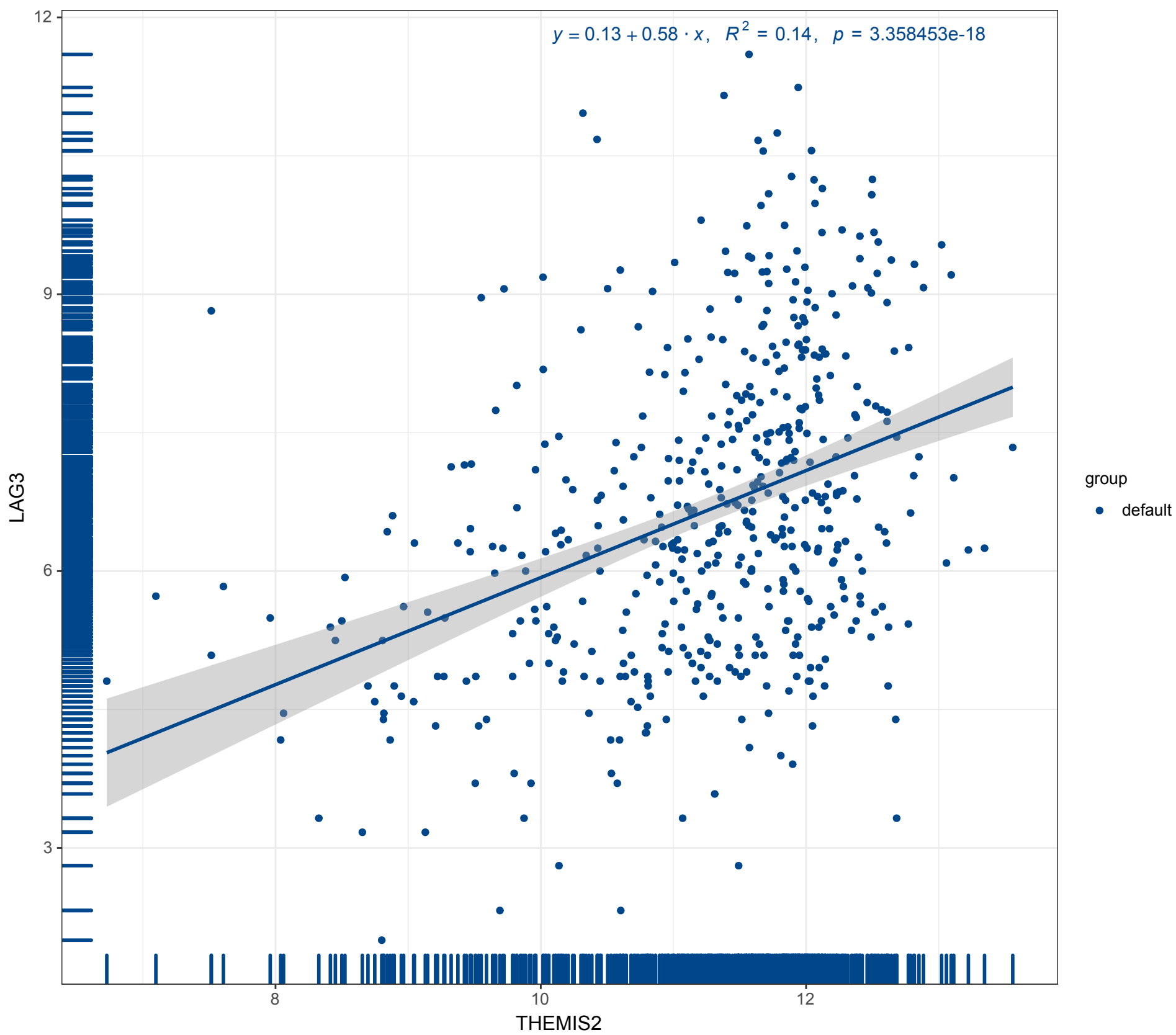

Supplement: Supplementary file 1 — Supplementary Information. [file 41598_2024_58943_MOESM1_ESM.zip › Raw data/Raw data/7. 免疫检查点分析/相关性分析/8.pdf]

Line Regreusion Plot

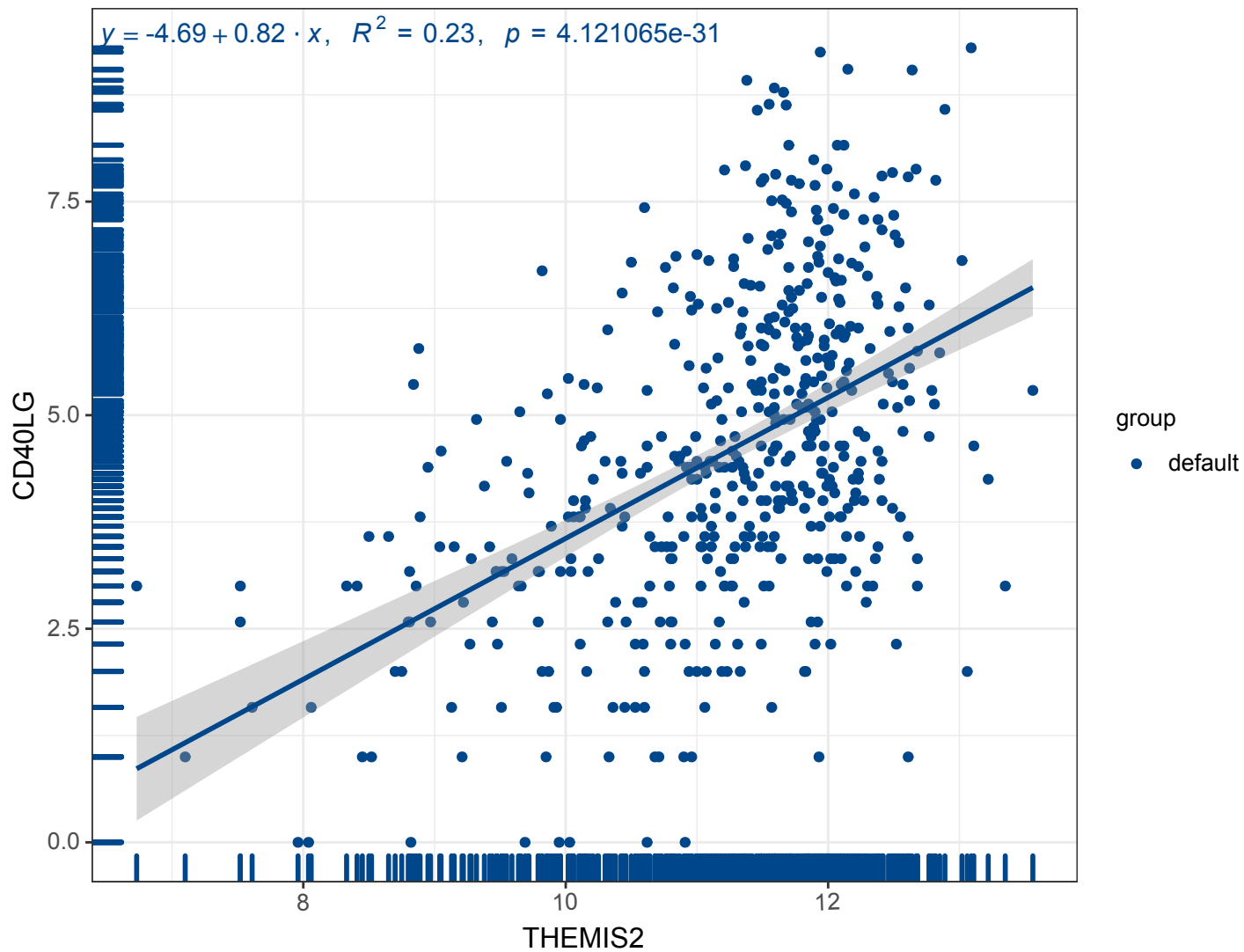

Supplement: Supplementary file 1 — Supplementary Information. [file 41598_2024_58943_MOESM1_ESM.zip › Raw data/Raw data/7. 免疫检查点分析/相关性分析/9.pdf]

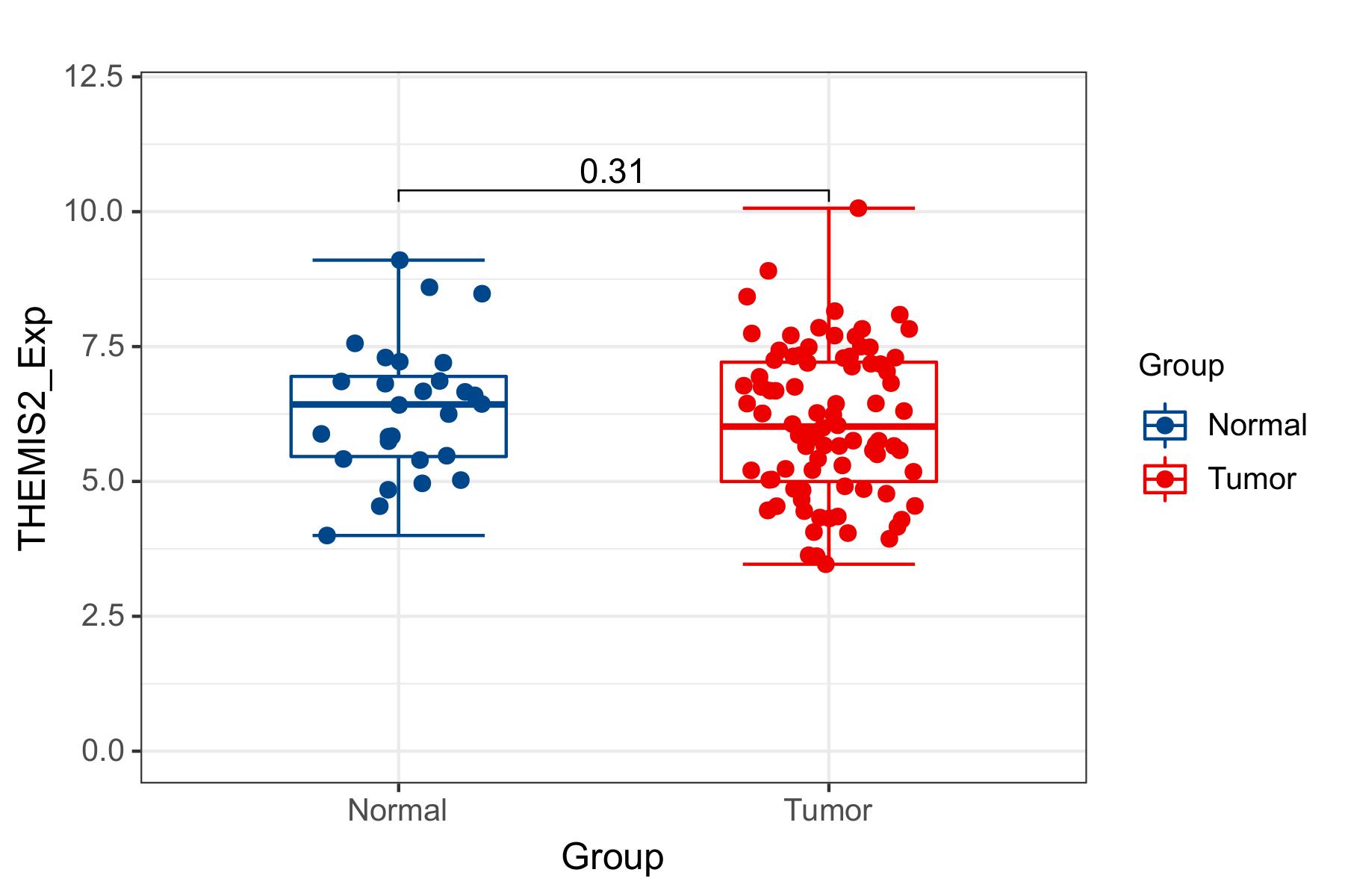

Supplement: Supplementary file 1 — Supplementary Information. [file 41598_2024_58943_MOESM1_ESM.zip › Raw data/Raw data/GEO-THEMIS2_expression.jpg]

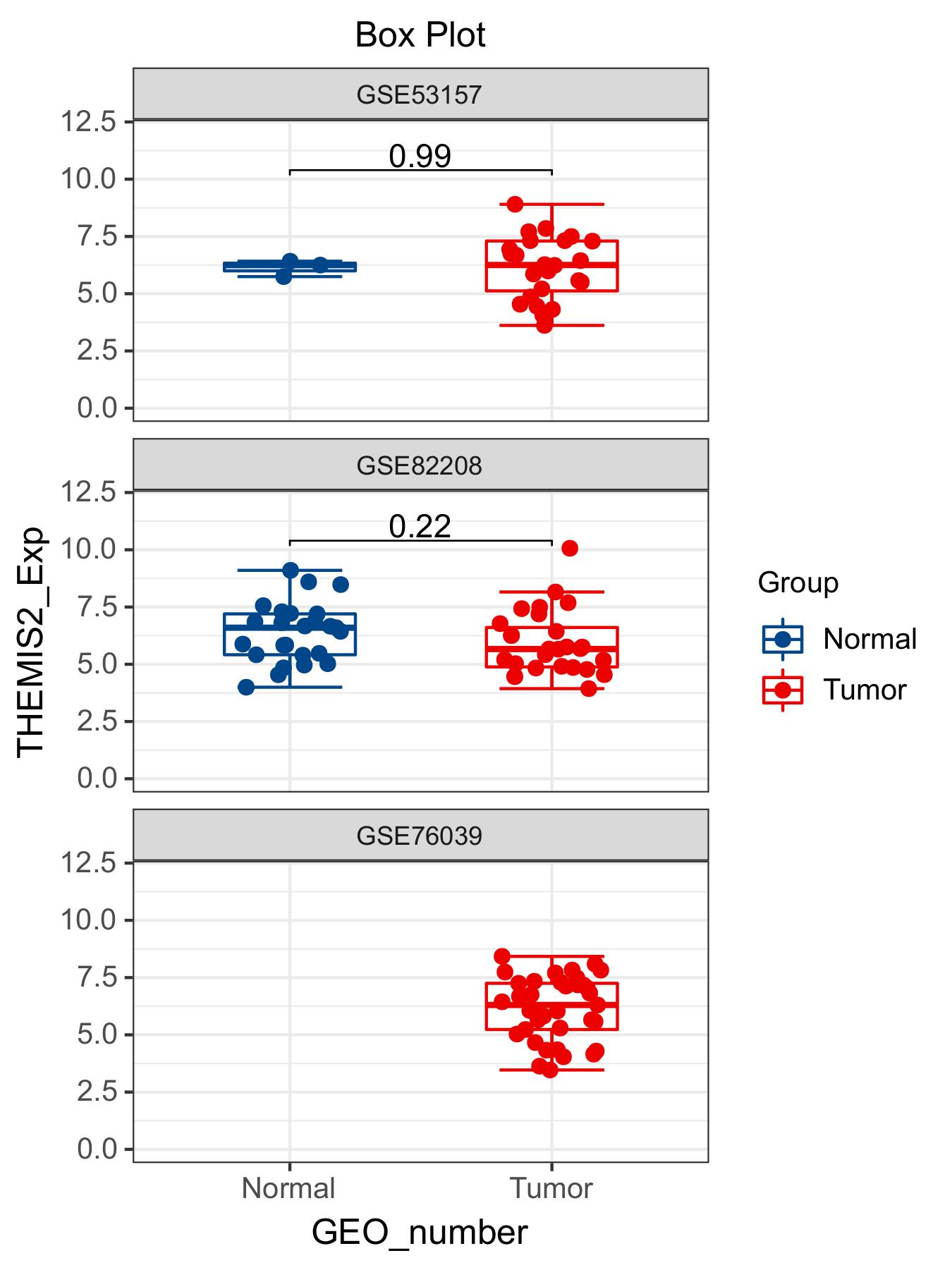

Supplement: Supplementary file 1 — Supplementary Information. [file 41598_2024_58943_MOESM1_ESM.zip › Raw data/Raw data/GEO-three-THEMIS2_expression.jpg]

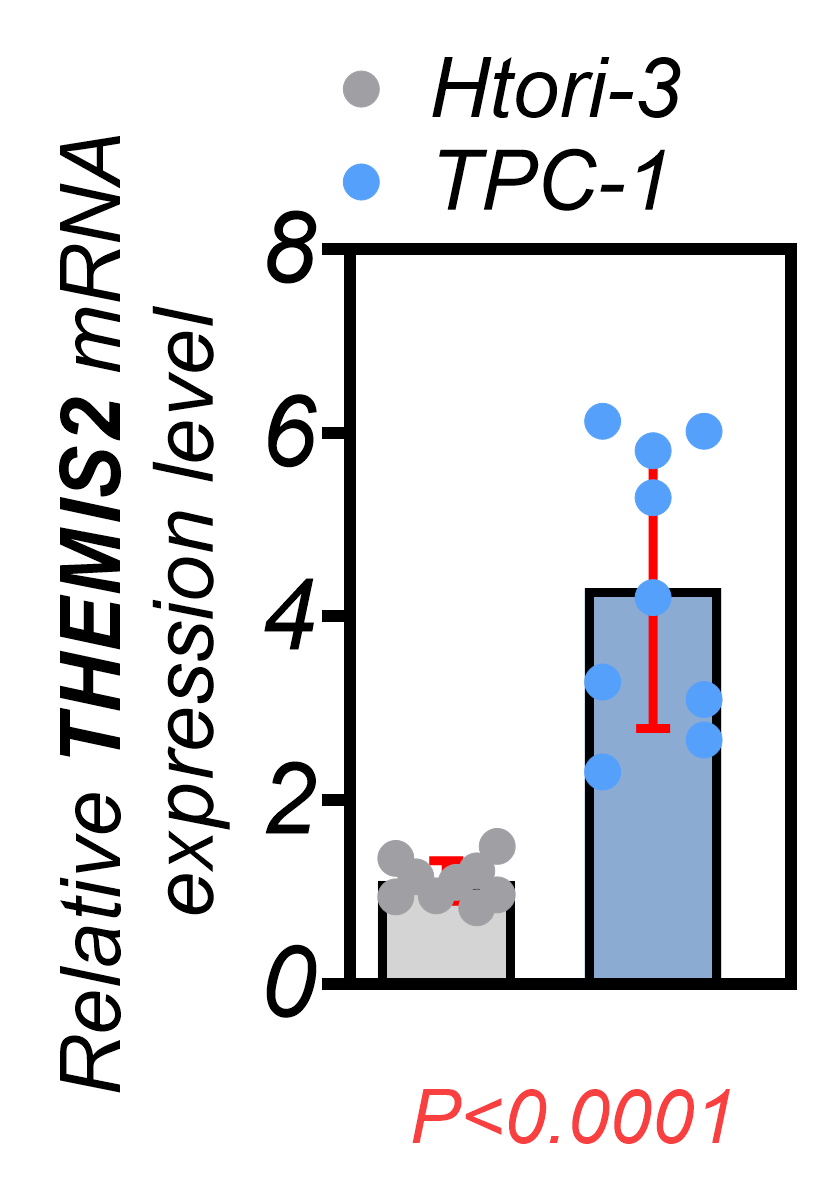

Supplement: Supplementary file 1 — Supplementary Information. [file 41598_2024_58943_MOESM1_ESM.zip › Raw data/Raw data/RT-PCR.tif]
