# Supplementary material for: Comprehensive bioinformatics analysis unveils THEMIS2 as a carcinogenic indicator related to immune infiltration and prognosis of thyroid cancer
Source: Sci Rep. 2024 Apr 8;14:8156. doi: 10.1038/s41598-024-58943-6 (PMC11001958; doi:10.1038/s41598-024-58943-6)
Supplement: Supplementary file 2 — Supplementary Table S1. [file 41598_2024_58943_MOESM2_ESM.docx]

**Table S1 Information for the three GEO datasets included in the current study**

| Reference | Dataset | Platform | Number of samples (Tumor/Control) |
| --- | --- | --- | --- |
| (Chen chen et al., 2023) | GSE53157 | GPL570 [HG-U133_Plus_2] Affymetrix Human Genome U133 Plus 2.0 Array | 27(24/3) |
| (Martin C Nwadiugwu, 2020) | GSE82208 | GPL570 [HG-U133_Plus_2] Affymetrix Human Genome U133 Plus 2.0 Array | 52 (27/25) |
| (Liang jiang et al., 2022) | GSE76039 | GPL570 [HG-U133_Plus_2] Affymetrix Human Genome U133 Plus 2.0 Array | 37 (37) |
